# Physician Reluctance to Intervene in Addiction: A Systematic Review

**DOI:** 10.1001/jamanetworkopen.2024.20837

**Published:** 2024-07-17

**Authors:** Melinda Campopiano von Klimo, Laura Nolan, Michelle Corbin, Lisa Farinelli, Jarratt D. Pytell, Caty Simon, Stephanie T. Weiss, Wilson M. Compton

**Affiliations:** 1 JBS International, Inc, North Bethesda, Maryland; 2 National Institute on Drug Abuse, National Institutes of Health, Bethesda, Maryland; 3 Department of Medicine, University of Colorado School of Medicine, Aurora, Colorado; 4 National Survivors Union, Greensboro, North Carolina; 5 NC Survivors Union, Greensboro, North Carolina; 6 Whose Corner Is It Anyway, Holyoke, Massachusetts

## Abstract

**Importance:**

The overdose epidemic continues in the US, with 107 941 overdose deaths in 2022 and countless lives affected by the addiction crisis. Although widespread efforts to train and support physicians to implement medications and other evidence-based substance use disorder interventions have been ongoing, adoption of these evidence-based practices (EBPs) by physicians remains low.

**Objective:**

To describe physician-reported reasons for reluctance to address substance use and addiction in their clinical practices using screening, treatment, harm reduction, or recovery support interventions.

**Data Sources:**

A literature search of PubMed, Embase, Scopus, *medRxiv*, and SSRN Medical Research Network was conducted and returned articles published from January 1, 1960, through October 5, 2021.

**Study Selection:**

Publications that included physicians, discussed substance use interventions, and presented data on reasons for reluctance to intervene in addiction were included.

**Data Extraction and Synthesis:**

Two reviewers (L.N., M.C., L.F., J.P., C.S., and S.W.) independently reviewed each publication; a third reviewer resolved discordant votes (M.C. and W.C.). This systematic review was conducted according to the Preferred Reporting Items for Systematic Reviews and Meta-analyses guidelines and the theoretical domains framework was used to systematically extract reluctance reasons.

**Main Outcomes and Measures:**

The primary outcome was reasons for physician reluctance to address substance use disorder. The association of reasons for reluctance with practice setting and drug type was also measured. Reasons and other variables were determined according to predefined criteria.

**Results:**

A total of 183 of 9308 returned studies reporting data collected from 66 732 physicians were included. Most studies reported survey data. Alcohol, nicotine, and opioids were the most often studied substances; screening and treatment were the most often studied interventions. The most common reluctance reasons were lack of institutional support (173 of 213 articles [81.2%]), knowledge (174 of 242 articles [71.9%]), skill (170 of 230 articles [73.9%]), and cognitive capacity (136 of 185 articles [73.5%]). Reimbursement concerns were also noted. Bivariate analysis revealed associations between these reasons and physician specialty, intervention type, and drug.

**Conclusions and Relevance:**

In this systematic review of reasons for physician reluctance to intervene in addiction, the most common reasons were lack of institutional support, knowledge, skill, and cognitive capacity. Targeting these reasons with education and training, policy development, and program implementation may improve adoption by physicians of EBPs for substance use and addiction care. Future studies of physician-reported reasons for reluctance to adopt EBPs may be improved through use of a theoretical framework and improved adherence to and reporting of survey development best practices; development of a validated survey instrument may further improve study results.

## Key Points

**Question:**

What reasons do physicians give for not addressing substance use and addiction in their clinical practice?

**Findings:**

In this systematic review of 283 articles, the institutional environment (81.2% of articles) was the most common reason given for physicians not intervening in addiction, followed by lack of skill (73.9%), cognitive capacity (73.5%), and knowledge (71.9%).

**Meaning:**

These findings suggest effort should be directed at creating institutional environments that facilitate delivery of evidence-based addiction care while improving access to both education and training opportunities for physicians to practice necessary skills.

## Teaser

This systematic review evaluates physician-reported reasons for reluctance to address substance use and addiction in their clinical practices.

## Introduction

Overdose is a leading cause of injury-related death in the US,^1^ with 107 941 such deaths occurring in 2022^2^ and annual deaths due to alcohol exceeding 140 000 from 2015 to 2019.^3^ The more than 46.3 million people in the US with a past-year substance use disorder^4^ and a nationwide economic impact of alcohol misuse and illicit drug use that tops $442 billion^5^ further evidences the magnitude of this crisis.

A variety of safe and effective evidence-based practices (EBPs) to identify, reduce the morbidity and mortality of, and treat substance use disorders exist. Examples include screening, brief intervention, and referral to treatment,^6,7,8,9,10^ as well as behavioral therapies and pharmacotherapies for nicotine, alcohol, and opioid use disorders.^11,12,13^ Furthermore, harm reduction approaches (eg, naloxone training and coprescribing, drug checking and testing, and syringe service programs) offer significant individual and public health benefits for people who use drugs and for those who do not have abstinence-based treatment goals.^14,15,16^

Clinician adoption of EBPs is necessary; however, screening for substance use disorders remains low,^7^ creating missed opportunities to intervene in harmful substance use or recognize and discuss potential progression to a severe disorder. Treatment capacity is inadequate to meet demand,^17^ with only 6.3% of people with a past-year substance use disorder receiving treatment in the US in 2021.^4^ Our goal is to summarize published data on physician-described barriers to adoption of EBPs for addiction in clinical practice and recommend actions to address them.

## Methods

### Data Sources and Searches

This systematic review was conducted according to the Preferred Reporting Items for Systematic Reviews and Meta-analyses (PRISMA) reporting guidelines. The search strategy was developed iteratively with a National Library of Medicine informationist specializing in systematic reviews. We applied this strategy on October 4, 2021, to PubMed, Embase, and Scopus and on October 5, 2021, to *medRxiv* and SSRN Medical Research Network. In addition, a gray literature search of relevant government and nongovernment websites was conducted on October 5, 2021. We found no previous similar systematic reviews. The systematic review protocol was registered in PROSPERO (CRD42022286208) and accepted on January 14, 2022.

### Study Selection

A 12-person team used Covidence to apply exclusion criteria first to the title and abstract of each study then to the full text of studies not already excluded. Two people (L.N., M.C., L.F., J.P., C.S., and S.W.) reviewed each study in both rounds. Discordant opinions were resolved by a third reviewer (M.C. and W.C.). To be included, the study had to present data on: (1) physicians at any practice level; (2) any substance use intervention(s) (Box); and (3) physician reasons for reluctance to intervene in addiction. Studies not in English, letters, editorials, narrative reviews, and commentaries were excluded. Data collection on reasons for reluctance were systemized using the theoretical domains framework (TDF),^18,19^ a comprehensive approach for identifying behavioral determinants and for assessing implementation problems (eg, clinicians’ behavior) to inform intervention development. The team created a data extraction template with 10 reluctance reason categories (Box). We did not formally assess risk of bias in included studies because few used experimental or controlled study designs. Due to patterns observed during data extraction, the team approved the ad hoc collection of data on factors (eg, using a theoretical framework, obtaining target audience input in survey design, and piloting surveys) that could affect the internal validity of individual studies or precision of results. We conducted a limited exploration of facilitators because we observed that many included studies provided at least some data on possible facilitators of intervention in addiction.

Box. **Definitions of Intervention Type and Reluctance Reasons**

**Intervention type and definition**

Harm reduction: syringe services, overdose prevention, naloxone, or drug user health.
Screening and assessment: screening, assessment of positive screening, or diagnosis.
Treatment: brief intervention, medication management, or behavioral services.
Recovery support: care coordination, care integration, or relapse prevention.

**Reason and definition^a^**

Knowledge: beliefs about having the necessary knowledge, awareness, or understanding, including knowledge of condition or scientific rationale, procedural knowledge, or knowledge of task environment.
Institutional environment: beliefs about support from institution or employer, including material resources, organizational culture, competing demands.
Skills: beliefs about having the necessary skills, ability, or proficiency to deliver the intervention.
Cognitive capacity: beliefs about the cognitive capacity to manage a level of expected complexity of care, possibly related to cognitive overload and mental fatigue.
Expectation of benefit: beliefs about the likelihood of the patient benefiting or the course of the disease being altered due to the intervention.
Social influences: beliefs about public or community acceptance or support for the intervention, including willingness to allocate or develop needed resources.
Emotion: feelings of fear, dislike, worry, negative judgement, worthiness of patient population.
Relationship: concern about harming or losing the patient-physician relationship by causing offense, provoking avoidance, or other negative consequence.
Reinforcement: beliefs about the adequacy of reimbursement, professional rewards, and other positive reinforcement.
Professional role/identity: beliefs about professional role, boundaries, and group identity, excluding the intervention.

^a^
Reasons are derived from the theoretical domains framework, a comprehensive approach for identifying behavioral determinants and assessing implementation problems (eg, clinicians’ behavior) to inform intervention development.

### Data Analysis

We conducted a series of quantitative analyses using SPSS, version 27 (IBM). Analyses were selected based on their purpose; independent variable; dependent variable; and statistical requirements, including measurement levels. We examined reasons for reluctance by specialty, intervention, drug type, and year and common combinations of reasons for reluctance using bivariate analysis and cross-tabulation. We conducted a regression analysis of reasons for reluctance by year. Statistical significance was considered a 2-sided *P* value less than .05. The exploratory analyses of ad hoc study quality data were not part of the planned analysis and are descriptive only. We used Atlas.ti version 24 (Atlas.ti) to conduct thematic analysis to examine facilitators using the following themes: knowledge and skills, intrapersonal and interpersonal factors, infrastructure, and regulation reform.

## Results

### Study Characteristics

Our search yielded 9308 studies published between January 1, 1960, and October 5, 2021, with 1280 remaining after removal of duplicates and 552 assessed for eligibility (eFigure 1 in Supplement 1). Of 283 studies^20,21,22,23,24,25,26,27,28,29,30,31,32,33,34,35,36,37,38,39,40,41,42,43,44,45,46,47,48,49,50,51,52,53,54,55,56,57,58,59,60,61,62,63,64,65,66,67,68,69,70,71,72,73,74,75,76,77,78,79,80,81,82,83,84,85,86,87,88,89,90,91,92,93,94,95,96,97,98,99,100,101,102,103,104,105,106,107,108,109,110,111,112,113,114,115,116,117,118,119,120,121,122,123,124,125,126,127,128,129,130,131,132,133,134,135,136,137,138,139,140,141,142,143,144,145,146,147,148,149,150,151,152,153,154,155,156,157,158,159,160,161,162,163,164,165,166,167,168,169,170,171,172,173,174,175,176,177,178,179,180,181,182,183,184,185,186,187,188,189,190,191,192,193,194,195,196,197,198,199,200,201,202,203,204,205,206,207,208,209,210,211,212,213,214,215,216,217,218,219,220,221,222,223,224,225,226,227,228,229,230,231,232,233,234,235,236,237,238,239,240,241,242,243,244,245,246,247,248,249,250,251,252,253,254,255,256,257,258,259,260,261,262,263,264,265,266,267,268,269,270,271,272,273,274,275,276,277,278,279,280,281,282,283,284,285,286,287,288,289,290,291,292,293,294,295,296,297,298,299,300,301,302^ included (eTable 1 in Supplement 1), 97.30% were published in 2000 or later (Table 1). The number of studies increased over time. For example, 4 studies^89,156,184,236^were published in 2000 and 21^33,48,49,66,68,75,77,79,93,107,108,113,139,142,148,240,251,255,302,306,313^ in 2021, with a high of 31 ^8,27,47,50,52,54,69,74,92,100,114,121,146,147,161,165,174,182,191,193,199,204,206,209,221,247,263,270,275,287,300^ in 2020 (eTable 2, eTable 3, eTable 4, eTable 5, and eFigure 2 in Supplement 1). Together, the included studies describe the views of 66 732 physicians who largely practiced general practice, internal medicine, or family medicine primarily in an office setting in the US. Most studies reported survey-based research results. Of the 4 general categories of addiction interventions (Table 2), treatment was most often addressed, followed by screening and assessment, with harm reduction and recovery support least discussed. Some studies addressed more than 1 intervention. Alcohol (86 studies^20,21,23,25,26,29,31,34,36,38,41,44,51,53,54,57,59,60,62,69,70,71,72,81,82,86,88,89,94,95,103,105,111,113,117,119,123,124,125,126,127,131,132,138,141,150,153,155,158,160,162,164,168,170,171,173,176,191,192,193,196,197,198,199,200,201,204,205,210,219,235,237,248,250,254,256,258,271,281,283,285,291,294,296,299,300^), nicotine (30 studies^28,40,48,49,52,61,73,85,97,109,118,129,134,140,142,149,179,188,190,212,218,223,231,249,252,265,270,286,288,298^), and opioids (104 studies^30,32,33,35,37,42,46,47,50,55,56,58,64,66,74,75,76,77,78,79,80,83,84,87,90,91,92,98,99,100,104,106,107,108,110,112,114,115,121,122,130,133,135,137,139,143,144,146,147,148,151,152,154,156,163,165,167,172,174,180,182,184,186,189,202,203,206,207,213,214,215,216,221,222,225,226,227,228,238,239,240,242,243,244,245,247,251,253,255,257,259,262,269,272,275,277,280,282,284,287,290,292,293,302^) were most often studied alone. Among studies reporting on multiple drugs (44 studies^22,39,43,45,63,65,67,68,93,96,101,102,116,120,136,145,166,181,183,185,194,195,208,209,217,220,230,232,233,234,241,246,260,263,264,267,268,273,274,278,279,289,295,297^), alcohol was included most often (38 studies^45,63,65,67,68,93,96,101,102,116,120,136,145,166,181,183,194,195,208,209,217,230,232,233,234,241,246,260,264,267,268,273,274,278,279,289,295,297^). Other substances were often reported as “other” or merely “drugs.” Cross-tabulations of each reason for reluctance with each of the most common specialties, interventions, and drugs produced no significant results; consequently, no *P* values are reported (Table 2). While this systematic review is of physician reluctance, 110 studies^20,23,24,25,28,30,31,33,34,39,42,44,47,48,50,52,54,57,59,63,64,66,67,68,69,70,87,88,90,92,93,95,99,101,103,104,105,106,107,109,111,112,113,116,120,122,123,126,129,134,136,138,139,143,146,147,151,156,157,159,162,166,167,169,173,174,177,178,183,186,189,190,192,194,195,199,200,201,203,205,206,209,211,217,221,225,229,235,236,243,244,245,251,257,260,261,266,269,270,275,277,280,283,286,287,290,291,297,299,302^ mentioned possible facilitators of physician engagement.

**Table 1.** Characteristics of Included Studies

Characteristic	Studies, No. (%) (N = 283)
Publication characteristic	
Year of publication^a^	
1960-1989	14 (4.95)
1990-1999	21 (7.4)
2000-2009	66 (23.3)
2010-2021	181 (64.0)
Study methods	
Survey based	170 (60.1)
Qualitative	82 (29.0)
Mixed-methods	27 (9.5)
Other^b^	4 (1.4)
Physician characteristic	
Specialty (top 4)^c^	
General practice/primary care physician	110 (38.9)
Internal medicine	80 (28.3)
Family medicine	67 (23.7)
General psychiatry	55 (19.4)
Country (top 4)^c^	
United States	168 (59.4)
United Kingdom	20 (7.1)
Canada	16 (5.7)
Australia	15 (5.3)
Practice setting (top 4)^c^	
Office	148 (52.3)
Inpatient	49 (17.3)
Emergency department	41 (14.5)
Addiction treatment	11 (3.9)
Level of practice^c^	
Attending	236 (83.4)
Trainee	58 (20.5)
Unspecified	31 (11.0)
Intervention type^c^	
Treatment	235 (83.0)
Screening	153 (54.1)
Harm reduction	82 (29.0)
Recovery support	27 (9.5)
Drug type (top 3)	
Alcohol	124 (43.8)
Opioids	120 (42.4)
Nicotine	43 (15.2)

^a^ This number is lower than the total number of publications because 1 article was missing information on the publication year.

^b^ This category includes 2 clinical studies and 2 meta-analyses.

^c^ This number will be higher than the total number of publications, and the proportion will be higher than 100% because many publications reported data on multiple physician characteristics. Other specialties included, but were not limited to, emergency medicine, pediatrics, addiction medicine, obstetrics and gynecology, surgery, infectious disease, and addiction psychiatry. Other countries included, but were not limited to Germany, Sweden, the Netherlands, Italy, Spain, Belgium, and Finland. Other practice settings were coded as other or not specified.

**Table 2.** Summary of Results Reasons for Reluctance by Specialty, Intervention, and Drug

Result	Studies citing reason		No./total No. (%)									
	All studies citing reason, (N = 283), No. (%)	Studies citing reason when asked, No./total No. (%)^a^	By specialty^a^^,^^b^			By intervention^a^^,^^c^				By drug^a^^,^^d^		
			General practice/ primary care	Family medicine	Internal medicine	Harm reduction	Screening	Treatment	Recovery support	Alcohol	Nicotine	Opioids
Reason for reluctance^e^												
Knowledge	174 (61.5)	174/242 (71.9)	32/56 (57.1)	4/5 (80.0)	11/12 (91.7)	14/18 (77.8)	7/12 (58.3)	53/67 (79.1)	1/2 (50.0)	55/79 (69.6)	18/28 (64.3)	62/80 (77.5)
Institutional environment	173 (61.1)	173/213 (81.2)	39/55 (70.9)	4/5 (80.0)	6/8 (75.0)	15/19 (78.9)	8/12 (66.7)	57/62 (91.9)	0	44/57 (77.2)	12/20 (60.0)	83/91 (91.2)
Skills	170 (60.1)	170/230 (73.9)	38/56 (67.9)	4/5 (80.0)	10/11 (90.9)	15/20 (75.0)	7/12 (58.3)	44/60 (73.3)	2/2 (100)	51/70 (72.9)	19/28 (67.9)	60/78 (76.9)
Cognitive capacity	136 (48.1)	136/185 (73.5)	38/50 (76.0)	4/4 (100)	2/4 (50.0)	14/18 (77.8)	11/13 (84.6)	33/50 (66.0)	0	41/54 (75.9)	17/ 21 (81.0)	53/73 (72.6)
Expectation of benefit	132 (46.6)	132/215 (61.4)	31/53 (58.5)	3/6 (50.0)	6/6 (100)	11/18 (61.1)	7/13 (53.8)	34/59 (57.6)	1/1 (100)	49/66 (74.2)	23/25 (92.0)	32/77 (41.6)
Social influences	121 (42.8)	121/184 (65.8)	27/46 (58.7)	3/4 (75.0)	3/5 (60.0)	8/17 (47.1)	9/12 (75.0)	30/48 (62.5)	0	36/51 (70.6)	10/18 (55.6)	49/78 (62.8)
Emotion	118 (41.7)	118/199 (59.3)	22/48 (45.8)	4/5 (80.0)	7/7 (100)	10/16 (62.5)	3/12 (25.0)	43/58 (74.1)	2/2 (100)	28/53 (52.8)	6/20 (30.0)	61/82 (74.4)
Clinician-patient relationship	91 (32.2)	91/164 (55.5)	27/42 (64.3)	1/3 (33.3)	2/4 (50.0)	7/14 (50.0)	12/14 (85.7)	8/35 (22.9)	0	35/59 (59.3)	13/19 (68.4)	17/51 (33.3)
Reinforcement	79 (27.9)	79/165 (47.9)	21/47 (44.7)	3/4 (75.0)	2/4 (50.0)	4/11 (36.4)	2/10 (20.0)	26/49 (53.1)	0	20/45 (44.4)	7/16 (43.8)	32/65 (49.2)
Professional role/identity	80 (28.3)	80/203 (39.4)	18/52 (34.6)	1 /4 (25.0)	5/7 (71.4)	5/16 (31.3)	5/13 (38.5)	25/53 (47.2)	0	31/63 (49.2)	11/26 (42.3)	27/72 (37.5)

^a^ Studies did not gather or report data on all reasons for reluctance within each category. No./total No. indicates the number of studies that asked about the reason for physician reluctance/the number of studies that cited the reason for physician reluctance for corresponding specialty, intervention, or drug. χ^2^ Statistics were calculated between each reason for reluctance and specialty, intervention, and drug subcategories. No comparisons were significant. Consequently, no *P* values are reported here.

^b^ This refers to publications where the reason for reluctance, when asked, was cited by the top 3 specialties.

^c^ This refers to publications where the reason for reluctance, when asked, was cited by intervention.

^d^ Publications where the reason for reluctance, when asked, was cited by top 3 drugs.

^e^ Reasons for reluctance definitions: publications met the criteria as having asked about the reason for reluctance, either through interview or focus group questions or in survey questions.

### Physician Reluctance

Most studies did not gather or report data on all reasons. When queried, institutional environment (173 of 213 articles [81.2%]^20,22,25,26,27,30,31,32,33,35,37,38,40,41,42,43,44,46,47,49,50,51,54,55,56,57,58,59,60,61,62,63,64,66,68,74,75,76,77,78,80,82,83,84,86,87,89,90,91,92,93,95,97,99,100,104,106,107,108,109,110,112,113,114,116,117,121,122,123,124,126,127,129,134,135,136,137,138,139,143,144,146,147,148,150,151,153,154,155,157,158,159,161,162,163,164,165,167,169,170,171,172,173,174,175,176,179,180,182,183,185,186,189,192,195,198,199,201,202,203,204,206,207,209,211,216,217,218,219,220,221,223,226,228,229,230,232,233,234,236,238,239,241,242,243,245,247,251,252,257,258,259,260,261,263,264,265,268,269,271,272,275,277,280,284,287,290,291,293,295,299,301,302^) was the most common reason, followed by lack of skill (170 of 230 articles [73.9%]^20,21,22,24,25,26,27,28,29,30,31,32,33,35,37,38,39,47,48,49,51,53,54,55,58,59,61,63,64,65,66,67,68,75,76,78,80,81,82,84,85,88,89,91,92,93,95,97,98,99,100,102,103,104,105,106,107,109,110,111,112,113,114,116,117,118,119,120,121,123,124,125,130,131,132,134,136,138,139,142,143,145,147,149,150,152,154,159,160,161,167,168,172,173,174,176,178,180,182,183,186,188,190,191,193,194,197,198,199,200,201,202,204,206,207,208,209,210,211,213,214,216,218,219,220,221,224,225,226,229,231,233,235,236,238,241,242,246,247,249,256,259,264,265,266,268,269,271,273,274,276,277,278,279,281,282,283,285,286,287,290,291,292,293,294,295,297,298,301,302^), cognitive capacity (136 of 185 articles [73.5%]^22,25,26,30,32,34,37,40,41,47,48,49,52,55,58,59,60,61,63,64,65,66,68,69,71,74,75,77,78,80,82,85,87,88,89,90,91,93,95,97,100,101,104,105,106,107,109,110,111,112,113,114,116,117,119,120,122,123,124,125,126,129,134,135,136,138,139,142,146,147,148,149,150,151,154,155,156,159,160,161,162,167,172,174,180,181,185,186,187,190,191,192,196,197,198,199,205,206,209,211,213,214,216,217,219,225,229,230,231,232,235,237,239,241,242,243,254,256,260,264,265,268,269,270,272,275,277,283,286,287,290,291,292,299,301,302^), and knowledge (174 of 242 articles [71.9%]^20,21,22,25,26,27,28,29,30,31,32,33,36,37,39,42,43,49,53,54,55,56,57,58,59,61,62,64,65,66,68,69,70,73,76,78,81,82,84,85,91,92,93,95,97,98,99,100,102,103,104,105,106,107,109,110,113,114,116,117,118,119,120,121,126,128,130,131,136,138,139,141,142,143,147,149,150,151,152,154,155,157,159,160,161,163,166,167,168,170,171,172,173,174,176,177,178,179,180,182,183,184,185,186,188,190,191,192,193,194,197,198,199,200,201,202,203,204,206,207,208,209,210,212,213,214,215,219,221,224,226,236,237,238,241,242,244,246,247,251,252,256,257,258,264,266,267,268,269,271,273,274,276,277,278,279,280,281,283,284,285,286,287,288,292,293,294,295,297,298,299,300,301,302^); and social influences (121 of 184 articles [65.8%]^26,27,30,31,32,41,42,46,47,49,51,57,58,60,62,63,68,71,77,79,80,82,83,88,90,92,95,99,101,102,106,107,108,109,110,112,113,114,118,121,122,123,124,126,127,129,134,135,136,137,138,146,147,151,153,155,157,158,159,161,165,167,169,170,176,177,180,182,185,189,195,197,198,199,200,201,202,203,204,205,206,207,208,210,211,212,216,217,219,221,223,227,228,233,234,235,238,242,245,247,249,254,255,257,260,261,264,266,268,269,282,283,286,287,289,291,296,297,298,301,302^) (Table 2). We conducted bivariate analyses of reasons for reluctance and specialty, drug type, intervention, and time (Table 2; eFigure 3 in Supplement 1). Too few studies of recovery support existed to conduct a bivariate analysis with reasons for reluctance. Analysis of combinations of the top 4 reasons for reluctance found the most often paired reluctance reasons were knowledge and skill (135 of 221 articles [61.1%]^20,21,22,25,26,27,28,29,30,31,32,33,37,39,49,53,54,55,58,59,61,64,65,66,68,76,78,81,82,84,85,91,92,93,95,97,98,99,100,102,103,104,105,106,107,109,110,113,114,116,117,118,119,120,121,130,131,136,138,139,142,143,147,149,150,152,154,159,160,161,167,168,172,173,174,176,178,180,182,183,186,188,190,191,193,194,197,198,199,200,201,202,204,206,207,208,209,210,213,214,219,221,224,226,236,238,241,242,246,247,256,264,266,268,269,271,273,274,276,277,278,279,281,283,285,286,287,292,293,294,295,297,298,301,302^), followed by cognitive capacity and institutional environment (99 of 165 articles [60.0%]^22,25,26,30,32,37,40,41,47,49,55,58,59,60,61,63,64,66,68,74,75,77,78,80,82,87,89,90,91,93,95,97,100,104,106,107,109,110,112,113,114,116,117,122,123,124,126,129,134,135,136,138,139,146,147,148,150,151,154,155,159,161,162,167,172,174,180,185,186,192,198,199,206,209,211,216,217,219,229,230,232,239,241,242,243,260,264,265,268,269,272,275,277,287,290,291,299,301,302^) (Table 3). Institutional environment appeared in combination with other reasons more often than any other reason (7 of 12 pairings). Reasons not in our data extraction template were described in a few studies, including lack of demand (13 articles^87,92,112,122,143,167,171,214,216,232,257,280,292^), cost to the patient (8 articles^58,69,148,155,171,174,288,292^), and patient refusal (6 articles^61,146,170,174,182,206^). Analysis of the trend over time for each reason for reluctance revealed a significant increase in identification of social influence (F_1,20_ = 4.91; *P* = .04) and relationship (F_1,20_ = 4.54; *P* = .046) (eFigure 3 in Supplement 1). We extracted exemplar text from included studies for the top 4 reasons for reluctance (Table 4), discussed in the following section.

**Table 3.** Frequency of Reasons for Reluctance Identified in Combination

Combinations of reasons for reluctance	Citing reason when asked, out of all publications that studied each reason, No./total No. (%)
Knowledge and skills	135/221 (61.1)
Cognitive capacity and institutional environment	99/165 (60.0)
Skills and institutional environment	106/179 (59.2)
Knowledge and institutional environment	107/186 (57.5)
Skills and cognitive capacity	96/169 (56.8)
Social influence and institutional environment	95/172 (55.2)
Knowledge and cognitive capacity	82/165 (49.7)
Skills, knowledge, and institutional environment	84/174 (48.3)
Institutional environment and emotion	75/165 (45.5)
Expectation of benefit and institutional environment	78/174 (44.8)
Expectation of benefit and skills	81/181 (44.8)
Expectation of benefit and knowledge	80/187 (42.8)

**Table 4.** Top 4 Reasons for Reluctance Examples

Top 4 reasons for reluctance and exemplars	Sources
Institutional environment	
Regulatory and liability concerns	Lowenstein et al,^167^ 2019; Sullivan et al,^259^ 2006; Oros et al,^107^ 2021; Taylor et al,^261^ 2003; Green et al,^90^ 2014; Fraeyman et al,^76^ 2016; Foti et al,^75^ 2021; Schulte et al,^245^ 2013; Martino et al,^174^ 2019; Logan et al,^165^ 2020; Lin and Detels,^163^ 2011; H ernandez-Meier et al,^99^ 2019; Gordon et al,^87^ 2008; Chenworth et al,^50^ 2020; Arfken et al,^35^ 2010; Andrilla et al,^32^ 2017
Lack of trained staff	Taylor et al,^260^ 2007; Savage and Ross,^242^ 2022; Netherland et al,^207^ 2009; McMurphy et al,^186^ 2006; McCausland et al,^182^ 2020; Lowenstein et al,^167^ 2019; Lacroix et al,^154^ 2018; Dong et al,^66^ 2021
Lack of acceptance of addiction interventions by leadership	Taylor et al,^261^ 2003; Thomas et al,^57^ 2003; Matheson et al,^175^ 2010; Mark, Kranzler, Poole et al,^155^ 2003; Lambrechts et al,^169^ 2015; Gano et al,^80^ 2018; Bounthavong et al,^275^ 2020
Cost to the patient or lack of insurance coverage	McCausland et al,^182^ 2020; Mark, Kranzler, Poole et al,^155^ 2003; Martino et al,^174^ 2019; Martinez et al,^173^ 2016; Mark, Kranzler, Song^171^ 2003; Mark, Kranzler, Song, et al,^170^ 2003; Kohan et al,^148^ 2021
Lack of clinician backup	Green et al,^90^ 2014; Fraeyman et al,^76^ 2016; Foti et al,^75^ 2021; DeFlavio et al,^64^ 2015; Cunningham et al,^56^ 2007; Cotter et al,^54^ 2020
Medication unavailability at pharmacies	Mark, Kranzler, Song, et al,^170^ 2003; Kohan et al,^148^ 2021; Kissin et al,^144^ 2006; Harris et al,^95^ 2013
Lack of resources to train staff	Hawk et al,^221^ 2020; Haffajee et al,^92^ 2020; Cunningham et al,^59^ 2010
Physician reimbursement insufficient to cover both the staff time necessary to intervene in addiction and the expense of additional staff training	Netherland et al,^207^ 2009; Martino et al,^174^ 2019; Binswanger et al,^277^ 2015
Record keeping or confidentiality concerns	Netherland et al,^207^ 2009; Sullivan et al,^259^ 2006; Bounthavong et al,^275^ 2020
Absence of population-specific patient education materials	Taylor et al,^260^ 2007; Wilson et al,^291^ 2011
Lack of acceptance of addiction interventions by staff	Sullivan et al,^259^ 2006; Oros et al,^107^ 2021
Nonexistent or unimplemented treatment algorithms	Hernandez-Meier et al,^99^ 2019; Webster et al,^287^ 2020
Lack of staff time required for prior authorizations	Haffajee et al,^92^ 2020
Contractual limitations	Wilson et al,^291^ 2011
Mental health programs not accepting patients with addiction	Todd et al,^264^ 2002
Addiction treatment programs rejecting patients deemed insufficiently ready to change or having difficulty matching the level of care needed	Rahm et al,^229^ 2015
Difficulty obtaining records from addiction treatment programs	Oros et al,^107^ 2021
Medicaid reimbursement specifically highlighted as inadequate	McMurphy et al,^186^ 2006
Physicians perceived the reimbursement to be inadequate but were not certain of the reimbursed amount	Cunningham et al,^56^ 2007
Lack of knowledge	
Knowledge was more deficient for treatment than for screening or diagnosis and for drug use more than for alcohol or tobacco use	Hawk et al,^221^ 2020; Hernandez-Meier et al,^99^ 2019; Wakeman et al,^273^ 2013; Midmer et al,^194^ 2011; Kunins et al,^152^ 2009; Johansson et al,^117^ 2002; Herzig et al,^102^ 2006; Hammond et al,^93^ 2021; El-Shahawy et al,^252^ 2016; Elliott et al,^70^ 2006; Demmert et al,^65^ 2011; Aalto et al,^20^ 2001
Physicians unfamiliar with the evidence for SUDs as biomedical conditions	Stöver,^257^ 2011; Kersnik et al,^138^ 2009; Joudrey et al,^199^ 2020; Johansson et al,^119^ 2005
Unfamiliar with harm reduction strategies	Lacroix et al,^154^ 2018; Tesema et al,^58^ 2018
Unfamiliar with substance use screening	Cunningham et al,^59^ 2010; Levy et al,^161^ 2020
Physicians lacked awareness of the extent of substance use in their patients	Donovan,^256^ 1991
Lack of skill	
Lack of skills to conduct interventions effective enough to produce behavior change, including counseling	Cunningham et al,^59^ 2010; Wilson et al,^291^ 2011; Johansson et al,^117^ 2002; Chichetto et al,^51^ 2019; Beich et al,^38^ 2002; Aalto et al,^21^ 2006
Lack of skill needed to initiate or manage treatment, especially for SUDs other than alcohol or tobacco	Hawk et al,^221^ 2020; Haffajee et al,^92^ 2020; Wakeman et al,^273^ 2013; Midmer et al,^194^ 2011; Kunins et al,^152^ 2009; Deehan et al,^63^ 1997
Lack of experience with observing or delivering an SUD intervention under supervision	Foti et al,^75^ 2021; Donovan,^256^ 1991; Gunderson et al,^91^ 2006; Gatewood et al,^238^ 2016; Abed and Neira-Munoz,^22^ 1990
Lack of skills to conduct brief intervention	Rahm et al,^229^ 2015; Hammond et al,^93^ 2021; Ordean et al,^209^ 2020
Inabilities to assemble or demonstrate naloxone administration devices	Tesema et al,^58^ 2018; Binswanger et al,^277^ 2015
Inability to deliver appropriate training in its use to patients	Hernandez-Meier et al,^99^ 2019
Lack of cognitive capacity	
Intervening in addiction as too time-consuming, both during the appointment and for monitoring	Green et al,^90^ 2014; Gordon et al,^87^ 2008; Webster et al,^287^ 2020; Hammond et al,^93^ 2021; Ehrie et al,^69^ 2020
Need to prioritize patients’ competing needs	Oros et al,^107^ 2021; Tesema et al,^58^ 2018; Van Hook et al,^268^ 2007; Omole et al,^109^ 2014
A general sense of overwhelm with clinical tasks (eg, “just too busy”)	Wilson et al,^291^ 2011; DeFlavio et al,^43^ 2015
Delegating screening to other clinical team members was viewed as diverting time from the physician visit	Rahm et al,^229^ 2015
Available tools were considered time-consuming	Taylor et al,^260^ 2007

Abbreviation: SUD, substance use disorder.

### Institutional Environment

Reasons for reluctance related to the institutional environment included lack of trained staff^66,154,167,182,186,207,242,260^ or resources to train staff,^59,92,221^ acceptance of addiction interventions by staff^107,259^ or leadership,^57,80,155,169,175,261,275^ and clinician backup.^54,56,64,75,76,90^ Regulatory and liability concerns were frequently reported,^32,35,50,75,76,87,90,99,107,163,165,167,174,245,259,261^ as were record-keeping or confidentiality concerns^207,259,275^ and staff time required for prior authorizations.^92^ Often mentioned were also cost to the patient or lack of insurance coverage,^148,155,170,171,173,174,182^ along with medication unavailability at pharmacies^95,144,148,170^ and the absence of population-specific patient education materials.^260,291^ Less frequently cited but noteworthy reasons for reluctance include contractual limitations,^291^ nonexistent or unimplemented treatment algorithms,^99,287^ mental health programs not accepting patients with addiction,^264^ addiction treatment programs rejecting patients deemed insufficiently ready to change or having difficulty matching the level of care needed,^229^ and difficulty obtaining records from addiction treatment programs.^107^ Reimbursement can be viewed as a component of institutional environment. In the TDF, reimbursement is 1 part of reinforcement as a reason for reluctance (Box). While reinforcement was 1 of the 2 least often identified reasons for reluctance, data specific to reimbursement was extracted because it is a perennial point of concern in adopting evidence-based interventions for addiction. Physician reimbursement was viewed as insufficient to cover both the staff time necessary to intervene in addiction and the expense of additional staff training.^174,207,277^ Medicaid reimbursement was specifically highlighted as inadequate.^186^ In some cases, physicians perceived the reimbursement to be inadequate but were not certain of the reimbursed amount.^56^

### Lack of Knowledge

In studies identifying lack of knowledge as a reason for reluctance, knowledge was more deficient for treatment than for screening or diagnosis and for drug use more than for alcohol or tobacco use.^20,65,70,93,99,102,117,152,194,221,252,273^ Physicians were unfamiliar with the evidence for substance use disorders as biomedical conditions,^119,138,199,257^ harm reduction strategies,^58,154^ and screening for risky substance use.^59,161^ Some physicians lacked awareness of the extent of substance use by their patients.^256^

### Lack of Skill

Physicians reported lacking skills to conduct interventions effective enough to produce behavior change, including counseling^21,38,51,59,117,291^ and brief intervention.^93,209,229^ They also described a lack of skill needed to initiate or manage treatment,^92,152,221,273^ especially for substance use disorders other than alcohol or tobacco.^63,194^ In some studies, they equated their lack of skill with lack of experience with observing or delivering a substance use disorder intervention under supervision.^22,75,91,238,256^ Inabilities to assemble or demonstrate naloxone administration devices^58,277^ or to deliver appropriate training in its use to patients^99^ were also noted.

### Lack of Cognitive Capacity

Lack of cognitive capacity was not often characterized beyond a general sense of overwhelm with clinical tasks (eg, “just too busy”)^64,291^ and the need to prioritize patients’ competing needs.^58,107,109,268^ In some cases, physicians perceived intervening in addiction as too time-consuming, both during the appointment and for monitoring,^69,87,90,93,287^ or that addiction treatment demand would be too great.^66,75,91^ Even delegating screening to other clinical team members was viewed as diverting time from the physician visit^229^; available tools were considered time-consuming.^260^ Some physicians expected meeting the care needs of patients with addiction to be too time-consuming.

### Facilitators

We analyzed 4 main themes related to facilitators. First, physicians need the knowledge and skills to intervene; they need adequate education and training in areas like managing pharmacology. Second, intrapersonal and interpersonal factors exist that facilitate physician intervention. Intrapersonal factors include physician characteristics (eg, work experience, confidence, and practice type) and motivation (eg, desire to improve patient outcomes, reimbursement, and understanding addiction as within their scope of practice). Interpersonal factors include the physician-patient relationship, specifically the patient characteristics that may compel the physician to intervene (eg, the patient is receptive to help). Third, an infrastructure is needed that supports physician interventions and includes institutional changes at the practice level to implement protocols to standardize care (eg, screening and improved technology). An environment that fosters collaboration with other professionals or entities (eg, multidisciplinary teams and referral systems) and offers resources that would support the intervention (eg, materials or tools for use with patients, follow-up care, or treatment facilities) is also essential. Finally, regulation reforms (eg, eliminating prior authorization requirements, expanding substance use disorder insurance coverage, and simplifying laws and policies governing prescribing and medication distribution to patients) would facilitate physician intervention.

## Discussion

The number and growth of publications meeting inclusion criteria for this systematic review demonstrates increasing interest in the perceived and actual barriers to physician engagement with addiction in clinical practice. The significant increase in social influence and relationship as reasons for reluctance over time may indicate increased awareness of stigma and associated social harms. Regarding intervention types, the availability of effective alcohol use disorder and opioid use disorder pharmacotherapies likely accounts for the literature’s focus on those therapies, corresponding with efforts to increase access to medications for opioid use disorder and to promote the adoption of screening, brief intervention, and referral to treatment practices. As the evidence base for a wider array of harm reduction strategies grows, it will be important to understand and address physicians’ perceived and actual barriers to their acceptance and adoption of those strategies. Information is limited on the adoption of recovery support interventions by physicians, a finding that also merits investigation.

That institutional environment is associated with physician reluctance to intervene may not surprise practicing clinicians. The pairing of institutional environment and cognitive capacity may signify the cost in time physicians expend overcoming institutional barriers to EBP for addiction (eg, inefficient workflows and communication and coordination of care across silos). The association of institutional environment with treatment and opioids may reflect the push to increase buprenorphine access despite regulatory impediments and health systems being unprepared for this responsibility.

Strategies to reduce physician reluctance related to institutional environment include greater commitment by health systems to make essential workflow and staffing changes, the breaking down of barriers between addiction services and both medical and mental health care, and commitment by insurers to provide reimbursement that covers the actual cost of providing addiction interventions. The analysis of facilitators supports a specific need for protocols to adequately intervene with patients with either at-risk substance use or substance use disorders. Institutional environment changes (eg, investing in staffing and staff training, implementing standard practices or protocols, and conducting addiction-specific quality assurance) could also facilitate intervention.

Lack of knowledge and skill are top reasons for reluctance, both separately and combined. It is unclear whether survey respondents understood knowledge and skill as the researchers intended because these terms were rarely defined in the studies. Only a few studies allowed for future replication by including objective measures of knowledge or skill (eg, counting successfully delivered services and interviewing patients).

True lack of knowledge and skill can be understood in several ways, including as a manifestation of the volume of information practicing clinicians are required to possess, acquire, and update. For example, physicians need updated information on dosing, pharmacology, and overall efficacy of interventions and medications. This challenge is made harder if interventions (eg, screening practices, initiating pharmacotherapy) are insufficiently adapted for different practice settings. Delivering these interventions effectively, efficiently, and in a nonstigmatizing manner requires skill mastery. Physicians, like other clinicians, acquire their skills by observing and then practicing under supervision. Medical education and postgraduate training have only recently begun to prepare physicians for these tasks.^303,304^

Ongoing training is critical for physicians to acquire and apply advanced skills in the care of this patient population,^305,306,307^ but few opportunities exist to observe and be observed practicing new skills once required medical training is complete. The analysis of facilitators suggests skill training should focus on brief intervention (eg, screening or assessment) and on communication with patients. Trainings accessible to physicians (eg, free or incentivized, hands-on, or delivered in clinical settings) and delivered by specialized trainers and/or mentors would facilitate the growth of a pool of experts to intervene in substance use. Physicians who expand their knowledge and skills should be eligible for continuing medical education credits and increased compensation.

Other reasons for reluctance (eg, negative social influences, negative emotions toward people who use drugs, and fear of harming the relationship with the patient by discussing substance use) could each be viewed as manifestations of stigma associated with substance use disorder and its treatment. Lack of demand may also reflect stigma if it is a manifestation of unwillingness on the part of patients to seek help due to fear of social, legal, and moral judgement or a presumption by the physician that there is no addiction in their community.

These reasons may diminish if effective public and professional education, in particular those developed and led by patient groups or by people who use drugs,^308,309,310,311,312^ are delivered to counter stigma.^313^ The analysis of facilitators suggests the following may be helpful: educational materials for patients and families, community outreach, and public health campaigns promoting nonstigmatizing language.

Reducing stigma will not be enough to address fear of harming the patient relationship, especially for physicians who care for minors and other populations that may be subject to punitive consequences of addiction. These physicians must consider additional confidentiality requirements, and their fear of harming the patient by triggering negative social and legal consequences may be more of a deterrent than previously considered. Interpersonal aspects of the patient-physician relationship and how they create reluctance or facilitate intervention are not well understood, although the analysis of facilitators shows that physicians may be motivated to intervene in substance use disorders when they have an established relationship with the patient, the patient is receptive to help, and/or the desire to improve patient outcomes is strong. Future research should examine unintended impacts of increased physician intervention in addiction like strain on the physician-patient relationship, less opportunity to meet other health care needs, and stigmatizing interactions with other health care clinicians due to the substance use disorder diagnosis being more widely documented.

### Limitations

This study has limitations. Inconsistent use of terms across included studies increased the complexity and interpretation of this analysis, but analysis of a sample this size can still inform research and policy. Studies were often developed without the benefit of a theoretical framework. Survey development lacked or failed to report participation of the audience of focus and/or was not piloted, raising concerns about the validity and applicability of results. During the years this systematic review covered, new medications and formulations became available, making comparison across decades challenging. The unregulated drug market also evolved, resulting in changes to illicit substances, methods of using them, and the regulatory environment in which clinicians address substance use. This review was limited to physicians, some of whom may have participated in more than 1 survey or focus group in the included studies. Although the results are relevant to the practice environment of many clinicians, including those specializing in addiction, they do not reflect the unique challenges that may be encountered by specific disciplines. Although we collected and described data about facilitators, the original search was not designed specifically to retrieve publications about facilitators of intervention in addiction.

## Conclusions

These data suggest that policy, regulatory, or accreditation changes are needed to systematically address institutional barriers, as well as increases to physician reimbursement and opportunities for clinically relevant training that provides both skill development and knowledge gain. Another systematic review of facilitators and reluctance among other clinical disciplines may refine the recommendations presented here. Future studies of clinician reluctance to adopt EBPs for addiction need to be of higher quality. They, at a minimum, should employ a theoretical framework and adhere to survey development best practices or use a validated survey instrument.

## Notes

Supplement 1. **eFigure 1.** Preferred Reporting Items for Systematic Reviews and Meta-Analyses Flowchart **eFigure 2. **Linear Model Results: Additional Tables and Figures for Examining Reasons for Reluctance Over Time, When Asked **eFigure 3. **Distribution of Publications by Year **eTable 1.** Study Characteristics and Quality Data **eTable 2. **Distribution of Publications by Year **eTable 3. **Physician Practice Settings Reported in Publications Since 2010 **eTable 4. **Drug Types Reported Since 2000 **eTable 5. **Count and Percentage of Reasons for Reluctance From 2000 to 2021, When Asked
eReferences

Supplement 2.
Data Sharing Statement
