# Supplementary material for: Physician Reluctance to Intervene in Addiction: A Systematic Review
Source: JAMA Netw Open. 2024 Jul 17;7(7):e2420837. doi: 10.1001/jamanetworkopen.2024.20837 (PMC11255913; doi:10.1001/jamanetworkopen.2024.20837)
Supplement: Supplement 1. — eFigure 1. Preferred Reporting Items for Systematic Reviews and Meta-Analyses Flowchart eFigure 2. Linear Model Results: Additional Tables and Figures for Examining Reasons for Reluctance Over Time, When Asked eFigure 3. Distribution of Publications by Year eTable 1. Study Characteristics and Quality Data eTable 2. Distribution of Publications by Year eTable 3. Physician Practice Settings Reported in Publications Since 2010 eTable 4. Drug Types Reported Since 2000 eTable 5. Count and Percentage of Reasons for Reluctance From 2000 to 2021, When Asked eReferences [file jamanetwopen-e2420837-s001.pdf]

Supplemental Online Content

Campopiano von Klimo M, Nolan L, Corbin M, et al. Physician reluctance to intervene in addiction: a systematic review. *JAMA Netw Open*. 2024;7(7):e2420837. doi:10.1001/jamanetworkopen.2024.20837

eFigure 1. Preferred Reporting Items for Systematic Reviews and Meta-Analyses Flowchart

eFigure 2. Linear Model Results: Additional Tables and Figures for Examining Reasons for Reluctance Over Time, When Asked

eFigure 3. Distribution of Publications by Year

eTable 1. Study Characteristics and Quality Data

eTable 2. Distribution of Publications by Year

eTable 3. Physician Practice Settings Reported in Publications Since 2010

eTable 4. Drug Types Reported Since 2000

eTable 5. Count and Percentage of Reasons for Reluctance From 2000 to 2021, When Asked

eReferences

This supplemental material has been provided by the authors to give readers additional information about their work.

© 2024 Campopiano von Klimo M et al. *JAMA Network Open.*

eFigure 1. Preferred Reporting Items for Systematic Reviews and Meta-Analyses Flowchart

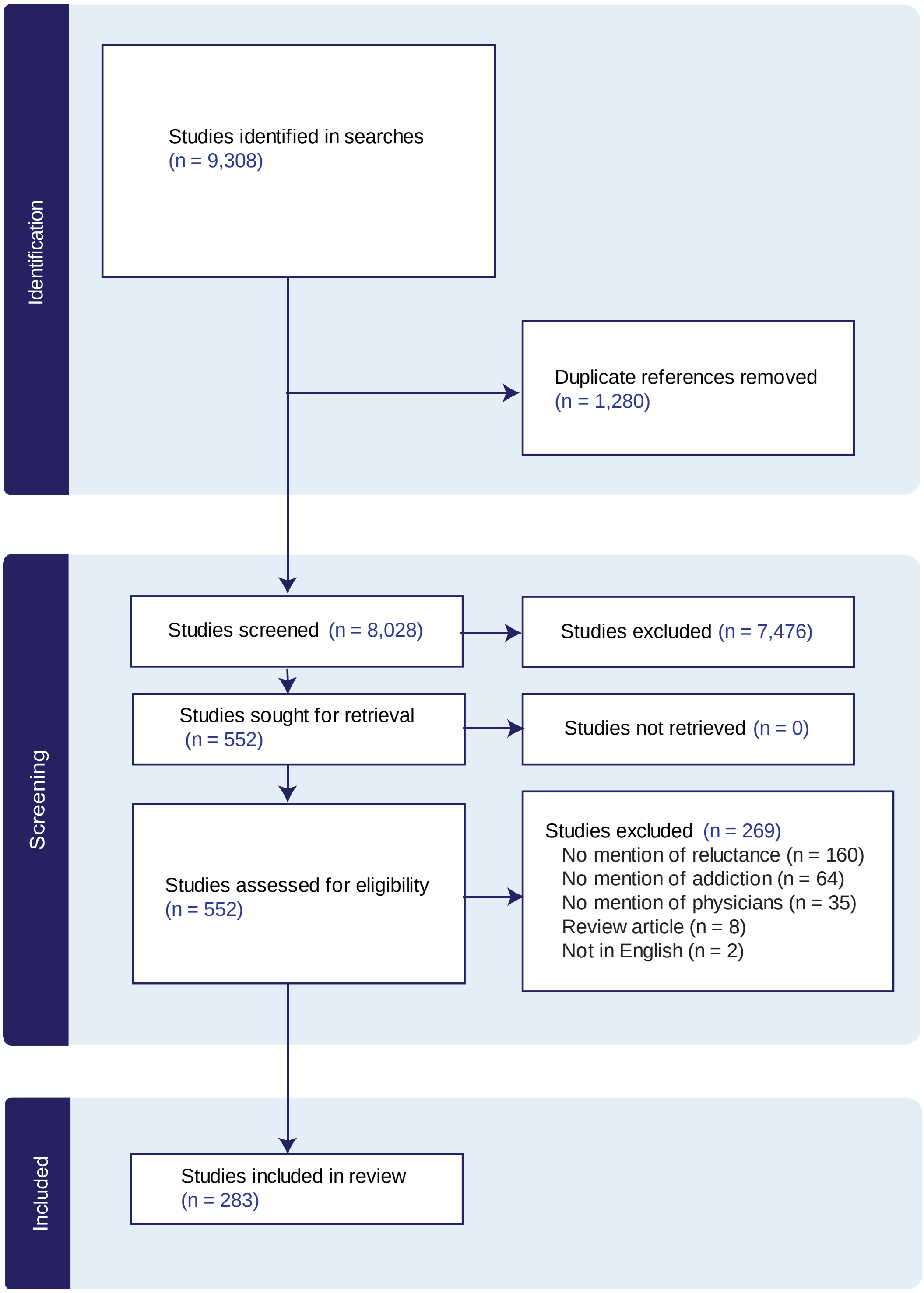

© 2024 Campopiano von Klimo M et al. *JAMA Network Open.*

eFigure 2. Linear Model Results: Additional Tables and Figures for Examining Reasons for Reluctance Over Time, When Asked

Knowledge	
F	P-value
.524	.478

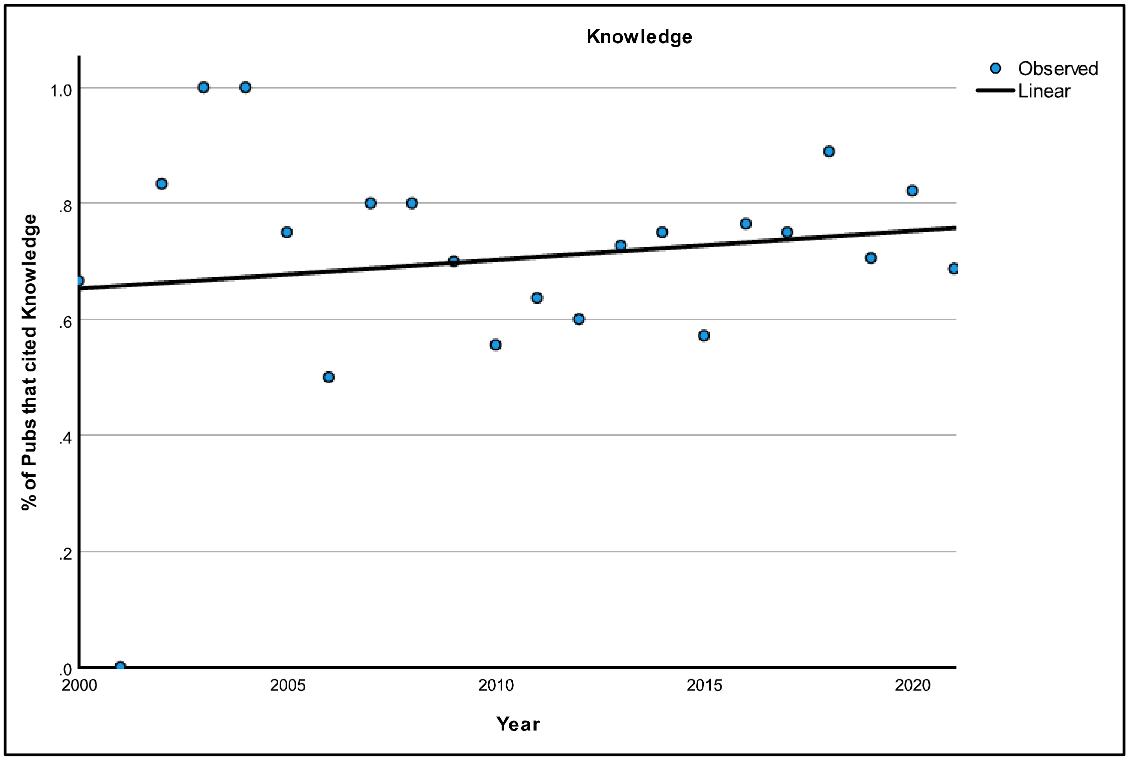

© 2024 Campopiano von Klimo M et al. *JAMA Network Open.*

Skills	
F	P-value
.861	.365

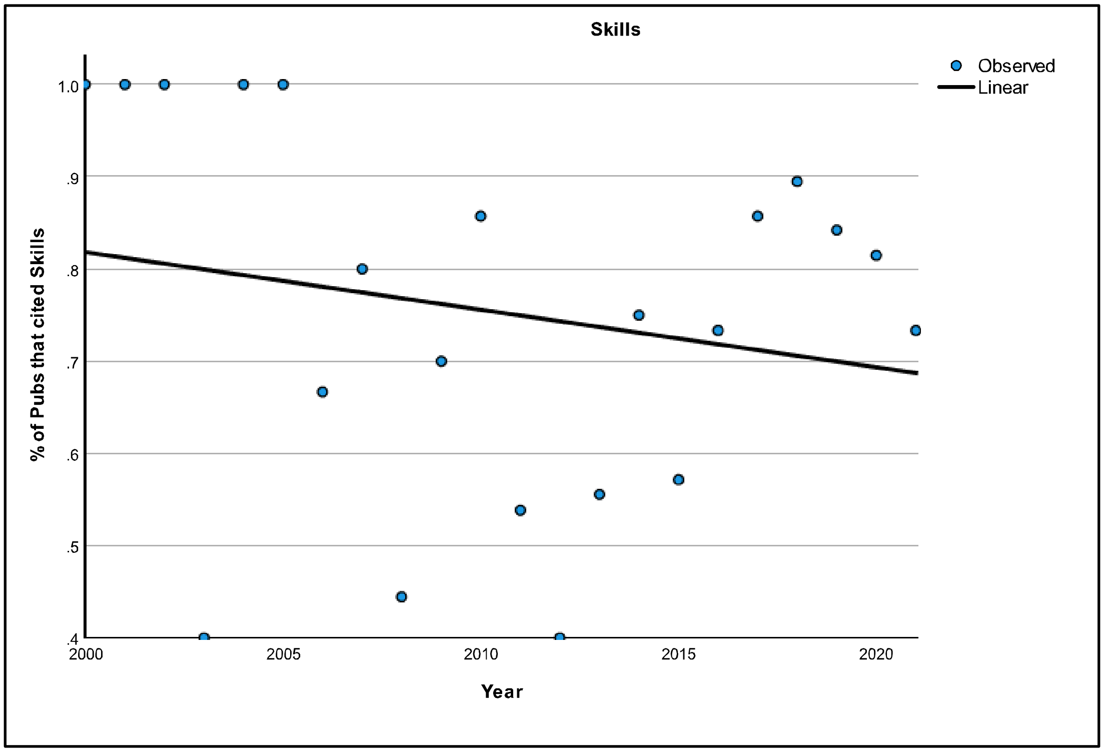

Professional Role/Identity	
F	P-value
**1.654**	**.213**

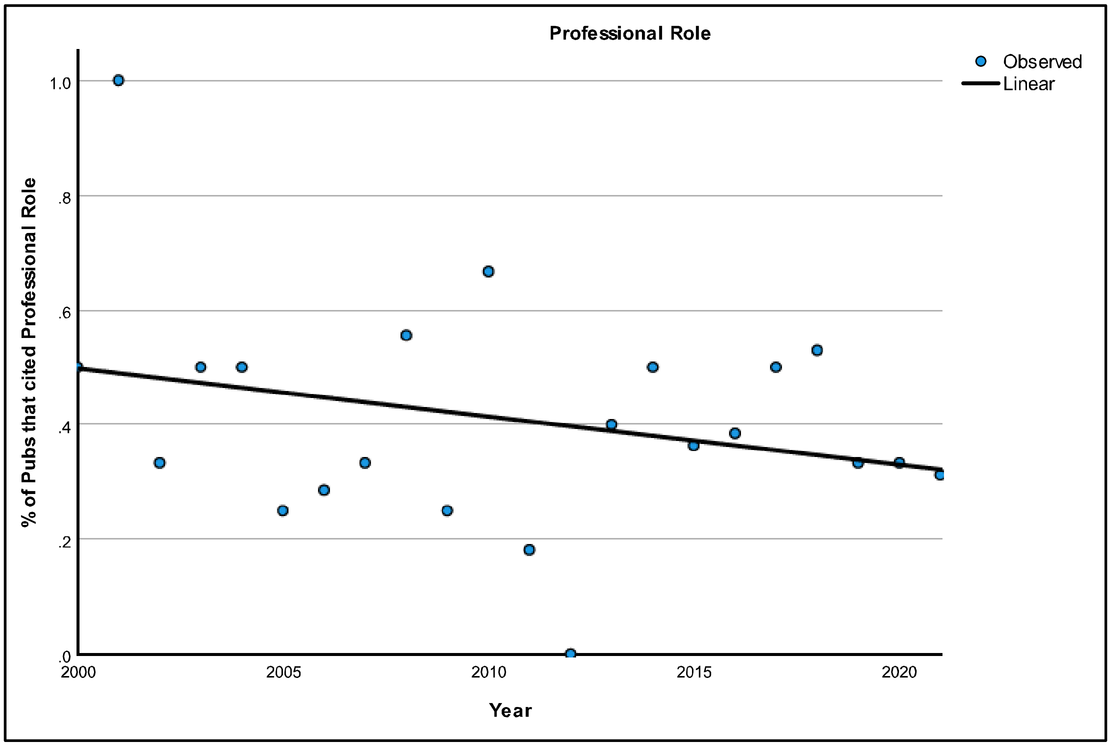

© 2024 Campopiano von Klimo M et al. *JAMA Network Open.*

Expectation of Benefit	
F	P-value
.148	.705

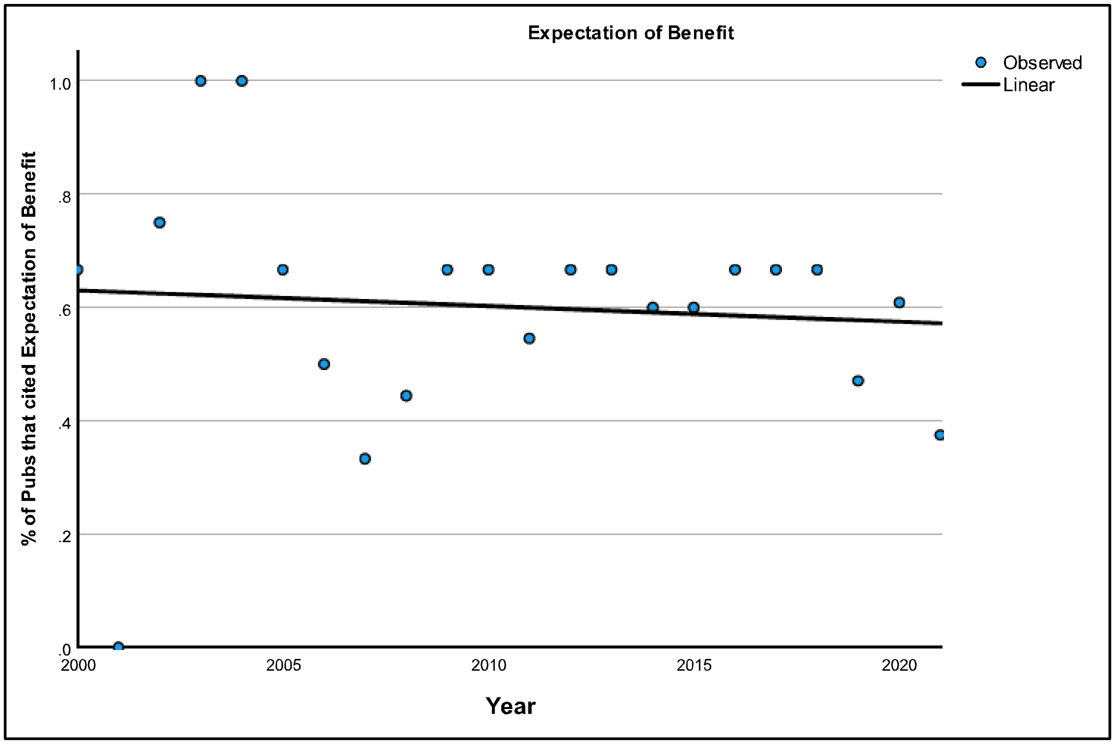

© 2024 Campopiano von Klimo M et al. *JAMA Network Open.*

Reinforcement	
F	P-value
.025	.875

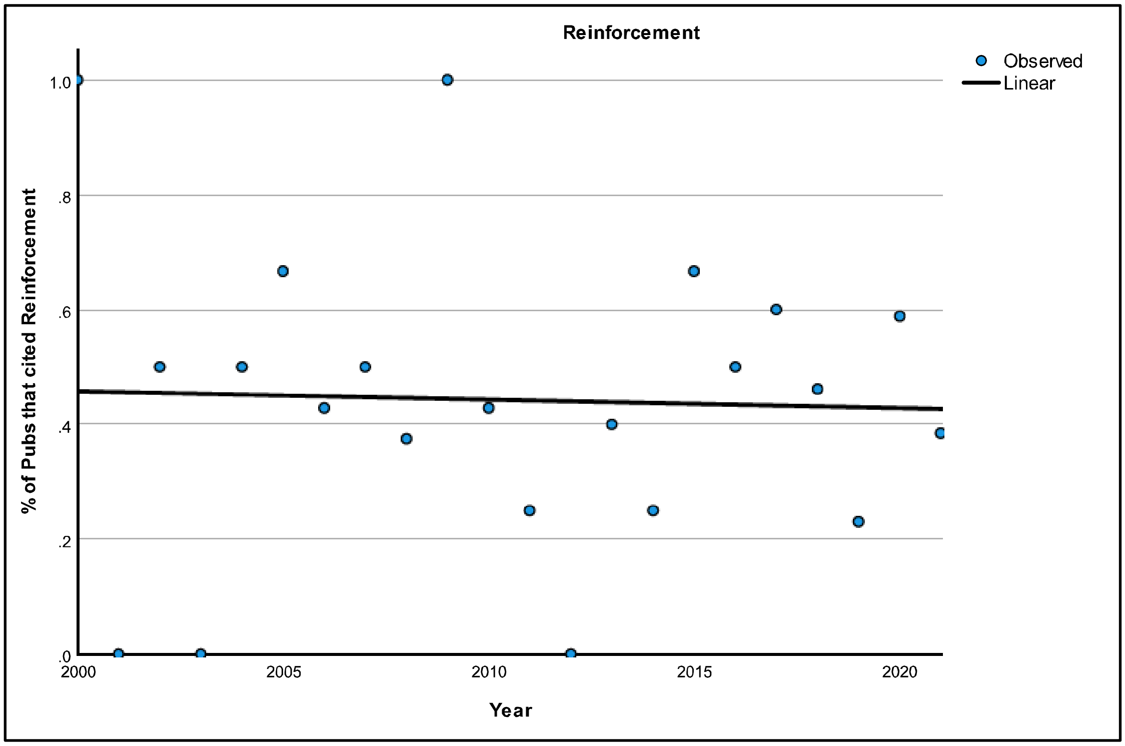

© 2024 Campopiano von Klimo M et al. *JAMA Network Open.*

Social Influences	
F	P-value
4.910*	.038

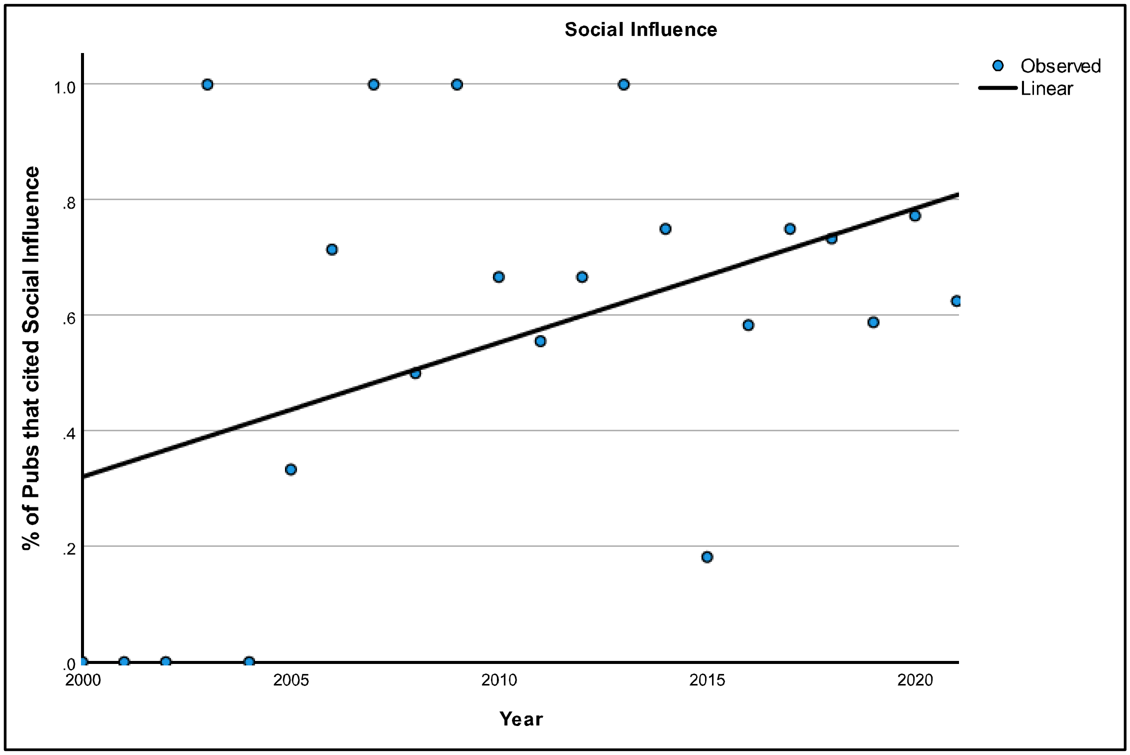

© 2024 Campopiano von Klimo M et al. *JAMA Network Open.*

Institutional Environment	
F	P-value
3.439	.078

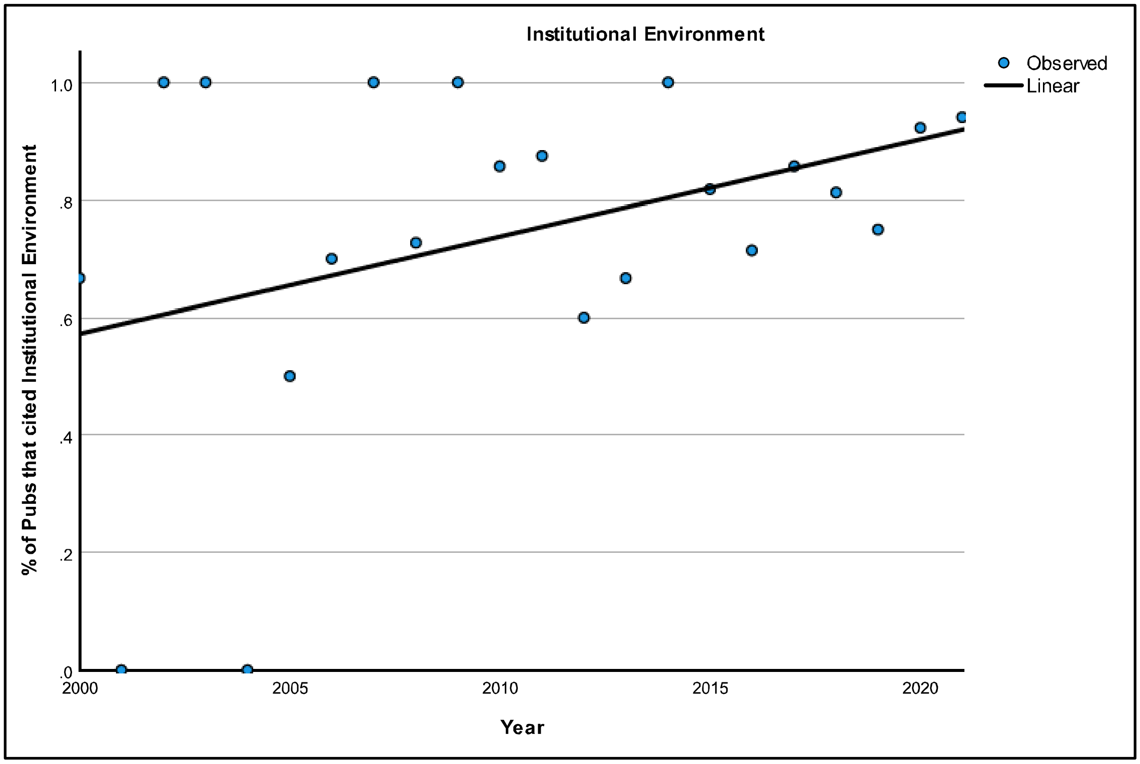

© 2024 Campopiano von Klimo M et al. *JAMA Network Open.*

Cognitive Capacity	
F	P-value
1.522	.232

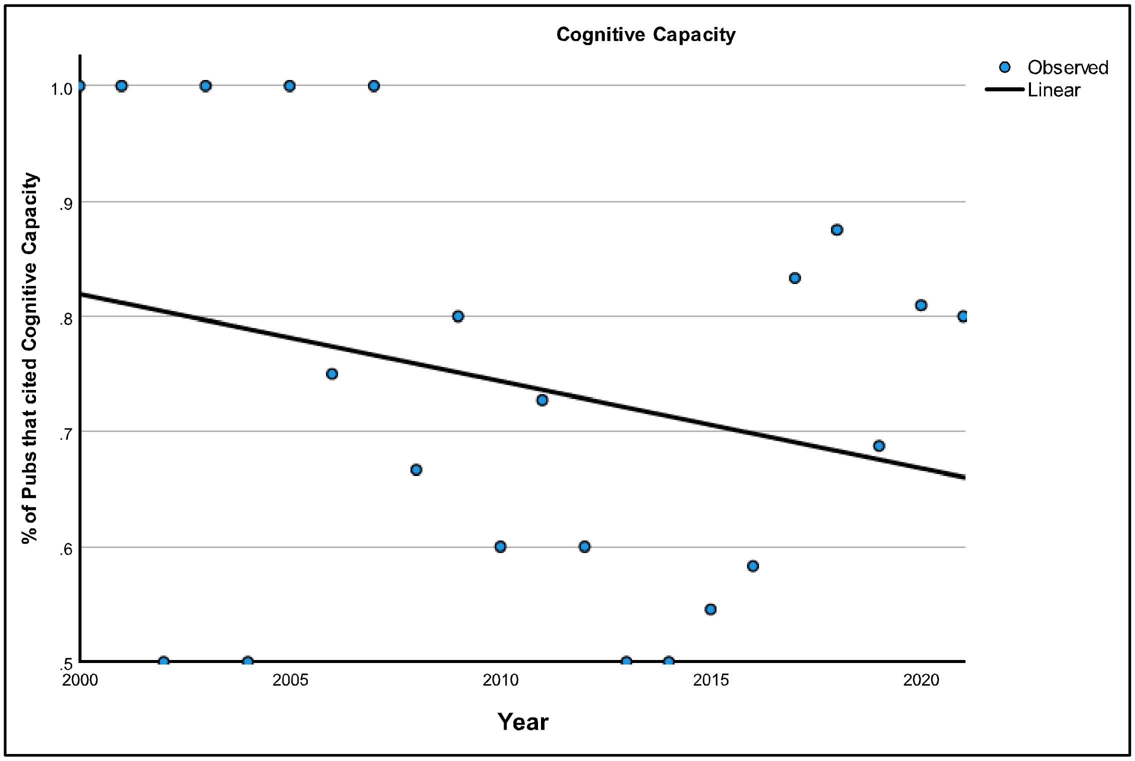

© 2024 Campopiano von Klimo M et al. *JAMA Network Open.*

Emotion	
F	P-value
3.799	.065

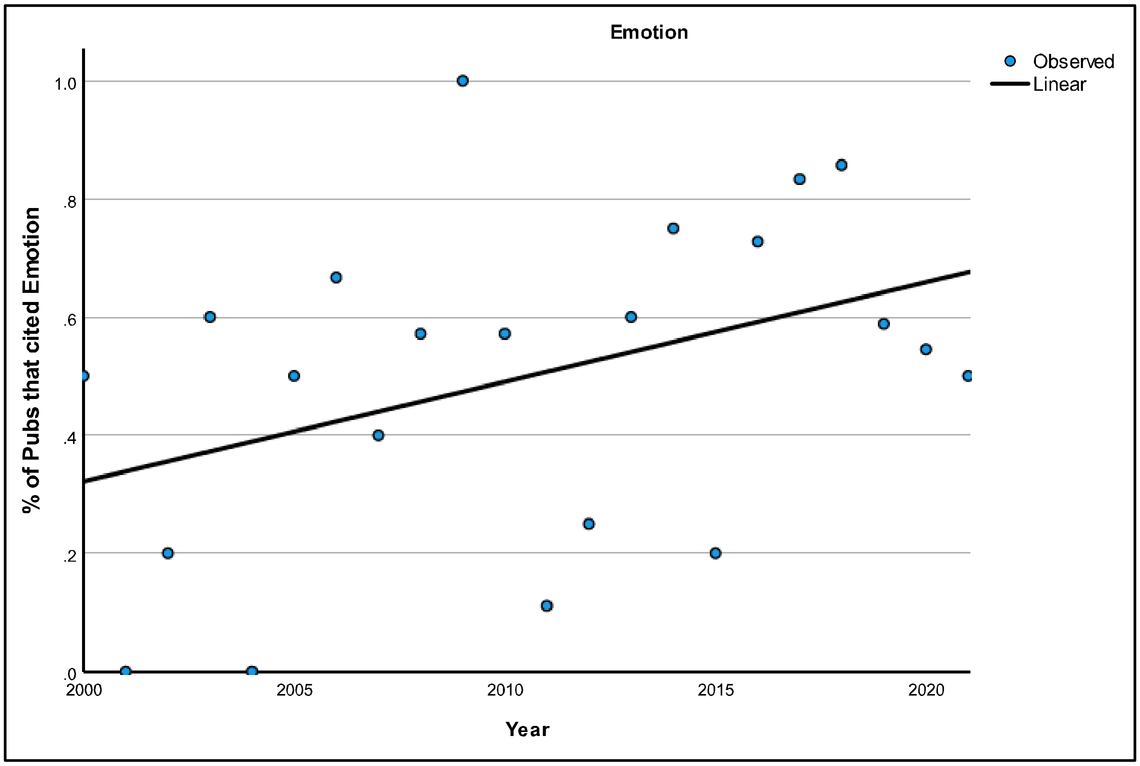

© 2024 Campopiano von Klimo M et al. *JAMA Network Open.*

Relationship	
F	P-value
4.543*	.046

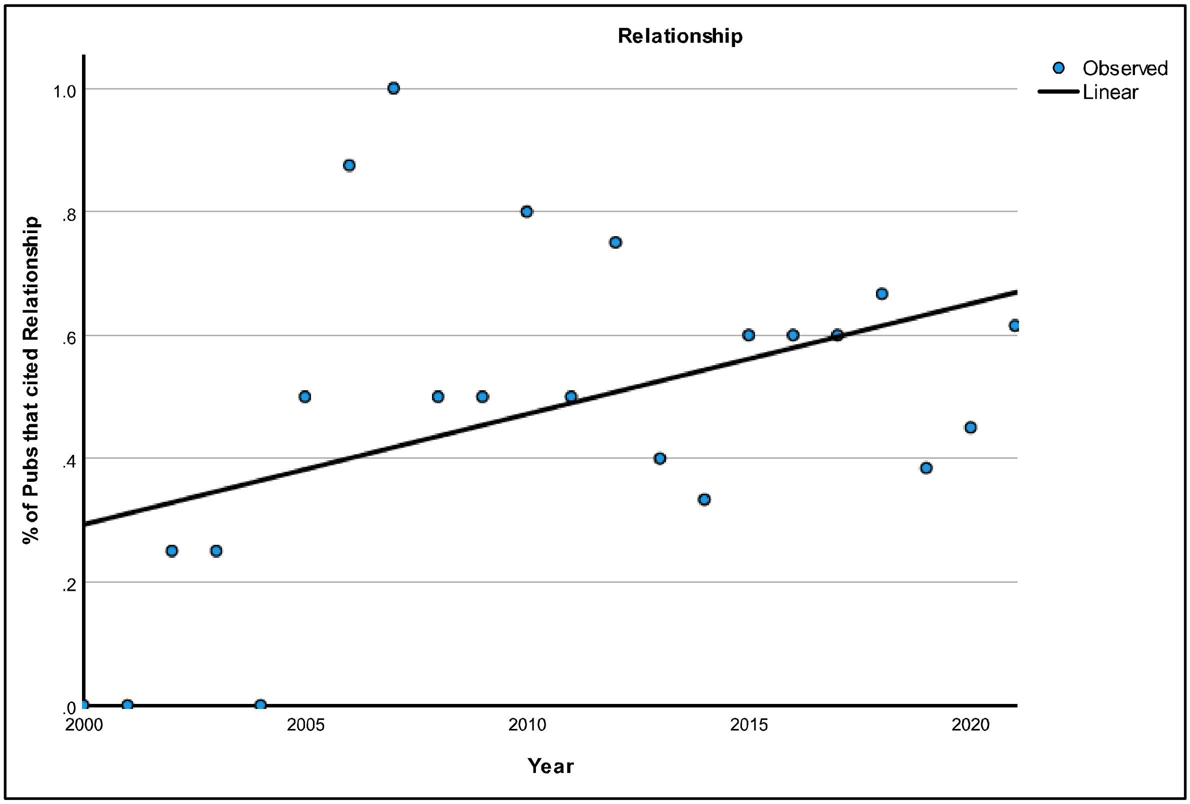

© 2024 Campopiano von Klimo M et al. *JAMA Network Open.*

eFigure 3. Distribution of Publications by Year

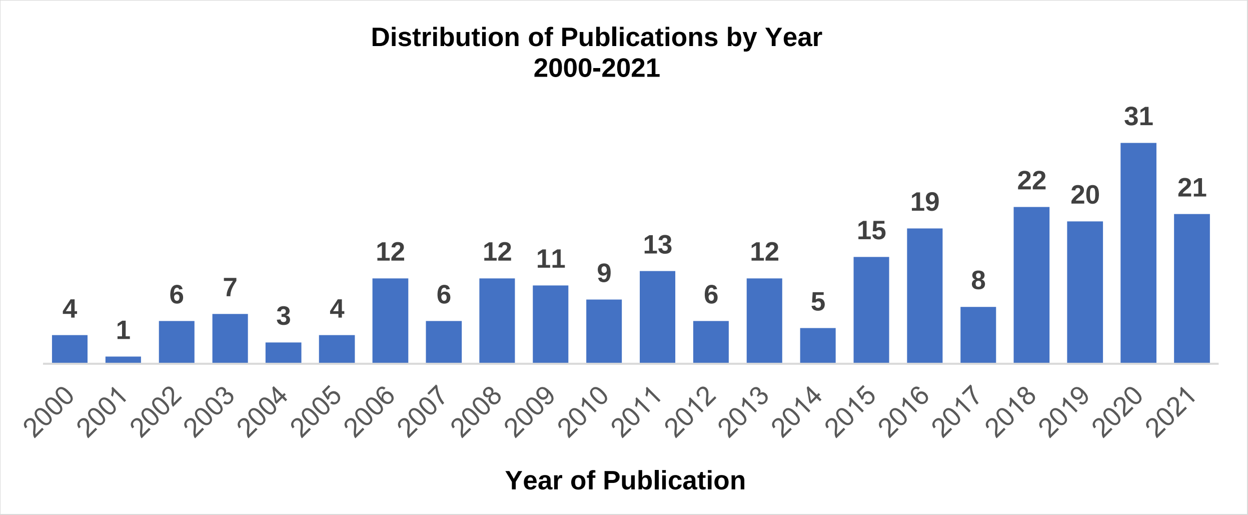

© 2024 Campopiano von Klimo M et al. *JAMA Network Open.*

eTable 1. Study Characteristics and Quality Data

Author	Year	Title	Reasons for reluctance cited	Factors impacting study quality (e.g., validity or precision of results)[a]	
				Theoretical framework[b]	Survey development best practices[c]
**Study type: report of survey-based research (170 articles)**					
**Aalto et al[1]**	2006	Do primary care physicians' own AUDIT scores predict their use of brief alcohol intervention? A cross-sectional survey	Knowledge<br>Skills	NA	NA
**Aalto et al[2]**	2001	Primary health care nurses' and physicians' attitudes, knowledge and beliefs regarding brief intervention for heavy drinkers	Knowledge<br>Skills<br>Reinforcement<br>Institutional environment	NA	NA
**Abed & Neira-Munoz[3]**	1990	A survey of general practitioners' opinion and attitude to drug addicts and addiction	Knowledge<br>Skills<br>Expectation of benefit<br>Reinforcement<br>Cognitive capacity<br>Institutional environment<br>Emotion	NA	Target audience involved in survey development<br><br>Piloted survey
**Adams et al[4]**	1997	Incentives for general practitioners to provide brief interventions for alcohol problems	Knowledge<br>Skills<br>Professional role/identity<br>Expectation of benefit<br>Reinforcement<br>Cognitive capacity<br>Institutional environment<br>Relationship	NA	Target audience involved in survey development<br><br>Piloted survey
**An et al[5]**	2004	Treatment of tobacco use as a chronic medical condition: primary care physicians' self-	Knowledge<br>Skills<br>Expectation of benefit	NA	NA

© 2024 Campopiano von Klimo M et al. *JAMA Network Open.*

Author	Year	Title	Reasons for reluctance cited	Factors impacting study quality (e.g., validity or precision of results)[a]	
				Theoretical framework[b]	Survey development best practices[c]
		reported practice patterns			
**Andrilla et al[6]**	2017	Barriers rural physicians face prescribing buprenorphine for opioid use disorder	Knowledge<br>Skills<br>Reinforcement<br>Cognitive capacity<br>Institutional environment<br>Social influences<br>Emotion	NA	NA
**Arfken et al[7]**	2010	Expanding treatment capacity for opioid dependence with office-based treatment with buprenorphine: national surveys of physicians	Skills<br>Expectation of benefit<br>Institutional environment<br>Emotion	NA	NA
**Bander et al[8]**	1987	Survey of attitudes among three specialties in a teaching hospital toward alcoholics	Knowledge<br>Professional role/identity<br>Expectation of benefit<br>Emotion	NA	NA
**Beletsky et al[9]**	2007	Physicians' knowledge of and willingness to prescribe naloxone to reverse accidental opiate overdose: challenges and opportunities	Skills<br>Expectation of benefits<br>Social influences<br>Emotion	Theory of Planned Behavior	NA
**Bell et al[10]**	1990	How willing are general practitioners to manage narcotic misuse?	Knowledge<br>Skills<br>Emotion<br>Other	NA	NA
**Bernstein et al[11]**	2007	An evidence-based alcohol screening, brief	Cognitive capacity	NA	NA

© 2024 Campopiano von Klimo M et al. *JAMA Network Open.*

Author	Year	Title	Reasons for reluctance cited	Factors impacting study quality (e.g., validity or precision of results)[a]	
				Theoretical framework[b]	Survey development best practices[c]
		intervention and referral to treatment (SBIRT) curriculum for emergency department (ED) providers improves skills and utilization	Institutional environment<br>Social influences<br>Relationship		
**Besson et al[12]**	2014	Opioid maintenance therapy in Switzerland: an overview of the Swiss IMPROVE study	Knowledge<br>Reinforcement<br>Institutional environment<br>Social influences<br>Emotion	NA	NA
**Bradley et al[13]**	1995	Primary and secondary prevention of alcohol problems: U.S. internist attitudes and practices	Expectation of benefit<br>Institutional environment<br>Relationship	NA	NA
**Broderick et al[14]**	2015	Emergency physician utilization of alcohol/substance screening, brief advice and discharge: a 10-year comparison	Expectation of benefit<br>Relationship	NA	Target audience involved in survey development<br><br>Piloted survey
**Calcaterra et al[15]**	2022	The impact of access to addiction specialist on attitudes, beliefs and hospital-based opioid use disorder related care: a survey of hospitalist physicians	Professional role/identity<br>Institutional environment<br>Social influences<br>Emotion	NA	Target audience involved in survey development

© 2024 Campopiano von Klimo M et al. *JAMA Network Open.*

Author	Year	Title	Reasons for reluctance cited	Factors impacting study quality (e.g., validity or precision of results)[a]	
				Theoretical framework[b]	Survey development best practices[c]
**Chan et al[16]**	2021	Knowledge, attitudes, practice, and barriers of physicians to provide tobacco dependence treatment: a cluster analysis	Skills<br>Professional role/identity<br>Expectation of benefit<br>Cognitive capacity	NA	NA
**Cilluffo et al[17]**	2020	Barriers and incentives for Italian paediatricians to become smoking cessation promoters: a GARD-Italy Demonstration Project	Expectation of benefit<br>Cognitive capacity<br>Relationship	NA	Target audience involved in survey development
**Collins et al[18]**	2018	An online survey of Irish general practitioner experience of and attitude toward managing problem alcohol use	Knowledge<br>Skills<br>Expectation of benefit	NA	NA
**Cotter et al[19]**	2020	Practice habits, knowledge, and attitudes of hepatologists to alcohol use disorder medication: sobering gaps and opportunities	Knowledge<br>Skills<br>Professional role/identity<br>Institutional environment	NA	Target audience involved in survey development
**Cunningham et al[20]**	2007	Barriers to obtaining waivers to prescribe buprenorphine for opioid addiction treatment	Knowledge<br>Reinforcement<br>Institutional environment<br>Emotion	NA	NA

© 2024 Campopiano von Klimo M et al. *JAMA Network Open.*

Author	Year	Title	Reasons for reluctance cited	Factors impacting study quality (e.g., validity or precision of results)[a]	
				Theoretical framework[b]	Survey development best practices[c]
		among HIV physicians			
**Cunningham et al[21]**	2010	National survey of emergency department alcohol screening and intervention practices	Knowledge<br>Skills<br>Expectation of benefit<br>Reinforcement<br>Cognitive capacity<br>Institutional environment	NA	Piloted survey
**Danielsson et al[22]**	1999	Reasons why trauma surgeons fail to screen for alcohol problems	Professional role/identity<br>Expectation of benefit<br>Cognitive capacity<br>Institutional environment<br>Social influences	NA	Piloted survey
**Day et al[23]**	2018	Oncologist provision of smoking cessation support: a national survey of Australian medical and radiation oncologists	Knowledge<br>Skills<br>Expectation of benefit<br>Cognitive capacity<br>Institutional environment<br>Emotion<br>Relationship<br>Patient refusal	NA	Piloted survey
**Deehan et al[24]**	1997	The general practitioner, the drug misuser, and the alcohol misuser: major differences in general practitioner activity, therapeutic commitment, and 'shared care' proposals	Skills<br>Reinforcement<br>Cognitive capacity<br>Institutional environment<br>Social influences<br>Emotion	NA	NA

© 2024 Campopiano von Klimo M et al. *JAMA Network Open.*

Author	Year	Title	Reasons for reluctance cited	Factors impacting study quality (e.g., validity or precision of results)[a]	
				Theoretical framework[b]	Survey development best practices[c]
**DeFlavio et al[25]**	2015	Analysis of barriers to adoption of buprenorphine maintenance therapy by family physicians	Knowledge<br>Skills<br>Professional role/identity<br>Expectation of benefit<br>Reinforcement<br>Cognitive capacity<br>Institutional environment<br>Emotion	NA	Piloted survey
**Demmert et al[26]**	2011	Attitudes towards brief interventions to reduce smoking and problem drinking behaviour in gynaecological practice	Knowledge<br>Skills<br>Expectation of benefit<br>Cognitive capacity<br>Relationship	NA	NA
**Donovan[27]**	1991	Factors predisposing, enabling and reinforcing routine screening of patients for preventing fetal alcohol syndrome: a survey of New Jersey physicians	Knowledge<br>Skills<br>Cognitive capacity<br>Relationship	PRECEDE (predisposing, reinforcing and enabling constructs in educational/economic diagnosis and evaluation)	NA
**Duszynski et al[28]**	1995	Reported practices, attitudes, and confidence levels of primary care physicians regarding patients who abuse alcohol and other drugs	Skills	NA	NA
**Ehrie et al[29]**	2020	Survey of addiction specialists' use	Knowledge<br>Expectation of benefit	NA	NA

© 2024 Campopiano von Klimo M et al. *JAMA Network Open.*

Author	Year	Title	Reasons for reluctance cited	Factors impacting study quality (e.g., validity or precision of results)[a]	
				Theoretical framework[b]	Survey development best practices[c]
		of medications to treat alcohol use disorder	Cognitive capacity<br>Cost to patient<br>Other		
**Elliott et al[30]**	2006	Diagnosis of foetal alcohol syndrome and alcohol use in pregnancy: a survey of paediatricians' knowledge, attitudes and practice	Knowledge<br>Relationship	NA	Target audience involved in survey development
**Elwy et al[31]**	2013	Physicians' attitudes toward unhealthy alcohol use and self-efficacy for screening and counseling as predictors of their counseling and primary care patients' drinking outcomes	Other	Theory of Planned Behavior	NA
**Ferguson et al[32]**	2003	Barriers to identification and treatment of hazardous drinkers as assessed by urban/rural primary care doctors	Cognitive capacity<br>Social influences<br>Emotion	NA	NA
**Fisher et al[33]**	1975	Physicians and alcoholics. The effect of medical training on attitudes toward alcoholics	Emotion	NA	NA
**Fisher et al[34]**	1975	Physicians and alcoholics. Factors affecting attitudes of family-practice	Emotion	Locus of Control	NA

© 2024 Campopiano von Klimo M et al. *JAMA Network Open.*

Author	Year	Title	Reasons for reluctance cited	Factors impacting study quality (e.g., validity or precision of results)[a]	
				Theoretical framework[b]	Survey development best practices[c]
		residents toward alcoholics			
**Fortmann et al[35]**	1985	Attitudes and practices of physicians regarding hypertension and smoking: the Stanford Five City project	Knowledge<br>Expectation of benefit<br>Emotion	NA	Target audience involved in survey development<br><br>Piloted Survey
**Foti et al[36]**	2021	Primary care physicians' preparedness to treat opioid use disorder in the United States: A cross-sectional survey	Skills<br>Cognitive capacity<br>Institutional environment	NA	Target audience involved in survey development<br><br>Piloted Survey
**Franz et al[37]**	2021	Physician blame and vulnerability: novel predictors of physician willingness to work with patients who misuse opioids	Reinforcement<br>Social influences<br>Emotion	NA	Target audience involved in survey development<br><br>Piloted Survey
**Franz et al[38]**	2021	Rural-urban differences in physician bias toward patients with opioid use disorder	Cognitive capacity<br>Institutional environment<br>Social influences<br>Emotion	NA	NA
**Gano et al[39]**	2018	Opioid overdose prevention in family medicine clerkships: a CERA study	Skills<br>Cognitive capacity<br>Institutional environment<br>Social influences<br>Other	NA	Target audience involved in survey development
**Gassman[40]**	2003	Medical specialization, profession, and mediating beliefs that	Knowledge<br>Skills<br>Professional role/identity	NA	NA

© 2024 Campopiano von Klimo M et al. *JAMA Network Open.*

Author	Year	Title	Reasons for reluctance cited	Factors impacting study quality (e.g., validity or precision of results)[a]	
				Theoretical framework[b]	Survey development best practices[c]
		predict stated likelihood of alcohol screening and brief intervention: targeting educational interventions			
**Geirsson et al[41]**	2005	Attitudes of Swedish general practitioners and nurses to working with lifestyle change, with special reference to alcohol consumption	Knowledge<br>Skills<br>Expectation of benefit<br>Cognitive capacity<br>Institutional environment<br>Social influences	NA	NA
**George & Martin[42]**	1992	GP's attitudes towards drug users	Institutional environment<br>Social influences<br>Emotion	NA	NA
**Glanz[43]**	1986	Findings of a national survey of the role of general practitioners in the treatment of opiate misuse: views on treatment	Knowledge<br>Skills<br>Professional role/identity<br>Institutional environment<br>Emotion	NA	NA
**Gokirmak et al[44]**	2010	The attitude toward tobacco dependence and barriers to discussing smoking cessation: a survey among Turkish general practitioners	Knowledge<br>Skills<br>Expectation of benefit<br>Reinforcement<br>Cognitive capacity<br>Emotion	NA	NA
**Gordon et al[45]**	2008	Outcomes of DATA 2000 certification trainings for the	Professional role/identity<br>Cognitive capacity	NA	NA

© 2024 Campopiano von Klimo M et al. *JAMA Network Open.*

Author	Year	Title	Reasons for reluctance cited	Factors impacting study quality (e.g., validity or precision of results)[a]	
				Theoretical framework[b]	Survey development best practices[c]
		provision of buprenorphine treatment in the Veterans Health Administration	Institutional environment<br>No demand		
**Graham et al[46]**	2000	Emergency physician attitudes concerning intervention for alcohol abuse/dependence delivered in the emergency department: a brief report	Skills<br>Professional role/identity<br>Expectation of benefit<br>Reinforcement<br>Cognitive capacity<br>Institutional environment	NA	Piloted Survey
**Gunderson et al[47]**	2006	Evaluation of a combined online and in person training in the use of buprenorphine	Knowledge<br>Skills<br>Reinforcement<br>Cognitive capacity<br>Institutional environment<br>Emotion	NA	NA
**Hammond et al[48]**	2021	Provider and practice characteristics and perceived barriers associated with different levels of adolescent SBIRT implementation among a national sample of US pediatricians	Knowledge<br>Skills<br>Cognitive capacity<br>Institutional environment<br>Relationship	NA	NA
**Harris & Yu[49]**	2016	Attitudes, perceptions and practice of alcohol and drug screening, brief intervention and referral to treatment: a	Expectation of benefit	NA	NA

© 2024 Campopiano von Klimo M et al. *JAMA Network Open.*

Author	Year	Title	Reasons for reluctance cited	Factors impacting study quality (e.g., validity or precision of results)[a]	
				Theoretical framework[b]	Survey development best practices[c]
		case study of New York State primary care physicians and non-physician providers			
**Hayes et al[50]**	2018	Suboxone and me: an OSCE-based assessment of medical residents' knowledge of medical treatment of opioid addiction [Abstract]	Knowledge<br>Skills	NA	NA
**Hernandez-Meier et al[51]**	2019	Emergency medicine provider attitudes toward and experiences with prescribing buprenorphine in the ED of a large academic metropolitan hospital	Knowledge<br>Skills<br>Institutional environment<br>Social influences	NA	NA
**Hernandez-Meier et al[52]**	2020	Emergency provider practices and attitudes around naloxone-prescribing in an academic emergency department	Knowledge<br>Skills<br>Cognitive capacity<br>Institutional environment<br>Emotion	NA	NA
**Hoffmann et al[53]**	2019	Are German family practitioners and psychiatrists sufficiently trained to diagnose and treat patients with alcohol problems?	Knowledge<br>Skills		Target audience involved in survey development

© 2024 Campopiano von Klimo M et al. *JAMA Network Open.*

Author	Year	Title	Reasons for reluctance cited	Factors impacting study quality (e.g., validity or precision of results)[a]	
				Theoretical framework[b]	Survey development best practices[c]
**Holmqvist et al[54]**	2008	Towards increased alcohol intervention activity in Swedish occupational health services	Knowledge<br>Skills<br>Cognitive capacity<br>Relationship		Target audience involved in survey development
**Huhn & Dunn[55]**	2017	Why aren't physicians prescribing more buprenorphine?	Knowledge<br>Skills<br>Expectation of benefit<br>Reinforcement<br>Cognitive capacity<br>Institutional environment<br>Social influences<br>Emotion	NA	NA
**James et al[56]**	2019	Interest in prescribing buprenorphine among resident and attending physicians at an urban teaching clinic	Professional role/identity<br>Expectation of benefit	NA	NA
**Johansson et al[57]**	2002	Early intervention for problem drinkers: readiness to participate among general practitioners and nurses in Swedish primary health care	Knowledge<br>Skills<br>Expectation of benefit<br>Cognitive capacity<br>Institutional environment	NA	NA
**Johnson et al[58]**	2020	211 Impact of X-waiver training on resident barriers and biases surrounding buprenorphine treatment for	Knowledge<br>Skills<br>Expectation of benefit<br>Institutional environment<br>Social influences	NA	NA

© 2024 Campopiano von Klimo M et al. *JAMA Network Open.*

Author	Year	Title	Reasons for reluctance cited	Factors impacting study quality (e.g., validity or precision of results)[a]	
				Theoretical framework[b]	Survey development best practices[c]
		opiate use disorder	Emotion		
**Johnson et al[59]**	2005	Physician beliefs about substance misuse and its treatment: findings from a U.S. survey of primary care practitioners	Knowledge<br>Skills<br>Expectation of benefit<br>Reinforcement<br>Cognitive capacity<br>Emotion<br>Relationship		Target audience involved in survey development<br><br>Piloted survey
**Jones & McCance-Katz[60]**	2019	Characteristics and prescribing practices of clinicians recently waivered to prescribe buprenorphine for the treatment of opioid use disorder	Cognitive capacity<br>Institutional environment<br>Social influences<br>Emotion<br>No demand	NA	NA
**Kaner et al[61]**	2001	Patient and practitioner characteristics predict brief alcohol intervention in primary care	Skills<br>Professional role/identity<br>Cognitive capacity	NA	NA
**Kaner et al[62]**	1999	Intervention for excessive alcohol consumption in primary health care: attitudes and practices of English general practitioners	Skills<br>Professional role/identity<br>Expectation of benefit<br>Reinforcement<br>Cognitive capacity<br>Institutional environment<br>Social influences<br>Relationship		Target audience involved in survey development<br><br>Piloted survey
**Karam-Hage et al[63]**	2001	Modifying residents' professional attitudes about substance	Knowledge<br>Emotion	NA	NA

© 2024 Campopiano von Klimo M et al. *JAMA Network Open.*

Author	Year	Title	Reasons for reluctance cited	Factors impacting study quality (e.g., validity or precision of results)[a]	
				Theoretical framework[b]	Survey development best practices[c]
		abuse treatment and training			
**Keller et al[64]**	2012	Practices, perceptions, and concerns of primary care physicians about opioid dependence associated with the treatment of chronic pain	Knowledge<br>Skills<br>Emotion	NA	NA
**Kennedy et al[65]**	2018	Impact of attending a mutual support group meeting on resident trainee attitudes toward patients with substance use disorder [Abstract]	Knowledge<br>Skills<br>Emotion	NA	NA
**Kennedy-Hendricks et al[66]**	2016	Primary care physicians' perspectives on the prescription opioid epidemic	Emotion	NA	NA
**Kermack et al[67]**	2017	Buprenorphine prescribing practice trends and attitudes among New York providers	Reinforcement<br>Cognitive capacity<br>Institutional environment<br>Social influences		Target audience involved in survey development<br><br>Piloted survey
**Kershaw et al[68]**	2018	Educational intervention to improve communication with patients who have opioid use disorder.	Institutional environment<br>Social influences<br>Emotion	NA	NA
**Kestler et al[69]**	2021	A cross-sectional survey on buprenorphine-naloxone	Knowledge<br>Skills<br>Cognitive capacity		Target audience involved in survey development

© 2024 Campopiano von Klimo M et al. *JAMA Network Open.*

Author	Year	Title	Reasons for reluctance cited	Factors impacting study quality (e.g., validity or precision of results)[a]	
				Theoretical framework[b]	Survey development best practices[c]
		practice and attitudes in 22 Canadian emergency physician groups: a cross-sectional survey	Institutional environment		Piloted survey
**Keto et al[70]**	2015	Physicians discuss the risks of smoking with their patients, but seldom offer practical cessation support	Expectation of benefit	NA	NA
**Kiernon et al[71]**	2015	Reducing consumption versus maintaining abstinence: market access challenges facing a novel treatment pathway for alcohol addiction in the EU5	Knowledge<br>Expectation of benefit	NA	NA
**Kirane et al[72]**	2018	Addressing the opioid crisis: assessment of clinicians in a large healthcare system	Knowledge<br>Skills<br>Professional role/identity<br>Reinforcement<br>Institutional environment<br>Emotion<br>No demand		Target audience involved in survey development<br><br>Piloted survey
**Kissin et al[73]**	2006	Experiences of a national sample of qualified addiction specialists who have and have not prescribed buprenorphine for opioid dependence	Institutional environment<br>Emotion	NA	NA

© 2024 Campopiano von Klimo M et al. *JAMA Network Open.*

Author	Year	Title	Reasons for reluctance cited	Factors impacting study quality (e.g., validity or precision of results)[a]	
				Theoretical framework[b]	Survey development best practices[c]
**Ko et al[74]**	2020	Obstetrician–gynecologists' practice patterns related to opioid use during pregnancy and postpartum - United States, 2017	Cognitive capacity<br>Institutional environment<br>Social influences<br>Emotion<br>Patient refusal	NA	NA
**Kohan et al[75]**	2021	Presence of opioid safety initiatives, prescribing patterns for opioid and naloxone, and perceived barriers to prescribing naloxone: cross-sectional survey results based on practice type, scope, and location	Cognitive capacity<br>Institutional environment<br>Relationship<br>Cost to patient	NA	NA
**Konfino et al[76]**	2011	Knowledge, attitudes and practices regarding smoking and cessation advice: a survey of physicians in Buenos Aires [Abstract]	Knowledge<br>Skills<br>Cognitive capacity	NA	NA
**Koopman et al[77]**	2008	Addressing alcohol problems in primary care settings: a study of general medical practitioners in Cape Town, South Africa	Knowledge<br>Skills<br>Professional role/identity<br>Expectation of benefit<br>Reinforcement<br>Cognitive capacity<br>Institutional environment<br>Relationship	NA	NA

© 2024 Campopiano von Klimo M et al. *JAMA Network Open.*

Author	Year	Title	Reasons for reluctance cited	Factors impacting study quality (e.g., validity or precision of results)[a]	
				Theoretical framework[b]	Survey development best practices[c]
**Kouyoumdjian et al[78]**	2018	Physician prescribing of opioid agonist treatments in provincial correctional facilities in Ontario, Canada: a survey	Knowledge<br>Professional role/identity<br>Expectation of benefit<br>Cognitive capacity<br>Institutional environment<br>Social influences<br>Emotion<br>Relationship	NA	Target audience involved in survey development
**Kunins et al[79]**	2009	HIV provider endorsement of primary care buprenorphine treatment: A vignette study	Knowledge<br>Skills<br>Emotion	NA	NA
**Kunze & Haidinger[80]**	1997	Alcoholism and nutrition results of a survey among general practitioners	Expectation of benefit<br>Institutional environment<br>Social influences	NA	NA
**Lacroix et al[81]**	2018	Emergency physicians' attitudes and perceived barriers to the implementation of take-home naloxone programs in Canadian emergency departments	Knowledge<br>Skills<br>Cognitive capacity<br>Institutional environment		Target audience involved in survey development<br><br>Piloted survey
**Langton et al[82]**	2000	Methadone maintenance in general practice: impact on staff attitudes	Cognitive capacity	NA	NA
**Latorre et al[83]**	2007	Primary care doctors' perception of treatment	Knowledge<br>Skills<br>Professional role/identity	NA	Target audience involved in

© 2024 Campopiano von Klimo M et al. *JAMA Network Open.*

Author	Year	Title	Reasons for reluctance cited	Factors impacting study quality (e.g., validity or precision of results)[a]	
				Theoretical framework[b]	Survey development best practices[c]
		demand and need for training in drug addiction issues	Cognitive capacity<br>Institutional environment<br>Social influences		survey development
**Le et al[84]**	2015	Primary care residents lack comfort and experience with alcohol screening and brief intervention: a multi-site survey	Knowledge<br>Skills<br>Expectation of benefit<br>Reinforcement<br>Cognitive capacity<br>Relationship	NA	Target audience involved in survey development<br><br>Piloted survey
**Levy et al[85]**	2020	Adolescent SBIRT practices among pediatricians in Massachusetts	Knowledge<br>Skills<br>Cognitive capacity<br>Institutional environment<br>Social influences<br>Relationship	NA	NA
**Linn & Yager[86]**	1989	Factors associated with physician recognition and treatment of alcoholism	Professional role/identity<br>Expectation of benefit<br>Institutional environment	NA	NA
**Logan et al[87]**	2020	85 Physician-perceived barriers to treating opiate use disorder in the emergency department	Institutional environment<br>Social influences	NA	NA
**Loheswaran et al[88]**	2015	Screening and treatment for alcohol, tobacco and opioid use disorders: a survey of family physicians across Ontario	Knowledge<br>Expectation of benefit<br>Reinforcement	NA	Target audience involved in survey development<br><br>Piloted survey
**Lowenstein et al[89]**	2019	Barriers and facilitators for	Knowledge<br>Skills	NA	NA

© 2024 Campopiano von Klimo M et al. *JAMA Network Open.*

Author	Year	Title	Reasons for reluctance cited	Factors impacting study quality (e.g., validity or precision of results)[a]	
				Theoretical framework[b]	Survey development best practices[c]
		emergency department initiation of buprenorphine: a physician survey	Reinforcement<br>Cognitive capacity<br>Institutional environment<br>Social influences<br>Emotion<br>No demand		
**Macalino et al[90]**	2009	A national physician survey on prescribing syringes as an HIV prevention measure	Knowledge<br>Expectation of benefit<br>Institutional environment<br>Social influences<br>Emotion	Theory of Planned Behavior	NA
**Marcell et al[91]**	2002	Physicians' attitudes and beliefs concerning alcohol abuse prevention in adolescents	Knowledge<br>Skills	NA	NA
**Mark et al[92]**	2003	Understanding US addiction physicians' low rate of naltrexone prescription	Knowledge<br>Expectation of benefit<br>Institutional environment<br>Emotion<br>No demand<br>Cost to patient	NA	Target audience involved in survey development
**Mark et al[93]**	2003	Physicians' opinions about medications to treat alcoholism	Knowledge<br>Expectation of benefit<br>Institutional environment<br>Social influences<br>Relationship<br>Patient refusal<br>Other	NA	Piloted survey
**Martinez et al[94]**	2016	Pharmacotherapy for alcohol use disorders: physicians'	Knowledge<br>Skills<br>Expectation of benefit	NA	NA

© 2024 Campopiano von Klimo M et al. *JAMA Network Open.*

Author	Year	Title	Reasons for reluctance cited	Factors impacting study quality (e.g., validity or precision of results)[a]	
				Theoretical framework[b]	Survey development best practices[c]
		perceptions and practices	Institutional environment		
**Martino et al[95]**	2019	Physician and pharmacist: attitudes, facilitators, and barriers to prescribing naloxone for home rescue	Knowledge<br>Skills<br>Professional role/identity<br>Reinforcement<br>Cognitive capacity<br>Institutional environment<br>Relationship<br>Patient refusal<br>Cost to patient	NA	NA
**Matheson et al[96]**	2010	Management of drug misuse: an 8-year follow-up survey of Scottish GPs	Institutional environment<br>Emotion	NA	Target audience involved in survey development<br><br>Piloted survey
**May et al[97]**	2002	Attitudes of anesthesiologist s about addiction and its treatment: a survey of Illinois and Wisconsin members of the American society of anesthesiologist s	Knowledge<br>Skills<br>Expectation of benefit<br>Emotion	NA	NA
**Mayet et al[98]**	2011	Impact of training for healthcare professionals on how to manage an opioid overdose with naloxone: Effective, but dissemination is challenging	Knowledge<br>Skills<br>Cognitive capacity<br>Institutional environment<br>Social influences	NA	NA
**McGillion et al[99]**	2000	GPs' attitudes towards the	Knowledge<br>Expectation of benefit	NA	NA

© 2024 Campopiano von Klimo M et al. *JAMA Network Open.*

Author	Year	Title	Reasons for reluctance cited	Factors impacting study quality (e.g., validity or precision of results)[a]	
				Theoretical framework[b]	Survey development best practices[c]
		treatment of drug misusers	Reinforcement<br>Emotion		
**Meijer & Chavannes[100]**	2021	Lacking willpower? A latent class analysis of healthcare providers' perceptions of smokers' responsibility for smoking	Knowledge<br>Skills<br>Professional role/identity<br>Reinforcement<br>Cognitive capacity<br>Institutional environment<br>Social influences<br>Emotion<br>Relationship	NA	NA
**Meijer et al[101]**	2018	What keeps healthcare professionals from advising their patients who smoke to quit? A large-scale cross-sectional study	Knowledge<br>Skills<br>Professional role/identity<br>Reinforcement	NA	NA
**Meijer et al[102]**	2019	Facilitating smoking cessation in patients who smoke: A large-scale cross-sectional comparison of fourteen groups of healthcare providers	Knowledge<br>Skills<br>Professional role/identity<br>Expectation of benefit<br>Cognitive capacity<br>Relationship	NA	NA
**Mellinger et al[103]**	2020	Provider attitudes and practices for alcohol screening, treatment and education in patients with liver disease	Knowledge<br>Skills<br>Professional role/identity<br>Expectation of benefit<br>Cognitive capacity<br>Relationship	NA	NA
**Messina et al[104]**	2020	Fetal alcohol spectrum disorders	Knowledge<br>Skills	NA	NA

© 2024 Campopiano von Klimo M et al. *JAMA Network Open.*

Author	Year	Title	Reasons for reluctance cited	Factors impacting study quality (e.g., validity or precision of results)[a]	
				Theoretical framework[b]	Survey development best practices[c]
		awareness in health professionals: implications for psychiatry			
**Midmer et al[105]**	2011	Efficacy of a physicians' pocket guide about prenatal substance use: a randomized trial	Knowledge<br>Skills	NA	NA
**Miller et al[106]**	2016	How Australian general practitioners engage in discussions about alcohol with their patients: a cross-sectional study	Cognitive capacity<br>Relationship	NA	Target audience involved in survey development<br><br>Piloted survey
**Miller & Frances[107]**	1986	Psychiatrists and the treatment of addictions: Perceptions and practices	Institutional environment<br>Social influences<br>Emotion	NA	NA
**Miquel et al[108]**	2018	Barriers to implement screening for alcohol consumption in Spanish hypertensive patients	Knowledge<br>Skills<br>Professional role/identity<br>Expectation of benefit<br>Reinforcement<br>Cognitive capacity<br>Social influences<br>Emotion<br>Relationship	NA	Target audience involved in survey development<br><br>Piloted survey
**Mitchell et al[109]**	2016	Education for the mind and the heart? Changing residents' attitudes about addressing	Knowledge<br>Skills<br>Professional role/identity<br>Reinforcement	NA	NA

© 2024 Campopiano von Klimo M et al. *JAMA Network Open.*

Author	Year	Title	Reasons for reluctance cited	Factors impacting study quality (e.g., validity or precision of results)[a]	
				Theoretical framework[b]	Survey development best practices[c]
		unhealthy alcohol use	Institutional environment<br>Social influences		
**Moatti et al[110]**	1998	French general practitioners' attitudes toward maintenance drug abuse treatment with buprenorphine	Knowledge<br>Skills<br>Expectation of benefit<br>Institutional environment<br>Social influences	NA	NA
**Mony & Jayakumar[111]**	2011	Preparedness for tobacco control among postgraduate residents of a medical college in Bangalore	Knowledge<br>Skills<br>Expectation of benefit<br>Social influences	Transtheoretical Model of Behavior Change	Piloted survey
**Myles et al[112]**	2020	Emergency physician knowledge, attitudes, and barriers to emergency department-delivered buprenorphine	Knowledge<br>Skills<br>Expectation of benefit<br>Cognitive capacity<br>Institutional environment<br>Social influences<br>Patient refusal	NA	NA
**Netherland et al[113]**	2009	Factors affecting willingness to provide buprenorphine treatment	Knowledge<br>Skills<br>Professional role/identity<br>Reinforcement<br>Institutional environment<br>Social influences<br>Emotion	NA	NA
**Nygaard et al[114]**	2010	Use and barriers to use of screening and brief interventions for alcohol	Knowledge<br>Skills<br>Reinforcement<br>Social influences<br>Relationship	NA	NA

© 2024 Campopiano von Klimo M et al. *JAMA Network Open.*

Author	Year	Title	Reasons for reluctance cited	Factors impacting study quality (e.g., validity or precision of results)[a]	
				Theoretical framework[b]	Survey development best practices[c]
		problems among Norwegian general practitioners			
**Ordean et al[115]**	2020	Screening, brief intervention, and referral to treatment for prenatal alcohol use and cigarette smoking: a survey of academic and community health care providers	Knowledge<br>Skills<br>Expectation of benefit<br>Reinforcement<br>Cognitive capacity<br>Institutional environment<br>Relationship	NA	NA
**O'Rourke et al[116]**	2006	Alcohol-related problems: emergency physicians' current practice and attitudes	Knowledge<br>Skills<br>Professional role/identity<br>Expectation of benefit<br>Social influences<br>Emotion	NA	NA
**Peckham et al[117]**	2018	A survey of prescribers' attitudes, knowledge, comfort, and fear of consequences related to an opioid overdose education and naloxone distribution program	Knowledge<br>Skills<br>Cognitive capacity	NA	NA
**Pelet et al[118]**	2005	Difficulties associated with outpatient management of drug abusers by general practitioners. A cross-sectional	Knowledge<br>Skills<br>Expectation of benefit<br>Reinforcement<br>Cognitive capacity<br>Emotion	NA	Target audience involved in survey development

© 2024 Campopiano von Klimo M et al. *JAMA Network Open.*

Author	Year	Title	Reasons for reluctance cited	Factors impacting study quality (e.g., validity or precision of results)[a]	
				Theoretical framework[b]	Survey development best practices[c]
		survey of general practitioners with and without methadone patients in Switzerland	No demand		
**Peterson et al[119]**	2007	Harm minimization strategies: opinions of health professionals in rural and remote Australia	Skills<br>Expectation of benefit<br>Reinforcement<br>Cognitive capacity<br>Institutional environment<br>Social influences<br>Emotion<br>No demand	NA	NA
**Porath-Waller et al[120]**	2015	Perceptions among healthcare professionals of prescription drug misuse	Skills<br>Expectation of benefit<br>Institutional environment	NA	NA
**Price et al[121]**	2006	Obstetricians and gynecologists' perceptions and use of nicotine replacement therapy	Expectation of benefit<br>Institutional environment<br>Social influences<br>Emotion<br>Relationship	NA	NA
**Pulcinelli et al[122]**	1978	Professional knowledge on the subject of drug addiction. First results of a survey	Knowledge<br>Skills	NA	NA
**Punzal et al[123]**	2019	Current practices in naloxone prescribing upon hospital discharge	Skills<br>Expectation of benefit<br>Cognitive capacity<br>Emotion	NA	Target audience involved in survey development

© 2024 Campopiano von Klimo M et al. *JAMA Network Open.*

Author	Year	Title	Reasons for reluctance cited	Factors impacting study quality (e.g., validity or precision of results)[a]	
				Theoretical framework[b]	Survey development best practices[c]
					Piloted survey
**Pytell et al[124]**	2019	A pilot office-based opioid treatment clinic in an internal medicine resident continuity practice: provider, staff, and patient outcomes	Knowledge<br>Skills<br>Professional role/identity<br>Expectation of benefit<br>Institutional environment	NA	NA
**Ramos et al[125]**	2017	Adolescent substance use: assessing the knowledge, attitudes, and practices of a school-based health center workforce	Cognitive capacity<br>Institutional environment<br>Relationship	NA	Target audience involved in survey development
**Raupach et al[126]**	2011	Knowledge gaps about smoking cessation in hospitalized patients and their doctors	Skills<br>Professional role/identity<br>Expectation of benefit<br>Cognitive capacity	NA	NA
**Richmond & Mendelsohn[127]**	1998	Physicians' views of programs incorporating stages of change to reduce smoking and excessive alcohol consumption	Expectation of benefit<br>Reinforcement<br>Cognitive capacity<br>Institutional environment<br>No demand	NA	NA
**Rock & Silsby[128]**	1975	The attitudes of American physicians stationed with the United States Army, Europe, in regard to alcohol and drug abuse	Professional role/identity<br>Expectation of benefit<br>Institutional environment<br>Social influences	NA	NA

© 2024 Campopiano von Klimo M et al. *JAMA Network Open.*

Author	Year	Title	Reasons for reluctance cited	Factors impacting study quality (e.g., validity or precision of results)[a]	
				Theoretical framework[b]	Survey development best practices[c]
**Rowland et al[129]**	1988	Doctors and alcohol screening--the gap between attitudes and action	Knowledge<br>Professional role/identity<br>Cognitive capacity<br>Relationship	NA	NA
**Ruetsch & Schoenberger[130]**	2009	Factors that influence decisions among MBHO network physicians to use office-based opioid treatment or to increase the number of opioid-dependent patients they treat: results from the OBOT attitude and intention physician survey	Cognitive capacity<br>Institutional environment<br>Emotion	NA	NA
**Russell et al[131]**	2021	Attending a biopsychosocially focused buprenorphine training improves clinician attitudes		NA	NA
**Samuels et al[132]**	2016	Emergency department-based opioid harm reduction: moving physicians from willing to doing	Knowledge<br>Skills<br>Professional role/identity<br>Expectation of benefit<br>Reinforcement<br>Cognitive capacity<br>Institutional environment<br>Emotion	Theory of Planned Behavior	Piloted survey
**Satre et al[133]**	2012	Using needs assessment to	Knowledge<br>Skills	NA	NA

© 2024 Campopiano von Klimo M et al. *JAMA Network Open.*

Author	Year	Title	Reasons for reluctance cited	Factors impacting study quality (e.g., validity or precision of results)[a]	
				Theoretical framework[b]	Survey development best practices[c]
		develop curricula for screening, brief intervention, and referral to treatment (SBIRT) in academic and community health settings	Cognitive capacity<br>Institutional environment<br>Relationship		
**Savage & Ross[134]**	2022	Barriers and attitudes reported by Canadian emergency physicians regarding the initiation of buprenorphine/naloxone in the emergency department for patients with opioid use disorder	Knowledge<br>Skills<br>Cognitive capacity<br>Institutional environment<br>Social influences	NA	Target audience involved in survey development
**Schaeffer et al[135]**	2016	Emergency prescribers and rescue naloxone: Results of a health-system survey	Knowledge<br>Expectation of benefit	NA	NA
**Schulte et al[136]**	2013	Structural barriers in the context of opiate substitution treatment in Germany--a survey among physicians in primary care	Reinforcement<br>Institutional environment<br>Social influences	NA	NA
**Sell & Visconti[137]**	2020	Harm Reduction: Assessing the educational needs of family medicine	Knowledge<br>Skills<br>Institutional environment<br>Social influences	NA	NA

© 2024 Campopiano von Klimo M et al. *JAMA Network Open.*

Author	Year	Title	Reasons for reluctance cited	Factors impacting study quality (e.g., validity or precision of results)[a]	
				Theoretical framework[b]	Survey development best practices[c]
		residents in care of persons who inject drugs	Emotion Relationship		
**Sheffer et al[138]**	2009	Training health care providers in the treatment of tobacco use and dependence: pre- and post-training results	Skills Expectation of benefit Social influences	NA	NA
**Shuey et al[139]**	2021	Evaluation of resident physicians' knowledge of and attitudes towards prescribing buprenorphine for patients with opioid use disorder	Knowledge Professional role/identity Institutional environment	NA	NA
**Sinclair et al[140]**	2014	New York state emergency department providers' attitudes on naloxone distribution for treatment of opioid overdose: a preliminary study	Professional role/identity Expectation of benefit Emotion	NA	NA
**Spandorfer et al[141]**	1999	Primary care physicians' views on screening and management of alcohol abuse: inconsistencies with national guidelines	Expectation of benefit Cognitive capacity Social influences Relationship	NA	NA
**Stone et al[142]**	2021	The role of stigma in U.S. primary care physicians' treatment of	Social influences Emotion	NA	NA

© 2024 Campopiano von Klimo M et al. *JAMA Network Open.*

Author	Year	Title	Reasons for reluctance cited	Factors impacting study quality (e.g., validity or precision of results)[a]	
				Theoretical framework[b]	Survey development best practices[c]
		opioid use disorder			
**Strange[143]**	1971	The federal physician's attitude toward alcoholism: a sampling of naval medical officers' opinions	Knowledge<br>Expectation of benefit<br>Institutional environment<br>Emotion	NA	NA
**Sullivan et al[144]**	2006	Training HIV physicians to prescribe buprenorphine for opioid dependence	Skills<br>Expectation of benefit<br>Institutional environment<br>Emotion	NA	NA
**Taylor et al[145]**	2003	Would you consider prescribing syringes to injection drug users?	Professional role/identity<br>Expectation of benefit<br>Institutional environment<br>Social influences	NA	NA
**Tello et al[146]**	2019	A survey of attitudes around opioid use disorder and perceived barriers to providing buprenorphine maintenance treatment among outpatient primary care providers in an urban academic medical setting	Emotion	NA	NA
**Thomas et al[147]**	2003	Research to practice: adoption of naltrexone in alcoholism treatment	Knowledge<br>Expectation of benefit<br>Institutional environment<br>Social influences	Framework: Diffusion theory<br>Theory: Transtheoretical Model of Behavior Change	Target audience involved in survey development<br><br>Piloted survey

© 2024 Campopiano von Klimo M et al. *JAMA Network Open.*

Author	Year	Title	Reasons for reluctance cited	Factors impacting study quality (e.g., validity or precision of results)[a]	
				Theoretical framework[b]	Survey development best practices[c]
**Tiako & Mahmood[148]**	2020	Cardiac surgeons' practices, attitudes regarding addiction care and patients who use drugs	Reinforcement<br>Institutional environment<br>Emotion	NA	NA
**Twardella & Brenner[149]**	2005	Lack of training as a central barrier to the promotion of smoking cessation: a survey among general practitioners in Germany	Skills<br>Professional role/identity<br>Reinforcement<br>Cognitive capacity<br>Institutional environment	NA	NA
**Vader & Aufseesser[150]**	1993	Physicians and intravenous drug users: attitudes and opinions in the Canton of Vaud, Switzerland	Knowledge<br>Skills<br>Professional role/identity<br>Reinforcement<br>Social influences<br>Emotion<br>Relationship	NA	NA
**Van Boekel et al[151]**	2014	Healthcare professionals' regard towards working with patients with substance use disorders: comparison of primary care, general psychiatry and specialist addiction services	Knowledge<br>Reinforcement<br>Emotion	NA	NA
**Waal et al[152]**	2012	General practitioners' views on drug-assisted rehabilitation	Cognitive capacity<br>Institutional environment	NA	NA

© 2024 Campopiano von Klimo M et al. *JAMA Network Open.*

Author	Year	Title	Reasons for reluctance cited	Factors impacting study quality (e.g., validity or precision of results)[a]	
				Theoretical framework[b]	Survey development best practices[c]
**Wakeman et al[153]**	2013	Internal medicine residents' training in substance use disorders	Knowledge<br>Skills	NA	NA
**Wakeman et al[154]**	2013	Internal medicine residents training in substance use disorders: a survey of the quality of instruction and residents self-perceived preparedness to diagnose and treat addiction	Knowledge<br>Skills<br>Emotion	NA	NA
**Wakeman et al[155]**	2017	Institutional substance use disorder intervention improves general internist preparedness, attitudes, and clinical practice	Knowledge<br>Skills<br>Expectation of benefit<br>Reinforcement<br>Emotion	NA	NA
**Wakeman et al[156]**	2016	General internists' attitudes, practices and preparedness related to substance use disorder	Knowledge<br>Skills<br>Expectation of benefit<br>Emotion	NA	NA
**Wakeman et al[157]**	2016	Attitudes, practices, and preparedness to care for patients with substance use disorder: results from a survey of general internists	Knowledge<br>Skills	NA	NA

© 2024 Campopiano von Klimo M et al. *JAMA Network Open.*

Author	Year	Title	Reasons for reluctance cited	Factors impacting study quality (e.g., validity or precision of results)[a]	
				Theoretical framework[b]	Survey development best practices[c]
**Walley et al[158]**	2008	Office-based management of opioid dependence with buprenorphine: clinical practices and barriers	Knowledge<br>Reinforcement<br>Institutional environment<br>No demand	NA	NA
**Walther et al[159]**	2008	Teaching hospital staff about hazardous drinking: the effect of a single intervention	Knowledge<br>Skills<br>Relationship	NA	NA
**Warburg et al[160]**	1987	Residents' attitudes, knowledge, and behavior regarding diagnosis and treatment of alcoholism	Knowledge<br>Skills<br>Professional role/identity<br>Emotion	NA	NA
**Weinberger et al[161]**	2008	Survey of clinician attitudes toward smoking cessation for psychiatric and substance abusing clients	Knowledge<br>Expectation of benefit<br>Relationship<br>Cost to patient	NA	NA
**West et al[162]**	2009	Alcohol/other drug problems screening and intervention by rehabilitation physicians	Social influences	NA	NA
**Wilson et al[163]**	2011	Intervention against excessive alcohol consumption in primary health care: a survey of GPs' attitudes and practices in England 10 years on	Skills<br>Expectation of benefit<br>Reinforcement<br>Cognitive capacity<br>Institutional environment<br>Social influences<br>Relationship	NA	Piloted survey

© 2024 Campopiano von Klimo M et al. *JAMA Network Open.*

Author	Year	Title	Reasons for reluctance cited	Factors impacting study quality (e.g., validity or precision of results)[a]	
				Theoretical framework[b]	Survey development best practices[c]
**Wilson et al[164]**	2016	Internal medicine resident knowledge, attitudes, and barriers to naloxone prescription in hospital and clinic settings	Knowledge<br>Skills<br>Expectation of benefit<br>Cognitive capacity<br>Relationship<br>No demand<br>Cost to patient	NA	NA
**Winograd et al[165]**	2017	Medical providers' knowledge and concerns about opioid overdose education and take-home naloxone rescue kits within Veterans Affairs health care medical treatment settings	Knowledge<br>Skills<br>Institutional environment<br>Emotion<br>Relationship	NA	Target audience involved in survey development
**Wolk et al[166]**	2019	Perspectives and practice in the identification and treatment of opioid use, alcohol use, and depressive disorders	Knowledge<br>Skills<br>Professional role/identity<br>Social influences	NA	NA
**Wombacher et al[167]**	2019	Communication apprehension mediates the effects of past experience discussing substance use on child and adolescent psychiatrists' self-efficacy	Skills<br>Emotion<br>Relationship	Social Cognitive Theory	NA
**Yan et al[168]**	2008	Smoking behavior, knowledge, attitudes and	Knowledge<br>Skills<br>Social influences	NA	Target audience involved in

© 2024 Campopiano von Klimo M et al. *JAMA Network Open.*

Author	Year	Title	Reasons for reluctance cited	Factors impacting study quality (e.g., validity or precision of results)[a]	
				Theoretical framework[b]	Survey development best practices[c]
		practice among health care providers in Changsha city, China	Relationship		survey development<br><br>Piloted survey
**Zellman et al[169]**	1999	Physician response to prenatal substance exposure	Knowledge<br>Skills<br>Expectation of benefit<br>Reinforcement<br>Cognitive capacity<br>Institutional environment<br>Social influences<br>Relationship	NA	NA
**Zuckerman et al[170]**	2021	Physician attitudes on buprenorphine induction in the emergency department: results from a multistate survey	Knowledge<br>Skills<br>Reinforcement<br>Cognitive capacity<br>Institutional environment<br>Social influences	NA	Target audience involved in survey development<br><br>Piloted survey
**Study type: qualitative (82 articles)**					
**Abraham et al[171]**	2017	Providers' perspectives on barriers and facilitators to connecting women veterans to alcohol-related care from primary care	Knowledge<br>Cognitive capacity<br>Institutional environment	Consolidated Framework for Implementation Research	NA
**Abram & McCourt[172]**	1964	Interaction of physicians with emergency ward alcoholic patients	Professional role/identity<br>Expectation of benefit<br>Reinforcement<br>Emotion	NA	NA
**Aira et al[173]**	2003	Factors influencing inquiry about	Knowledge<br>Skills	NA	NA

© 2024 Campopiano von Klimo M et al. *JAMA Network Open.*

Author	Year	Title	Reasons for reluctance cited	Factors impacting study quality (e.g., validity or precision of results)[a]	
				Theoretical framework[b]	Survey development best practices[c]
		patients' alcohol consumption by primary health care physicians: qualitative semi-structured interview study	Professional role/identity<br>Expectation of benefit<br>Cognitive capacity<br>Institutional environment<br>Social influences		
**Alanis-Hirsch et al[174]**	2015	Extended-release naltrexone: a qualitative analysis of barriers to routine use	Knowledge<br>Skills<br>Expectation of benefit<br>Reinforcement<br>Institutional environment	Consolidated Framework for Implementation Research	NA
**Allen et al[175]**	2020	Substance Use Stigma, Primary Care, and the New York State Prescription Drug Monitoring Program	Knowledge<br>Skills<br>Professional role/identity<br>Expectation of benefit<br>Institutional environment<br>Social influences<br>Emotion	NA	NA
**Andraka-Christou & Capone[176]**	2018	A qualitative study comparing physician-reported barriers to treating addiction using buprenorphine and extended-release naltrexone in U.S. office-based practices	Knowledge<br>Skills<br>Professional role/identity<br>Reinforcement<br>Cognitive capacity<br>Institutional environment<br>Social influences<br>Emotion<br>Other	NA	NA
**Arborelius & Damström Thakker[177]**	1995	Why is it so difficult for general practitioners to discuss alcohol with patients	Expectation of benefit<br>Reinforcement<br>Cognitive capacity<br>Emotion	NA	NA

© 2024 Campopiano von Klimo M et al. *JAMA Network Open.*

Author	Year	Title	Reasons for reluctance cited	Factors impacting study quality (e.g., validity or precision of results)[a]	
				Theoretical framework[b]	Survey development best practices[c]
			Relationship		
**Barry et al[178]**	2009	Integrating buprenorphine treatment into office-based practice: a qualitative study	Knowledge<br>Skills<br>Professional role/identity<br>Reinforcement<br>Cognitive capacity<br>Institutional environment<br>Emotion	NA	NA
**Bartholomew & Bute[179]**	2023	Exploring internal medicine interns' educational experiences on opioid addiction: a narrative analysis	Knowledge<br>Expectation of benefit<br>Institutional environment<br>Emotion	Narrative Theorizing: symbolic actions, self-reflection, and sensemaking	NA
**Bar-Zeev et al[180]**	2019	Overcoming challenges to treating tobacco use during pregnancy - a qualitative study of Australian general practitioners' barriers	Knowledge<br>Expectation of benefit<br>Skills<br>Cognitive capacity<br>Social influences<br>Relationship	Theoretical domains framework (interview guide)	
**Beich et al[181]**	2002	Screening and brief intervention for excessive alcohol use: qualitative interview study of the experiences of general practitioners	Skills<br>Professional role/identity<br>Expectation of benefit<br>Institutional environment<br>Relationship	NA	NA
**Bell et al[182]**	2012	Physician advice for smoking cessation in primary care: time for a paradigm shift	Professional role/identity<br>Expectation of benefit<br>Reinforcement<br>Cognitive capacity	NA	NA

© 2024 Campopiano von Klimo M et al. *JAMA Network Open.*

Author	Year	Title	Reasons for reluctance cited	Factors impacting study quality (e.g., validity or precision of results)[a]	
				Theoretical framework[b]	Survey development best practices[c]
			Institutional environment Relationship		
**Binswanger et al[183]**	2015	Overdose education and naloxone for patients prescribed opioids in primary care: a qualitative study of primary care staff	Knowledge Skills Reinforcement Cognitive capacity Institutional environment	Theory of Planned Behavior; Health Belief Model	NA
**Blevins et al[184]**	2018	Gaps in the substance use disorder treatment referral process: provider perceptions	Knowledge Institutional environment	NA	NA
**Bounthavong et al[185]**	2020	Providers' perceptions on barriers and facilitators to prescribing naloxone for patients at risk for opioid overdose after implementation of a national academic detailing program: a qualitative assessment	Cognitive capacity Institutional environment Relationship	Theory of Planned Behavior, Framework for Effective Implementati on	NA
**Chichetto et al[186]**	2019	HIV care provider perceptions and approaches to managing unhealthy alcohol use in primary HIV care settings: a qualitative study	Skills Expectation of benefit Institutional environment Social influences	NA	NA

© 2024 Campopiano von Klimo M et al. *JAMA Network Open.*

Author	Year	Title	Reasons for reluctance cited	Factors impacting study quality (e.g., validity or precision of results)[a]	
				Theoretical framework[b]	Survey development best practices[c]
**Cunningham et al[187]**	2006	Attending physicians' and residents' attitudes and beliefs about prescribing buprenorphine at an urban teaching hospital	Knowledge<br>Skills<br>Professional role/identity<br>Cognitive capacity<br>Institutional environment	NA	NA
**Dong et al[188]**	2021	Emergency physician perspectives on initiating buprenorphine/naloxone in the emergency department: a qualitative study	Knowledge<br>Skills<br>Professional role/identity<br>Reinforcement<br>Cognitive capacity<br>Institutional environment<br>Emotion<br>Relationship	NA	NA
**Edsall et al[189]**	2021	Provider perspectives on integration of substance use disorder and HIV care in Vietnam: a qualitative study	Knowledge<br>Skills<br>Cognitive capacity<br>Institutional environment<br>Social influences	NA	NA
**El-Shahawy et al[190]**	2016	Primary care physicians' beliefs and practices regarding e-cigarette use by patients who smoke: a qualitative assessment	Knowledge<br>Institutional environment	Theory of Reasoned action	NA
**Fraeyman et al[191]**	2016	How to overcome hurdles in opiate substitution treatment? A qualitative study with general	Knowledge<br>Skills<br>Institutional environment<br>Emotion<br>Other	NA	NA

© 2024 Campopiano von Klimo M et al. *JAMA Network Open.*

Author	Year	Title	Reasons for reluctance cited	Factors impacting study quality (e.g., validity or precision of results)[a]	
				Theoretical framework[b]	Survey development best practices[c]
		practitioners in Belgium			
**Gatewood et al[192]**	2016	Academic physicians' and medical students' perceived barriers toward bystander administered naloxone as an overdose prevention strategy	Knowledge<br>Skills<br>Professional role/identity<br>Institutional environment<br>Social influences<br>Relationship	Grounded theory approach	NA
**Green et al[193]**	2014	A qualitative study of the adoption of buprenorphine for opioid addiction treatment	Professional role/identity<br>Expectation of benefit<br>Cognitive capacity<br>Institutional environment<br>Social influences<br>Emotion	NA	NA
**Haug et al[194]**	2016	Assessment of provider attitudes toward #naloxone on Twitter	Expectation of benefit<br>Emotion	Grounded theory approach	NA
**Herzig et al[195]**	2006	Seizing the 9-month moment: addressing behavioral risks in prenatal patients	Expectation of benefit<br>Cognitive capacity<br>Social influences<br>Relationship	NA	NA
**Herzig et al[196]**	2006	Comparing prenatal providers' approaches to four different risks: alcohol, tobacco, drugs, and domestic violence	Knowledge<br>Skills<br>Social influences<br>Emotion<br>Relationship	NA	NA

© 2024 Campopiano von Klimo M et al. *JAMA Network Open.*

Author	Year	Title	Reasons for reluctance cited	Factors impacting study quality (e.g., validity or precision of results)[a]	
				Theoretical framework[b]	Survey development best practices[c]
**Holland et al[197]**	2019	Emergency department physicians' and pharmacists' perspectives on take-home naloxone	Knowledge<br>Skills<br>Cognitive capacity<br>Institutional environment	NA	NA
**Hunter et al[198]**	2021	Clinician perspectives on methadone service delivery and the use of telemedicine during the COVID-19 pandemic: a qualitative study	Expectation of benefit<br>Institutional environment<br>Social influences<br>Emotion<br>Relationship	NA	NA
**Hutchinson et al[199]**	2014	Barriers to primary care physicians prescribing buprenorphine	Skills<br>Cognitive capacity<br>Institutional environment<br>Social influences<br>No demand	NA	NA
**Hutchinson & Rosenblatt[200]**	2013	Understanding and overcoming barriers to office-based physicians' treatment of opioid addiction [Abstract]	Knowledge<br>Skills<br>Cognitive capacity<br>Institutional environment<br>Social influences	NA	NA
**Hyland et al[201]**	2021	Treatment of alcohol dependence in Swedish primary care: perceptions among general practitioners	Knowledge<br>Skills<br>Cognitive capacity<br>Institutional environment<br>Social influences<br>Relationship	NA	NA
**Johansson et al[202]**	2005	Factors influencing GPs' decisions regarding screening for	Knowledge<br>Skills<br>Cognitive capacity<br>Relationship	NA	NA

© 2024 Campopiano von Klimo M et al. *JAMA Network Open.*

Author	Year	Title	Reasons for reluctance cited	Factors impacting study quality (e.g., validity or precision of results)[a]	
				Theoretical framework[b]	Survey development best practices[c]
		high alcohol consumption: a focus group study in Swedish primary care			
**Kaner et al[203]**	2006	Seeing through the glass darkly? A qualitative exploration of GPs' drinking and their alcohol intervention practices	Professional role/identity<br>Institutional environment<br>Social influences<br>Relationship	NA	NA
**Kennedy et al[204]**	2018	Resident trainee reflections on patients with substance use disorder after attending a mutual support group meeting	Skills<br>Expectation of benefit<br>Emotion	NA	NA
**Kenny & O'Carroll[205]**	2012	The use of psychotherapeutic interventions by primary care GPs in Ireland in the treatment of their methadone patients: a grounded theory study	Institutional environment<br>Social influences<br>Relationship	Ground theory	NA
**Kersnik et al[206]**	2009	What may stimulate general practitioners to undertake screening and brief intervention for excess alcohol consumption in Slovenia? A focus group study	Knowledge<br>Skills<br>Expectation of benefit<br>Reinforcement<br>Cognitive capacity<br>Institutional environment<br>Social influences<br>Emotion	NA	NA

© 2024 Campopiano von Klimo M et al. *JAMA Network Open.*

Author	Year	Title	Reasons for reluctance cited	Factors impacting study quality (e.g., validity or precision of results)[a]	
				Theoretical framework[b]	Survey development best practices[c]
**Ketterer et al[207]**	2014	What factors determine Belgian general practitioners' approaches to detecting and managing substance abuse? A qualitative study based on the I-Change Model	Knowledge<br>Skills<br>Professional role/identity<br>Expectation of benefit<br>Institutional environment<br>Emotion<br>Relationship	Integrated model of change (DeVrie's)	NA
**Kilgore et al[208]**	2021	Provider attitudes and practices on treating tobacco dependence in New York City after 10 years of comprehensive tobacco control efforts	Knowledge<br>Skills<br>Expectation of benefit<br>Reinforcement<br>Cognitive capacity	NA	NA
**Klimas et al[209]**	2015	Alcohol screening among opioid agonist patients in a primary care clinic and an opioid treatment program	Skills<br>Reinforcement<br>Relationship	NA	NA
**Lambrechts et al[210]**	2015	The approach taken to substance abuse by occupational physicians: A qualitative study on influencing factors	Professional role/identity<br>Institutional environment<br>Social influences<br>Relationship	Integrated model of change (DeVrie's)	NA
**Lid et al[211]**	2015	When general practitioners talk about alcohol: exploring facilitating and hampering factors for	Cognitive capacity<br>Institutional environment<br>Other	NA	NA

© 2024 Campopiano von Klimo M et al. *JAMA Network Open.*

Author	Year	Title	Reasons for reluctance cited	Factors impacting study quality (e.g., validity or precision of results)[a]	
				Theoretical framework[b]	Survey development best practices[c]
		pragmatic case finding			
**Lin & Detels[212]**	2011	A qualitative study exploring the reason for low dosage of methadone prescribed in the MMT clinics in China	Knowledge<br>Institutional environment<br>Relationship	NA	NA
**Mabood et al[213]**	2013	Experiences of pediatric emergency physicians in providing alcohol-related care to adolescents in the emergency department	Professional role/identity<br>Expectation of benefit<br>Institutional environment<br>Social influences<br>Emotion	Moustakas'phenomenological method and van Manen's approach to hermeneutics	NA
**Mark et al[214]**	2003	Barriers to the use of medications to treat alcoholism	Knowledge<br>Professional role/identity<br>Expectation of benefit<br>Cognitive capacity<br>Institutional environment<br>Social influences<br>Emotion<br>Cost to patient	Theory of Planned Behavior	NA
**Martin et al[215]**	2019	Emergency department-initiated medication-assisted treatment: lessons learned one year after launch	Knowledge<br>Skills<br>Professional role/identity<br>Cognitive capacity<br>Institutional environment	NA	NA
**Mathew et al[216]**	2009	Knowledge, attitudes, and practices of physicians in Tomsk Oblast	Knowledge<br>Skills<br>Professional role/identity	NA	NA

© 2024 Campopiano von Klimo M et al. *JAMA Network Open.*

Author	Year	Title	Reasons for reluctance cited	Factors impacting study quality (e.g., validity or precision of results)[a]	
				Theoretical framework[b]	Survey development best practices[c]
		tuberculosis services regarding alcohol use among tuberculosis patients in Tomsk, Russia	Expectation of benefit<br>Reinforcement<br>Institutional environment<br>Social influences<br>Emotion<br>Relationship		
**Mathis et al[217]**	2020	Provider-patient communication about prescription drug abuse: a qualitative analysis of the perspective of prescribers	Knowledge<br>Skills<br>Cognitive capacity<br>Institutional environment<br>Social influences<br>Emotion	Social Cognitive Theory; communication theory research	NA
**May et al[218]**	2008	Identification of barriers that impede the implementation of nicotine replacement therapy in the acute cardiac care setting	Knowledge<br>Professional role/identity<br>Expectation of benefit<br>Institutional environment<br>Other	NA	NA
**McCambridge et al[219]**	2004	Encouraging GP alcohol intervention: pilot study of change-orientated reflective listening (CORL)	Cognitive capacity	NA	NA
**McKeown et al[220]**	2003	A qualitative study of GPs' attitudes to drug misusers and drug misuse services in primary care	Knowledge<br>Expectation of benefit<br>Cognitive capacity<br>Institutional environment<br>Social influences<br>Emotion	NA	NA

© 2024 Campopiano von Klimo M et al. *JAMA Network Open.*

Author	Year	Title	Reasons for reluctance cited	Factors impacting study quality (e.g., validity or precision of results)[a]	
				Theoretical framework[b]	Survey development best practices[c]
**McMurphy et al**[221]	2006	Clinic-based treatment for opioid dependence: a qualitative inquiry	Knowledge<br>Skills<br>Reinforcement<br>Cognitive capacity<br>Institutional environment<br>Emotion<br>Relationship	NA	NA
**McNabb et al**[222]	2006	Diagnosing drug-seeking behaviour in an adult emergency department	Expectation of benefit<br>Reinforcement<br>Cognitive capacity<br>Emotion<br>Relationship	NA	NA
**McNeely et al**[223]	2017	Barriers and facilitators affecting the implementation of substance use screening in primary care clinics: a qualitative study of patients, providers, and staff	Knowledge<br>Skills<br>Cognitive capacity<br>Institutional environment<br>Social influences<br>Relationship	Knowledge to Action Framework	NA
**Meijer et al**[224]	2018	"It's on everyone's plate": a qualitative study into physicians' perceptions of responsibility for smoking cessation	Skills<br>Professional role/identity<br>Expectation of benefit<br>Reinforcement<br>Cognitive capacity<br>Institutional environment<br>Social influences<br>Relationship	Framework Method	NA
**Miller et al**[225]	2006	Initial steps taken by nine primary care practices to	Knowledge<br>Cognitive capacity	Practice Partner Research Network-	NA

© 2024 Campopiano von Klimo M et al. *JAMA Network Open.*

Author	Year	Title	Reasons for reluctance cited	Factors impacting study quality (e.g., validity or precision of results)[a]	
				Theoretical framework[b]	Survey development best practices[c]
		implement alcohol screening guidelines with hypertensive patients: the AA-TRIP project	Institutional environment Social influences Relationship	Translating Research into Practice	
**Miner et al[226]**	1996	Barriers to screening and counseling pregnant women for alcohol use	Skills Cognitive capacity Institutional environment Social influences Emotion Relationship	Systems Model of Clinical Preventive Care	NA
**Molfenter et al[227]**	2015	Implementing buprenorphine in addiction treatment: payer and provider perspectives in Ohio	Knowledge Professional role/identity Reinforcement Institutional environment Social influences Emotion	NA	NA
**Mudd et al[228]**	2020	Qualitative exploration of barriers to alcohol management in patients with chronic disease in a regional setting	Knowledge Skills Professional role/identity Expectation of benefit Reinforcement Institutional environment Social influences Relationship	NA	NA
**Mules et al[229]**	2012	Addressing patient alcohol use: a view from general practice	Cognitive capacity Social influences Relationship	NA	NA
**O'Donnell & Kaner[230]**	2017	Are brief alcohol interventions adequately	Skills Professional role/identity	Normalization process theory (type	NA

© 2024 Campopiano von Klimo M et al. *JAMA Network Open.*

Author	Year	Title	Reasons for reluctance cited	Factors impacting study quality (e.g., validity or precision of results)[a]	
				Theoretical framework[b]	Survey development best practices[c]
		embedded in UK primary care? A qualitative study utilising normalisation process theory	Expectation of benefit<br>Cognitive capacity<br>Relationship<br>Other	of implementation theory)	
**Omole et al[231]**	2014	Implementing tobacco dependence treatment during clinical consultations: a qualitative study of clinicians' experiences, perceptions and behaviours in a South African primary health care setting	Knowledge<br>Skills<br>Expectation of benefit<br>Cognitive capacity<br>Institutional environment<br>Social influences	Social Cognitive Theory	NA
**Pflanz-Sinclair et al[232]**	2018	Physicians' experiences of SBIRT training and implementation for SUD management in primary care in the UAE: a qualitative study	Reinforcement<br>Cognitive capacity<br>Institutional environment<br>Social influences<br>Relationship	NA	NA
**Pilnick & Coleman[233]**	2010	'Do your best for me': the difficulties of finding a clinically effective endpoint in smoking cessation consultations in primary care	Skills<br>Professional role/identity<br>Expectation of benefit<br>Institutional environment<br>Relationship<br>Other	NA	NA
**Poplas Susic et al[234]**	2010	Why do general practitioners not screen and intervene regarding	Knowledge<br>Skills<br>Professional role/identity	NA	NA

© 2024 Campopiano von Klimo M et al. *JAMA Network Open.*

Author	Year	Title	Reasons for reluctance cited	Factors impacting study quality (e.g., validity or precision of results)[a]	
				Theoretical framework[b]	Survey development best practices[c]
		alcohol consumption in Slovenia? A focus group study	Cognitive capacity<br>Institutional environment<br>Social influences<br>Relationship		
**Quest & Rosenblatt[235]**	2011	Buprenorphine therapy for opioid addiction in rural Washington: the early adopters.	Social influences	NA	NA
**Rahm et al[236]**	2015	Facilitators and barriers to implementing screening, brief intervention, and referral to treatment (SBIRT) in primary care in integrated health care settings	Skills<br>Professional role/identity<br>Cognitive capacity<br>Institutional environment<br>Relationship	NA	NA
**Rindal et al[237]**	2012	There is no rock bottom: emergency physicians' approach and adaptations to alcohol intoxicated patients	Expectation of benefit<br>Institutional environment	Theory of Planned Behavior	NA
**Scarborough et al[238]**	2011	Opioid substitution therapy: a study of GP participation in prescribing	Cognitive capacity<br>Institutional environment<br>Emotion	NA	NA
**Tam et al[239]**	2013	Australian general practitioner perceptions of the detection and screening of at-risk drinking, and the role of	Knowledge<br>Institutional environment<br>Social influences<br>Emotion<br>Relationship	Straussian Grounded Theory	NA

© 2024 Campopiano von Klimo M et al. *JAMA Network Open.*

Author	Year	Title	Reasons for reluctance cited	Factors impacting study quality (e.g., validity or precision of results)[a]	
				Theoretical framework[b]	Survey development best practices[c]
		the AUDIT-C: a qualitative study			
**Taylor et al[240]**	2007	Prenatal screening for substance use and violence: findings from physician focus groups	Cognitive capacity<br>Institutional environment<br>Social influences<br>Relationship	NA	NA
**Tesema et al[241]**	2018	Training in office-based opioid treatment with buprenorphine in US residency programs: a national survey of residency program directors	Knowledge<br>Skills<br>Expectation of benefit<br>Reinforcement<br>Cognitive capacity<br>Institutional environment<br>Social influences<br>Emotion<br>Relationship<br>Cost to patient	Theory of Planned Behavior; Health belief model	NA
**Todd et al[242]**	2002	Barriers to optimal care for patients with coexisting substance use and mental health disorders	Knowledge<br>Skills<br>Professional role/identity<br>Expectation of benefit<br>Cognitive capacity<br>Institutional environment<br>Social influences<br>Emotion	NA	NA
**Van Hook et al[243]**	2007	The "six T's": barriers to screening teens for substance abuse in primary care	Knowledge<br>Skills<br>Cognitive capacity<br>Institutional environment<br>Social influences<br>Relationship	NA	NA
**Van Hout et al[244]**	2018	Optimising treatment in	Knowledge<br>Skills	NA	NA

© 2024 Campopiano von Klimo M et al. *JAMA Network Open.*

Author	Year	Title	Reasons for reluctance cited	Factors impacting study quality (e.g., validity or precision of results)[a]	
				Theoretical framework[b]	Survey development best practices[c]
		opioid dependency in primary care: results from a national key stakeholder and expert focus group in Ireland	Professional role/identity<br>Cognitive capacity<br>Institutional environment<br>Social influences<br>Emotion		
**van Schayck et al[245]**	2020	The experience of general practitioners with Very Brief Advice in the treatment of tobacco addiction	Cognitive capacity	NA	NA
**Varley et al[246]**	2020	Understanding barriers and facilitators to the uptake of best practices for the treatment of co-occurring chronic pain and opioid use disorder	Skills<br>Expectation of benefit<br>Cognitive capacity<br>Institutional environment<br>Social influences<br>Emotion	Consolidated Framework for Implementation Research	NA
**Webster et al[247]**	2020	Emergency physician attitudes on opioid use disorder and barriers to providing buprenorphine/naloxone	Knowledge<br>Skills<br>Cognitive capacity<br>Institutional environment<br>Social influences<br>Emotion	NA	NA
**Wiercigroch et al[248]**	2019	Management of opioid withdrawal: a qualitative examination of current practices and barriers to prescribing buprenorphine in a Canadian	Skills<br>Professional role/identity<br>Expectation of benefit<br>Cognitive capacity<br>Institutional environment<br>Emotion<br>Relationship	NA	NA

© 2024 Campopiano von Klimo M et al. *JAMA Network Open.*

Author	Year	Title	Reasons for reluctance cited	Factors impacting study quality (e.g., validity or precision of results)[a]	
				Theoretical framework[b]	Survey development best practices[c]
		emergency department			
**Wiercigroch et al[249]**	2021	A qualitative examination of the current management of opioid use disorder and barriers to prescribing buprenorphine in a Canadian emergency department	Knowledge<br>Skills<br>Institutional environment<br>Relationship	Constructivist Grounded Theory Approach	NA
**Williams et al[250]**	2018	Barriers to and facilitators of alcohol use disorder pharmacotherapy in primary care: a qualitative study in five VA clinics	Knowledge<br>Skills<br>Professional role/identity<br>Expectation of benefit<br>Institutional environment<br>Social influences<br>Emotion	Domains of Behavior Change	NA
**Wloch[251]**	2002	Role of the countryside general practitioner in recognition of reasons and circumstances of alcohol abuse by patients of Spzoz. Part I. Characteristics of staff and patients' resources in the outpatient health care in the Lublin voivodeship	Knowledge<br>Skills	NA	NA
**Wolf et al[252]**	1965	Social factors in the diagnosis of alcoholism. II.	Professional role/identity	NA	NA

© 2024 Campopiano von Klimo M et al. *JAMA Network Open.*

Author	Year	Title	Reasons for reluctance cited	Factors impacting study quality (e.g., validity or precision of results)[a]	
				Theoretical framework[b]	Survey development best practices[c]
		Attitudes of physicians	Social influences<br>Emotion<br>Relationship		
**Study type: mixed methods (27 articles)**					
**Chenworth et al[253]**	2020	Buprenorphine initiation in the emergency department: a thematic content analysis of a #firesidetox Tweetchat	Expectation of benefit<br>Institutional environment<br>Emotion	NA	NA
**Foster et al[254]**	2020	Providing incentive for emergency physician X-waiver training: an evaluation of program success and postintervention buprenorphine prescribing	Reinforcement<br>Cognitive capacity<br>Institutional environment	NA	NA
**Gordon et al[255]**	2011	Provider, patient, and family perspectives of adolescent alcohol use and treatment in rural settings	Skills<br>Professional role/identity<br>Expectation of benefit<br>Cognitive capacity<br>Social influences<br>Relationship	NA	NA
**Haffajee et al[256]**	2020	A mixed-method comparison of physician-reported beliefs about and barriers to treatment with medications for opioid use disorder	Knowledge<br>Skills<br>Expectation of benefit<br>Reinforcement<br>Institutional environment<br>Social influences<br>Emotion<br>No demand	NA	Piloted survey
**Hanna[257]**	1991	Attitudes toward problem	Emotion	NA	NA

© 2024 Campopiano von Klimo M et al. *JAMA Network Open.*

Author	Year	Title	Reasons for reluctance cited	Factors impacting study quality (e.g., validity or precision of results)[a]	
				Theoretical framework[b]	Survey development best practices[c]
		drinkers, revisited: patient-therapist factors contributing to the differential treatment of patients with alcohol problems			
**Harris et al[258]**	2013	Pharmacotherapy for alcohol dependence: perceived treatment barriers and action strategies among Veterans Health Administration service providers	Knowledge<br>Skills<br>Expectation of benefit<br>Cognitive capacity<br>Institutional environment<br>Social influences<br>Emotion	NA	NA
**Harutyunyan et al[259]**	2019	Perceived barriers of tobacco dependence treatment: a mixed-methods study among primary healthcare physicians in Armenia	Knowledge<br>Skills<br>Professional role/identity<br>Expectation of benefit<br>Institutional environment<br>Social influences<br>Relationship	NA	NA
**Hawk et al[260]**	2020	Barriers and facilitators to clinician readiness to provide emergency department-initiated buprenorphine	Knowledge<br>Skills<br>Institutional environment<br>Social influences	Promoting Action on Research Implementation in Health Services (PARiHS) framework	NA
**Im et al261**	2020	Emergency department clinicians' attitudes toward opioid use	Knowledge<br>Skills<br>Professional role/identity	NA	NA

© 2024 Campopiano von Klimo M et al. *JAMA Network Open.*

Author	Year	Title	Reasons for reluctance cited	Factors impacting study quality (e.g., validity or precision of results)[a]	
				Theoretical framework[b]	Survey development best practices[c]
		disorder and emergency department-initiated buprenorphine treatment: a mixed-methods study	Expectation of benefit<br>Reinforcement<br>Cognitive capacity<br>Institutional environment<br>Social influences<br>Emotion		
**Joudrey et al[262]**	2019	A pre-implementation study of hospital-based health professional perspectives on prescribing of medications for alcohol use disorder	Knowledge<br>Skills<br>Expectation of benefit<br>Social influences<br>Emotion	Implementation Science framework	NA
**Joudrey et al[263]**	2020	Inpatient adoption of medications for alcohol use disorder: a mixed-methods formative evaluation involving key stakeholders	Knowledge<br>Skills<br>Expectation of benefit<br>Reinforcement<br>Cognitive capacity<br>Institutional environment<br>Social influences<br>Emotion<br>Other	Consolidated Framework for Implementation Research	NA
**Kathuria et al[264]**	2019	Patient and physician perspectives on treating tobacco dependence in hospitalized smokers with substance use disorders: a mixed methods study	Expectation of benefit<br>Cognitive capacity<br>Institutional environment<br>Social influences<br>Emotion	NA	NA
**Matusow et al[265]**	2012	Factors associated with	Knowledge	NA	NA

© 2024 Campopiano von Klimo M et al. *JAMA Network Open.*

Author	Year	Title	Reasons for reluctance cited	Factors impacting study quality (e.g., validity or precision of results)[a]	
				Theoretical framework[b]	Survey development best practices[c]
		mental health clinicians' referrals to 12-step groups	Expectation of benefit<br>Social influences<br>Relationship		
**McCausland et al[266]**	2020	Current practice in withdrawal management: opportunities to improve treatment of opioid use disorder	Knowledge<br>Skills<br>Reinforcement<br>Institutional environment<br>Social influences<br>Patient refusal	NA	NA
**Oros et al[267]**	2021	Facilitators and barriers to utilization of medications for opioid use disorder in primary care in South Carolina	Knowledge<br>Skills<br>Professional role/identity<br>Expectation of benefit<br>Cognitive capacity<br>Institutional environment<br>Social influences<br>Emotion<br>Relationship	Theory of Planned Behavior	NA
**Palmer et al[268]**	2019	Barriers faced by physicians in screening for substance use disorders among adolescents	Skills<br>Expectation of benefit<br>Reinforcement<br>Cognitive capacity<br>Institutional environment<br>Social influences<br>Relationship	NA	NA
**Panda et al[269]**	2013	Perception and practices of physicians in addressing the smokeless tobacco	Knowledge<br>Expectation of benefit<br>Social influences<br>Relationship	NA	NA

© 2024 Campopiano von Klimo M et al. *JAMA Network Open.*

Author	Year	Title	Reasons for reluctance cited	Factors impacting study quality (e.g., validity or precision of results)[a]	
				Theoretical framework[b]	Survey development best practices[c]
		epidemic: findings from two states in India			
**Penm et al**[270]	2018	Combatting opioid overdoses in Ohio: emergency department physicians' prescribing patterns and perceptions of naloxone	Knowledge<br>Professional role/identity<br>Expectation of benefit<br>Emotion	NA	NA
**Quest et al**[271]	2012	Buprenorphine therapy for opioid addiction in rural Washington: the experience of the early adopters	Expectation of benefit<br>Reinforcement<br>Institutional environment<br>Social influences	NA	Piloted survey
**Roche & Richard**[272]	1991	Doctors' willingness to intervene in patients' drug and alcohol problems	Skills<br>Expectation of benefit<br>Institutional environment<br>Social influences	NA	NA
**Rohman et al**[273]	1987	The response of primary care physicians to problem drinkers	Skills<br>Expectation of benefit<br>Reinforcement<br>Cognitive capacity<br>Social influences<br>Emotion	NA	NA
**Rowan & Galasso**[274]	2000	Identifying office resource needs of Canadian physicians to help prevent, assess and treat patients with	Knowledge<br>Skills<br>Professional role/identity<br>Reinforcement<br>Institutional environment	NA	Piloted survey

© 2024 Campopiano von Klimo M et al. *JAMA Network Open.*

Author	Year	Title	Reasons for reluctance cited	Factors impacting study quality (e.g., validity or precision of results)[a]	
				Theoretical framework[b]	Survey development best practices[c]
		substance use and pathological gambling disorders			
**Segnan et al[275]**	1992	Preventive practices of general practitioners in Torino, Italy	Knowledge<br>Skills<br>Expectation of benefit<br>Reinforcement<br>Emotion<br>Other	NA	Piloted survey
**Stöver[276]**	2011	Barriers to opioid substitution treatment access, entry and retention: a survey of opioid users, patients in treatment, and treating and non-treating physicians	Knowledge<br>Professional role/identity<br>Reinforcement<br>Institutional environment<br>Social influences<br>Emotion<br>No demand<br>Other	NA	NA
**Van Zyl[277]**	2016	Doctors' views of disulfiram and their response to relapse in alcohol-dependent patients, Free State, 2009	Knowledge<br>Skills<br>Expectation of benefit<br>Reinforcement<br>Institutional environment<br>Emotion<br>Relationship	NA	NA
**Wamsley et al[278]**	2014	Teaching residents screening, brief intervention and referral to treatment skills for alcohol use: using chart-stimulated recall to assess curricular impact	Knowledge<br>Skills<br>Cognitive capacity<br>Social influences<br>Relationship	NA	NA
**Yilani et al[279]**	2020	Medication-assisted treatment in	Knowledge<br>Professional role/identity	NA	NA

© 2024 Campopiano von Klimo M et al. *JAMA Network Open.*

Author	Year	Title	Reasons for reluctance cited	Factors impacting study quality (e.g., validity or precision of results)[a]	
				Theoretical framework[b]	Survey development best practices[c]
		alcohol use disorder: can education and EMR interventions increase prescriptions?	Expectation of benefit		
**Study type: meta-analysis (2 articles)**					
**Anderson et al[280]**	2004	Attitudes and managing alcohol problems in general practice: an interaction analysis based on findings from a WHO collaborative study	Knowledge<br>Skills<br>Professional role/identity<br>Expectation of benefit<br>Reinforcement	NA	NA
**Mitchell et al[281]**	2012	Clinical recognition and recording of alcohol disorders by clinicians in primary and secondary care: meta-analysis	Knowledge<br>Skills<br>Reinforcement<br>Cognitive capacity<br>Institutional environment<br>Social influences<br>Emotion<br>Relationship	NA	NA
**Study type: clinical study (2 articles)**					
**Mertens et al[282]**	2011	Structural and attitudinal factors affecting SBIRT implementation in adult primary care	Knowledge<br>Expectation of benefit<br>Cognitive capacity<br>Institutional environment	NA	NA
**Muench et al[283]**	2015	Perceptions of clinical team members toward implementation of SBIRT processes	Knowledge<br>Skills<br>Expectation of benefit<br>Reinforcement<br>Cognitive capacity<br>Institutional environment	Consolidated Framework for Implementati on Research	NA

© 2024 Campopiano von Klimo M et al. *JAMA Network Open.*

Author	Year	Title	Reasons for reluctance cited	Factors impacting study quality (e.g., validity or precision of results)[a]	
				Theoretical framework[b]	Survey development best practices[c]
			Relationship		

[a] Survey development best practices are defined as whether the target audience was involved in survey development and if it was piloted. Best practices and theoretical frameworks may have been applied but not reported in the published article. Data is presented only where it could be identified in the publication.
[a] NA in this column indicates that the Theoretical Framework is not applicable: no data were identified in the publication.
[a] NA in this column indicates that Survey Development Best Practices are not applicable: no data were identified in the publication.

© 2024 Campopiano von Klimo M et al. *JAMA Network Open.*

eTable 2. Distribution of Publications by Year

Year of Publication	Number of Publications, No., (%) (N=283)
1960-1969	2 (0.7)
1970-1979	5 (1.8)
1980-1989	7 (2.5)
1990-1999	21 (7.4)
2000-2009	66 (23.3)
2000	4 (1.4)
2001	1 (0.4)
2002	6 (2.1)
2003	7 (2.5)
2004	3 (1.1)
2005	4 (1.4)
2006	12 (4.2)
2007	6 (2.1)
2008	12 (4.2)
2009	11(3.9)
2010-2021	181(64.0)
2010	9 (3.2)
2011	13 (4.6)
2012	6 (2.1)
2013	12 (4.2)
2014	5 (1.8)
2015	15 (5.3)
2016	19 (6.7)
2017	8 (2.8)
2018	22 (7.8)
2019	20 (7.1)
2020	31 (11.0)
2021	21 (7.4)
Missing	1 (0.4)

© 2024 Campopiano von Klimo M et al. *JAMA Network Open.*

eTable 3. Physician Practice Settings Reported in Publications Since 2010

Year (N)[a]	Emergency Department, No. (%)[b]	Inpatient, No. (%)[b]	Office, No. (%)[b]	Addiction Treatment, No. (%)[b]
2010 (9)	1 (11.1)	1 (11.1)	7 (77.8)	0 (0.0)
2011 (13)	0 (0.0)	3 (23.1)	7 (53.8)	2 (15.4)
2012 (6)	1 (16.7)	1 (16.7)	4 (66.7)	1 (16.7)
2013 (12)	2 (16.7)	3 (25.0)	10 (83.3)	2 (16.7)
2014 (5)	1 (20.0)	0 (0.0)	4 (80.0)	0 (0.0)
2015 (15)	1 (6.7)	1 (6.7)	8 (53.3)	1 (6.7)
2016 (19)	3 (15.8)	1 (5.3)	5 (26.3)	1 (5.3)
2017 (8)	0 (0.0)	2 (25.0)	6 (75.0)	0 (0.0)
2018 (22)	5 (22.7)	4 (18.2)	12 (54.5)	2 (9.1)
2019 (20)	7 (35.0)	4 (20.0)	5 (25.0)	0 (0.0)
2020 (31)	9 (29.0)	8 (25.8)	12 (38.7)	0 (0.0)
2021 (21)	5 (23.8)	2 (9.5)	6 (28.6)	1 (4.8)

[a] The denominator represents the number of included studies published that year.
[b] Studies may have asked about more than one practice setting.

© 2024 Campopiano von Klimo M et al. *JAMA Network Open.*

eTable 4. Drug Types Reported Since 2000

	Drug reported No (%)		
Year (N)[a]	Alcohol No. (%)[b]	Opioids No. (%)[b]	Nicotine No. (%)[b]
2000 (4)	1 (25.0)	2 (50.0)	0 (0.0)
2001 (1)	1 (100.0)	0 (0.0)	0 (0.0)
2002 (6)	5 (83.3)	0 (0.0)	0 (0.0)
2003 (7)	6 (85.7)	1 (14.3)	0 (0.0)
2004 (3)	2 (66.7)	1 (33.3)	1 (33.3)
2005 (4)	2 (50.0)	1 (25.0)	1 (25.0)
2006 (12)	5 (41.7)	5 (41.7)	3 (25.0)
2007 (6)	3 (50.0)	2 (33.3)	0 (0.0)
2008 (12)	5 (41.7)	4 (33.3)	3 (25.0)
2009 (11)	5 (45.5)	3 (27.3)	3 (27.3)
2010 (9)	4 (44.4)	2 (22.2)	2 (22.2)
2011 (13)	4 (30.8)	6 (46.2)	6 (46.2)
2012 (6)	3 (50.0)	2 (33.3)	0 (0.0)
2013 (12)	8 (66.7)	5 (41.7)	1 (8.3)
2014 (5)	1 (20.0)	3 (60.0)	1 (20.0)
2015 (15)	8 (53.3)	7 (46.7)	2 (13.3)
2016 (19)	9 (47.4)	8 (42.1)	1 (5.3)
2017 (8)	5 (62.5)	5 (62.5)	0 (0.0)
2018 (22)	6 (27.3)	12 (54.5)	5 (22.7)
2019 (20)	4 (20.0)	11 (55.0)	3 (15.0)
2020 (31)	9 (29.0)	18 (58.1)	4 (12.9)
2021 (21)	2 (9.5)	16 (76.2)	3 (14.3)

[a] The denominator represents the number of included studies published that year.
[b] Studies may have asked about more than one drug or a drug not reported here.

© 2024 Campopiano von Klimo M et al. *JAMA Network Open.*

eTable 5. Publications Reporting Reasons for Reluctance From 2000 to 2021, When Asked

	No. / N (%)[a]									
Year	Knowledge	Skills	Professional Role/Identity	Expectation of Benefit	Reinforcement	Cognitive Capacity	Institutional Environment	Social Influence	Emotion	Relationship
2000	2/3 (66.7)	2/2 (100.0)	2/4 (50.0)	2/3 (66.7)	3/3 (100.0)	2/2 (100.0)	2/3 (66.7)	0/2 (0.0)	1/2 (50.0)	0/2 (0.0)
2001	0/0 (0.0)	1/1 100.0	1/1 (100.0)	0/0 (0.0)	0/0 (0.0)	1/1 (100.0)	0/0 (0.0)	0/0 (0.0)	0/0 (0.0)	0/0 (0.0)
2002	5/6 (83.3)	6/6 (100.0)	1/3 (33.3)	3/4 (75.0)	1/2 (50.0)	1/2 (50.0)	3/3 (100.0)	0/3 (0.0)	1/5 (20.0)	1/4 (25.0)
2003	7/7 (100.0)	2/5 (40.0)	3/6 (50.0)	6/6 (100.0)	0/3 (0.0)	3/3 (100.0)	6/6 (100.0)	5/5 (100.0)	3/5 (60.0)	1/4 (25.0)
2004	2/2 (100.0)	2/2 (100.0)	1/2 (50.0)	2/2 (100.0)	1/2 (50.0)	1/2 (50.0)	0/2 (0.0)	0/1 (0.0)	0/1 (0.0)	0/1 (0.0)
2005	3/4 (75.0)	4/4 (100.0)	1/4 (25.0)	2/3 (66.7)	2/3 (66.7)	4/4 (100.0)	2/4 (50.0)	1/3 (33.3)	1/2 (50.0)	1/2 (50.0)
2006	5/10 (50.0)	6/9 (66.7)	2/7 (28.6)	5/10 (50.0)	3/7 (42.9)	6/8 (75.0)	7/10 (70.0)	5/7 (71.4)	6/9 (66.7)	7/8 (87.5)
2007	4/5 (80.0)	4/5 (80.0)	1/3 (33.3)	1/3 (33.3)	2/4 (50.0)	4/4 (100.0)	5/5 (100.0)	4/4 (100.0)	2/5 (40.0)	3/3 (100.0)
2008	8/10 (80.0)	4/9 (44.4)	5/9 (55.6)	4/9 (44.4)	3/8 (37.5)	6/9 (66.7)	8/11 (72.7)	4/8 (50.0)	4/7 (57.1)	5/10 (50.0)
2009	7/10 (70.0)	7/10 (70.0)	2/8 (25.0)	6/9 (66.7)	5/5 (100.0)	4/5 (80.0)	6/6 (100.0)	7/7 (100.0)	9/9 (100.0)	2/4 (50.0)
2010	5/9 (55.6)	6/7 (85.7)	4/6 (66.7)	4/6 (66.7)	3/7 (42.9)	3/5 (60.0)	6/7 (85.7)	4/6 (66.7)	4/7 (57.1)	4/5 (80.0)
2011	7/11 (63.6)	7/13 (53.8)	2/11 (18.2)	6/11 (54.5)	2/8 (25.0)	8/11 (72.7)	7/8 (87.5)	5/9 (55.6)	1/9 (11.1)	5/10 (50.0)
2012	3/5 (60.0)	2/5 (40.0)	0/4 (0.0)	2/3 (66.7)	0/4 (0.0)	3/5 (60.0)	3/5 (60.0)	2/3 (66.7)	1/4 (25.0)	3/4 (75.0)
2013	8/11 (72.7)	5/9 (55.6)	2/5 (40.0)	4/6 (66.7)	2/5 (40.0)	3/6 (50.0)	6/9 (66.7)	7/7 (100.0)	6/10 (60.0)	2/5 (40.0)
2014	3/4 (75.0)	3/4 (75.0)	2/4 (50.0)	3/5 (60.0)	1/4 (25.0)	2/4 (50.0)	4/4 (100.0)	3/4 (75.0)	3/4 (75.0)	1/3 (33.3)
2015	8/14	8/14	4/11	9/15	8/12	6/11	9/11	2/11	2/10	6/10

© 2024 Campopiano von Klimo M et al. *JAMA Network Open.*

	No. / N (%)[a]									
**Year**	**Knowledge**	**Skills**	**Professional Role/Identity**	**Expectation of Benefit**	**Reinforcement**	**Cognitive Capacity**	**Institutional Environment**	**Social Influence**	**Emotion**	**Relationship**
	(51.1)	(57.1)	(36.4)	(60.0)	(66.7)	(54.5)	(81.8)	(18.2)	(20.0)	(60.0)
**2016**	13/17 (76.5)	11/15 (73.3)	5/13 (38.5)	10/15 (66.7)	5/10 (50.0)	7/12 (58.3)	10/14 (71.4)	7/12 (58.3)	8/11 (72.7)	6/10 (60.0)
**2017**	6/8 (75.0)	6/7 (85.7)	2/4 (50.0)	4/6 (66.7)	3/5 (60.0)	5/6 (83.3)	6/7 (85.7)	3/4 (75.0)	5/6 (83.3)	3/5 (60.0)
**2018**	16/18 (88.9)	17/19 (89.5)	9/17 (52.9)	10/15 (66.7)	6/13 (46.2)	14/16 (87.5)	13/16 (81.5)	11/15 (73.3)	12/14 (85.7)	8/12 (66.7)
**2019**	12/17 (70.6)	16/19 (84.2)	5/15 (33.3)	8/17 (47.1)	3/13 (23.1)	11/16 (68.8)	12/16 (75.0)	10/17 (58.8)	10/17 (58.8)	5/13 (38.5)
**2020**	23/28 (82.1)	22/27 (81.5)	8/24 (33.3)	14/23 (60.9)	10/17 (58.8)	17/21 (81.0)	24/26 (92.3)	17/22 (77.3)	12/22 (54.5)	9/20 (45.0)
**2021**	11/16 (68.8)	11/15 (73.3)	5/16 (31.3)	6/16 (37.5)	5/13 (38.5)	12/15 (80.0)	16/17 (94.1)	10/16 (62.5)	8/16 (50.0)	8/13 (61.5)

[a] The denominator (N) is the number studies that asked about a reason and is unique for each cell. Studies may have asked about more than 1 reason.

© 2024 Campopiano von Klimo M et al. *JAMA Network Open.*

eReferences

1. Aalto M, Hyvönen S, Seppä K. Do primary care physicians' own AUDIT scores predict their use of brief alcohol intervention? A cross-sectional survey. *Drug Alcohol Depend*. 2006;83(2):169-73.
2. Aalto M, Pekuri P, Seppä K. Primary health care nurses' and physicians' attitudes, knowledge and beliefs regarding brief intervention for heavy drinkers. *Addiction*. 2001;96(2):305-11.
3. Abed RT, Neira-Munoz E. A survey of general practitioners' opinion and attitude to drug addicts and addiction. *Br J Addict*. 1990;85(1):131-6.
4. Adams PJ, Powell A, McCormick R, et al. Incentives for general practitioners to provide brief interventions for alcohol problems. *N Z Med J*. 1997;110(1049):291-4.
5. An LC, Bernhardt TS, Bluhm J, et al. Treatment of tobacco use as a chronic medical condition: primary care physicians' self-reported practice patterns. *Prev Med*. 2004;38(5):574-85.
6. Andrilla CHA, Coulthard C, Larson EH. Barriers rural physicians face prescribing buprenorphine for opioid use disorder. *Ann Fam Med*. 2017;15(4):359-62.
7. Arfken CL, Johanson CE, di Menza S, et al. Expanding treatment capacity for opioid dependence with office-based treatment with buprenorphine: national surveys of physicians. *J Subst Abuse Treat*. 2010;39(2):96-104.
8. Bander KW, Goldman DS, Schwartz MA, et al. Survey of attitudes among three specialties in a teaching hospital toward alcoholics. *J Med Educ*. 1987;62(1):17-24.
9. Beletsky L, Ruthazer R, Macalino GE, et al. Physicians' knowledge of and willingness to prescribe naloxone to reverse accidental opiate overdose: challenges and opportunities. *J Urban Health*. 2007;84(1):126-36.
10. Bell K, Bowers M, McCullough L, et al. Physician advice for smoking cessation in primary care: time for a paradigm shift? *Critical Public Health*. 2012;22(1):9-24.
11. Bernstein E, Bernstein J, Feldman J, et al. An evidence based alcohol screening, brief intervention and referral to treatment (SBIRT) curriculum for emergency department (ED) providers improves skills and utilization. *Subst Abus*. 2007;28(4):79-92.
12. Besson J, Beck T, Wiesbeck G, et al. Opioid maintenance therapy in Switzerland: an overview of the Swiss IMPROVE study. *Swiss Med Wkly*. 2014;144:w13933.
13. Bradley KA, Curry SJ, Koepsell TD, et al. Primary and secondary prevention of alcohol problems: U.S. internist attitudes and practices. *J Gen Intern Med*. 1995;10(2):67-72.
14. Broderick KB, Kaplan B, Martini D, et al. Emergency physician utilization of alcohol/substance screening, brief advice and discharge: a 10-year comparison. *J Emerg Med*. 2015;49(4):400-7.
15. Calcaterra SL, Binswanger IA, Edelman EJ, et al. The impact of access to addiction specialist on attitudes, beliefs and hospital-based opioid use disorder related care: a survey of hospitalist physicians. *Subst Abus*. 2022;43(1):143-51.
16. Chan MF, Alsaidi Y, Al-Sumri S, et al. Knowledge, attitudes, practice and barriers of physicians to provide tobacco dependence treatment: a cluster analysis. *Pan Afr Med J*. 2021;38:193.
17. Cilluffo G, Ferrante G, Cutrera R, et al. Barriers and incentives for Italian paediatricians to become smoking cessation promoters: a GARD-Italy Demonstration Project. *J Thorac Dis*. 2020;12(11):6868-79.
18. Collins C, Finegan P, O'Riordan M. An online survey of Irish general practitioner experience of and attitude toward managing problem alcohol use. *BMC Fam Pract*. 2018;19(1):200.
19. Cotter TG, Ayoub F, King AC, et al. Practice habits, knowledge, and attitudes of hepatologists to alcohol use disorder medication: sobering gaps and opportunities. *Transplant Direct*. 2020;6(10):e603.
20. Cunningham CO, Kunins HV, Roose RJ, et al. Barriers to obtaining waivers to prescribe buprenorphine for opioid addiction treatment among HIV physicians. *J Gen Intern Med*. 2007;22(9):1325-9.

© 2024 Campopiano von Klimo M et al. *JAMA Network Open.*

21. Cunningham RM, Harrison SR, McKay MP, et al. National survey of emergency department alcohol screening and intervention practices. *Ann Emerg Med*. 2010;55(6):556-62.
22. Danielsson PE, Rivara FP, Gentilello LM, et al. Reasons why trauma surgeons fail to screen for alcohol problems. *Arch Surg*. 1999;134(5):564-8.
23. Day FL, Sherwood E, Chen TY, et al. Oncologist provision of smoking cessation support: a national survey of Australian medical and radiation oncologists. *Asia Pac J Clin Oncol*. 2018;14(6):431-8.
24. Deehan A, Taylor C, Strang J. The general practitioner, the drug misuser, and the alcohol misuser: major differences in general practitioner activity, therapeutic commitment, and 'shared care' proposals. *Br J Gen Pract*. 1997;47(424):705-9.
25. DeFlavio JR, Rolin SA, Nordstrom BR, et al. Analysis of barriers to adoption of buprenorphine maintenance therapy by family physicians. *Rural Remote Health*. 2015;15:3019.
26. Demmert A, Grothues JM, Rumpf HJ. Attitudes towards brief interventions to reduce smoking and problem drinking behaviour in gynaecological practice. *Public Health*. 2011;125(4):182-6.
27. Donovan CL. Factors predisposing, enabling and reinforcing routine screening of patients for preventing fetal alcohol syndrome: a survey of New Jersey physicians. *J Drug Educ*. 1991;21(1):35-42.
28. Duszynski KR, Nieto FJ, Valente CM. Reported practices, attitudes, and confidence levels of primary care physicians regarding patients who abuse alcohol and other drugs. *Md Med J*. 1995;44(6):439-46.
29. Ehrie J, Hartwell EE, Morris PE, et al. Survey of addiction specialists' use of medications to treat alcohol use disorder. *Front Psychiatry*. 2020;11:47.
30. Elliott EJ, Payne J, Haan E, et al. Diagnosis of foetal alcohol syndrome and alcohol use in pregnancy: a survey of paediatricians' knowledge, attitudes and practice. *J Paediatr Child Health*. 2006;42(11):698-703.
31. Elwy AR, Horton NJ, Saitz R. Physicians' attitudes toward unhealthy alcohol use and self-efficacy for screening and counseling as predictors of their counseling and primary care patients' drinking outcomes. *Subst Abuse Treat Prev Policy*. 2013;8:17.
32. Ferguson L, Ries R, Russo J. Barriers to identification and treatment of hazardous drinkers as assessed by urban/rural primary care doctors. *J Addict Dis*. 2003;22(2):79-90.
33. Fisher JC, Mason RL, Keeley KA, et al. Physicians and alcoholics. The effect of medical training on attitudes toward alcoholics. *J Stud Alcohol*. 1975;36(7):949-55.
34. Fisher JC, Keeley KA, Mason RL, et al. Physicians and alcoholics. Factors affecting attitudes of family-practice residents toward alcoholics. *J Stud Alcohol*. 1975;36(5):626-33.
35. Fortmann SP, Sallis JF, Magnus PM, et al. Attitudes and practices of physicians regarding hypertension and smoking: the Stanford Five City project. *Prev Med*. 1985;14(1)70-80.
36. Foti K, Heyward J, Tajanlangit M, et al. Primary care physicians' preparedness to treat opioid use disorder in the United States: a cross-sectional survey. *Drug Alcohol Depend*. 2021;225:108811.
37. Franz B, Dhanani LY, Brook DL. Physician blame and vulnerability: novel predictors of physician willingness to work with patients who misuse opioids. *Addict Sci Clin Pract*. 2021;16(1):33.
38. Franz B, Dhanani LY, Miller WC. Rural-urban differences in physician bias toward patients with opioid use disorder. *Psychiatr Serv*. 2021;72(8):874-9.
39. Gano L, Renshaw SE, Hernandez RH, et al. Opioid overdose prevention in family medicine clerkships: a CERA study. *Fam Med.* 2018;50(9):698-701.
40. Gassman RA. Medical specialization, profession, and mediating beliefs that predict stated likelihood of alcohol screening and brief intervention: targeting educational interventions. *Subst Abus*. 2003;24(3):141-56.
41. Geirsson M, Bendtsen P, Spak F. Attitudes of Swedish general practitioners and nurses to working with lifestyle change, with special reference to alcohol consumption. *Alcohol Alcohol*. 2005;40(5):388-93.

© 2024 Campopiano von Klimo M et al. *JAMA Network Open.*

42. George M, Martin E. GP's attitudes towards drug users. *Br J Gen Pract*. 1992;42(360):302.
43. Glanz A. Findings of a national survey of the role of general practitioners in the treatment of opiate misuse: views on treatment. *Br Med J (Clin Res Ed).* 1986;293(6546):543-5.
44. Gokirmak M, Ozturk O, Bircan A, et al. The attitude toward tobacco dependence and barriers to discussing smoking cessation: a survey among Turkish general practitioners. *Int J Public Health*. 2010;55(3):177-83.
45. Gordon AJ, Liberto J, Granda S, et al. Outcomes of DATA 2000 certification trainings for the provision of buprenorphine treatment in the Veterans Health Administration. *Am J Addict*. 2008;17(6):459-62.
46. Graham DM, Maio RF, Blow FC, et al. Emergency physician attitudes concerning intervention for alcohol abuse/dependence delivered in the emergency department: a brief report. *J Addict Dis*. 2000;19(1):45-53.
47. Gunderson EW, Fiellin DA, Levin FR, et al. Evaluation of a combined online and in person training in the use of buprenorphine. *Subst Abus*. 2006;27(3):39-45.
48. Hammond CJ, Parhami I, Young AS, et al. Provider and practice characteristics and perceived barriers associated with different levels of adolescent SBIRT implementation among a national sample of US pediatricians. *Clin Pediatr (Phila)*. 2021;60(9-10):418-26.
49. Harris BR, Yu J. Attitudes, perceptions and practice of alcohol and drug screening, brief intervention and referral to treatment: a case study of New York State primary care physicians and non-physician providers. *Public Health*. 2016;139:70-78.
50. Hayes RW, Hanley K, Calvo-Friedman A, et al. Suboxone and me: an OSCE-based assessment of medical residents' knowledge of medical treatment of opioid addiction. *J Gen Intern Med*. 2018;33(2):349.
51. Hernandez-Meier J, Good S, Zosel A. Emergency medicine provider attitudes toward and experiences with prescribing buprenorphine in the ED of a large academic metropolitan hospital. *Clinical Toxicology*. 2019;57(10):1041-2.
52. Hernandez-Meier J, Li D, Zosel A. Emergency provider practices and attitudes around naloxone-prescribing in an academic emergency department. *Clinical Toxicology*. 2020;58(11):1265.
53. Hoffmann T, Voigt K, Kugler J, et al. Are German family practitioners and psychiatrists sufficiently trained to diagnose and treat patients with alcohol problems? *BMC Fam Pract*. 2019;20(1):115.
54. Holmqvist M, Hermansson U, Nilsen P. Towards increased alcohol intervention activity in Swedish occupational health services. *Int J Occup Med Environ Health*. 2008;21(2):179-87.
55. Huhn AS, Dunn KE. Why aren't physicians prescribing more buprenorphine? *J Subst Abuse Treat.* 2017;78:1-7.
56. James JR, Gordon LM, Klein JW, et al. Interest in prescribing buprenorphine among resident and attending physicians at an urban teaching clinic. *Subst Abus*. 2019;40(1):11-13.
57. Johansson K, Bendtsen P, Akerlind I. Early intervention for problem drinkers: readiness to participate among general practitioners and nurses in Swedish primary health care. *Alcohol Alcohol*. 2002;37(1):38-42.
58. Johnson E, Lam C, Bluthenthal R, et al. 211 Impact of X-waiver training on resident barriers and biases surrounding buprenorphine treatment for opiate use disorder. *Ann Emerg Med*. 2020;76(4):S82.
59. Johnson TP, Booth AL, Johnson P. Physician beliefs about substance misuse and its treatment: findings from a U.S. survey of primary care practitioners. *Subst Use Misuse*. 2005;40(8):1071-84.
60. Jones CM, McCance-Katz EF. Characteristics and prescribing practices of clinicians recently waivered to prescribe buprenorphine for the treatment of opioid use disorder. *Addiction*. 2019;114(3):471-82.

© 2024 Campopiano von Klimo M et al. *JAMA Network Open.*

61. Kaner EF, Heather N, Brodie J, et al. Patient and practitioner characteristics predict brief alcohol intervention in primary care. *Br J Gen Pract*. 2001;51(471):822-7.
62. Kaner EF, Heather N, McAvoy BR, et al. Intervention for excessive alcohol consumption in primary health care: attitudes and practices of English general practitioners. *Alcohol Alcohol*. 1999;34(4):559-66.
63. Karam-Hage M, Nerenberg L, Brower KJ. Modifying residents' professional attitudes about substance abuse treatment and training. *Am J Addict*. 2001;10(1):40-7.
64. Keller CE, Ashrafioun L, Neumann AM, et al. Practices, perceptions, and concerns of primary care physicians about opioid dependence associated with the treatment of chronic pain. *Subst Abus*. 2012;33(2):103-13.
65. Kennedy AJ, Carter A, McNeil M. Impact of attending a mutual support group meeting on resident trainee attitudes toward patients with substance use disorder. *J Gen Intern Med*. 2018;33(2):229-30.
66. Kennedy-Hendricks A, Busch SH, McGinty EE, et al. Primary care physicians' perspectives on the prescription opioid epidemic. *Drug Alcohol Depend*. 2016;165:61-70.
67. Kermack A, Flannery M, Tofighi B, et al. Buprenorphine prescribing practice trends and attitudes among New York providers. *J Subst Abuse Treat*. 2017;74:1-6.
68. Kershaw C, Stead W, Rowley CF. Educational intervention to improve communication with patients who have opioid use disorder. *Open Forum Infect Dis*. 2018;5(Suppl 1):S405.
69. Kestler A, Kaczorowski J, Dong K, et al. A cross-sectional survey on buprenorphine-naloxone practice and attitudes in 22 Canadian emergency physician groups: a cross-sectional survey. *CMAJ Open*. 2021;9(3):E864-E873.
70. Keto J, Jokelainen J, Timonen M, et al. Physicians discuss the risks of smoking with their patients, but seldom offer practical cessation support. *Subst Abuse Treat Prev Policy*. 2015;10:43.
71. Kiernon B, Cox J, Fletcher-Louis M, et al. Reducing consumption versus maintaining abstinence: market access challenges facing a novel treatment pathway for alcohol addiction in the EU5. *Value Health*. 2015;18(7):413.
72. Kirane H, Drits E, Ahn S, et al. Addressing the opioid crisis: assessment of clinicians in a large healthcare system. *J Addict Med*. 2018;12(3):E1.
73. Kissin W, McLeod C, Sonnefeld J, et al. Experiences of a national sample of qualified addiction specialists who have and have not prescribed buprenorphine for opioid dependence. *J Addict Dis*. 2006;25(4):91-103.
74. Ko JY, Tong VT, Haight SC, et al. Obstetrician–gynecologists' practice patterns related to opioid use during pregnancy and postpartum - United States, 2017. *J Perinatol*. 2020;40(3):412-21.
75. Kohan LR, Elmofty D, Pena I, et al. Presence of opioid safety initiatives, prescribing patterns for opioid and naloxone, and perceived barriers to prescribing naloxone: cross-sectional survey results based on practice type, scope, and location. *J Opioid Manag*. 2021;17(1):19-38.
76. Konfino J, Mejia R, Basombrio A. Knowledge, attitudes and practices regarding smoking and cessation advice: a survey of physicians in Buenos Aires. *J Gen Intern Med*. 2011;26:S108.
77. Koopman FA, Parry CD, Myers B, et al. Addressing alcohol problems in primary care settings: a study of general medical practitioners in Cape Town, South Africa. *Scand J Public Health*. 2008;36(3):298-302.
78. Kouyoumdjian FG, Patel A, To MJ, et al. Physician prescribing of opioid agonist treatments in provincial correctional facilities in Ontario, Canada: a survey. *PLoS One*. 2018;13(2):e0192431.
79. Kunins HV, Sohler NL, Roose RJ, et al. HIV provider endorsement of primary care buprenorphine treatment: a vignette study. *Fam Med*. 2009;41(10):722-8.
80. Kunze U, Haidinger G. Alcoholism and nutrition results of a survey among general practitioners. *Alcoholism*. 1997;33(1-2):35-43.

© 2024 Campopiano von Klimo M et al. *JAMA Network Open.*

81. Lacroix L, Thurgur L, Orkin AM, et al. Emergency physicians' attitudes and perceived barriers to the implementation of take-home naloxone programs in Canadian emergency departments. *CJEM*. 2018;20(1):46-52.
82. Langton D, Hickey A, Bury G, et al. Methadone maintenance in general practice: impact on staff attitudes. *Ir J Med Sci*. 2000;169(2):133-6.
83. Latorre JM, López-Torres J, Sanchez-Nuñez T, et al. Primary care doctors' perception of treatment demand and need for training in drug addiction issues. *Primary Care Commun*. 2007;12(1):33-41.
84. Le KB, Johnson JA, Seale JP, et al. Primary care residents lack comfort and experience with alcohol screening and brief intervention: a multi-site survey. *J Gen Intern Med*. 2015;30(6):790-6.
85. Levy S, Wiseblatt A, Straus JH, et al. Adolescent SBIRT practices among pediatricians in Massachusetts. *J Addict Med*. 2020;14(2):145-9.
86. Linn LS, Yager J. Factors associated with physician recognition and treatment of alcoholism. *West J Med*. 1989;150(4):468-72.
87. Logan G, Craen A, Drone E, et al. 85 Physician-perceived barriers to treating opiate use disorder in the emergency department. *Ann Emerg Med*. 2020;76(4):S33-S34.
88. Loheswaran G, Soklaridis S, Selby P, et al. Screening and treatment for alcohol, tobacco and opioid use disorders: a survey of family physicians across Ontario. *PLoS One*. 2015;10(4):e0124402.
89. Lowenstein M, Kilaru A, Perrone J, et al. Barriers and facilitators for emergency department initiation of buprenorphine: a physician survey. *Am J Emerg Med*. 2019;37(9):1787-90.
90. Macalino GE, Sachdev DD, Rich JD, et al. A national physician survey on prescribing syringes as an HIV prevention measure. *Subst Abuse Treat Prev Policy*. 2009;4:13.
91. Marcell AV, Halpern-Felsher B, Coriell M, et al. Physicians' attitudes and beliefs concerning alcohol abuse prevention in adolescents. *Am J Prev Med*. 2002;22(1):49-55.
92. Mark TL, Kranzler HR, Song X. Understanding US addiction physicians' low rate of naltrexone prescription. *Drug Alcohol Depend*. 2003;71(3):219-28.
93. Mark TL, Kranzler HR, Song X, et al. Physicians' opinions about medications to treat alcoholism. *Addiction*. 2003;98(5):617-26.
94. Martinez CP, Vakkalanka P, Ait-Daoud N. Pharmacotherapy for alcohol use disorders: Physicians' perceptions and practices. *Front Psychiatry*. 2016;7:182.
95. Martino JG, Smith SR, Rafie S, et al. Physician and pharmacist: attitudes, facilitators, and barriers to prescribing naloxone for home rescue. *Am J Addict*. 2019;29(1):65-72.
96. Matheson C, Porteous T, Van Teijlingen E, et al. Management of drug misuse: an 8-year follow-up survey of Scottish GPs. *Br J Gen Pract*. 2010;60(576):517-20.
97. May JA, Warltier DC, Pagel PS. Attitudes of anesthesiologists about addiction and its treatment: a survey of Illinois and Wisconsin members of the American society of anesthesiologists. *J Clin Anesth*. 2002;14(4):284-9.
98. Mayet S, Manning V, Williams A, et al. Impact of training for healthcare professionals on how to manage an opioid overdose with naloxone: effective, but dissemination is challenging. *Int J Drug Policy*. 2011;22(1):9-15.
99. McGillion J, Wanigaratne S, Feinmann C, et al. GPs' attitudes towards the treatment of drug misusers. *Br J Gen Pract*. 2000;50(454):385-6.
100. Meijer E, Chavannes NH. Lacking willpower? A latent class analysis of healthcare providers' perceptions of smokers' responsibility for smoking. *Patient Educ Couns*. 2021;104(3):620-6.
101. Meijer E, Van Der Kleij R, Chavannes N. What keeps healthcare professionals from advising their patients who smoke to quit? A large-scale cross-sectional study. *Tob Induc Dis*. 2018;16:196.

© 2024 Campopiano von Klimo M et al. *JAMA Network Open.*

102. Meijer E, Van Der Kleij RMJJ, Chavannes NH. Facilitating smoking cessation in patients who smoke: A large-scale cross-sectional comparison of fourteen groups of healthcare providers. *BMC Health Serv Res*. 2019;19:750.
103. Mellinger JL, Im GY, Winters A, et al. Provider attitudes and practices for alcohol screening, treatment and education in patients with liver disease. *Hepatology*. 2020;72(1 SUPPL):186A-187A.
104. Messina MP, D'Angelo A, Battagliese G, et al. Fetal alcohol spectrum disorders awareness in health professionals: implications for psychiatry. *Riv Psichiatr*. 2020;55(2):79-89.
105. Midmer D, Kahan M, Kim T, et al. Efficacy of a physicians' pocket guide about prenatal substance use: a randomized trial. *Subst Abus*. 2011;32(4):175-9.
106. Miller ER, Ramsey IJ, Tran LT, et al. How Australian general practitioners engage in discussions about alcohol with their patients: a cross-sectional study. *BMJ Open*. 2016;6(12):e013921.
107. Miller SI, Frances R. Psychiatrists and the treatment of addictions: perceptions and practices. *Am J Drug Alcohol Abuse*. 1986;12(3):187-97.
108. Miquel L, López-Pelayo H, Nuño L, et al. Barriers to implement screening for alcohol consumption in Spanish hypertensive patients. *Fam Pract*. 2018;35(3):295-301.
109. Mitchell MA, Broyles LM, Pringle JL, et al. Education for the mind and the heart? Changing residents' attitudes about addressing unhealthy alcohol use. *Subst Abus*. 2017;38(1):40-2.
110. Moatti JP, Souville M, Escaffre N, et al. French general practitioners' attitudes toward maintenance drug abuse treatment with buprenorphine. *Addiction*. 1998;93(10):1567-75.
111. Mony PK, Jayakumar S. Preparedness for tobacco control among postgraduate residents of a medical college in Bangalore. *Indian J Community Med*. 2011;36(2):104-8.
112. Myles ML, Ziobrowski H, Scott K, et al. Emergency physician knowledge, attitudes, and barriers to emergency department-delivered buprenorphine. *Ann Emerg Med*. 2020;76(4):S54.
113. Netherland J, Botsko M, Egan JE, et al. Factors affecting willingness to provide buprenorphine treatment. *J Subst Abuse Treat*. 2009;36(3):244-51.
114. Nygaard P, Paschall MJ, Aasland OG, et al. Use and barriers to use of screening and brief interventions for alcohol problems among Norwegian general practitioners. *Alcohol Alcohol*. 2010;45(2):207-12.
115. Ordean A, Forte M, Selby P, et al. Screening, brief intervention, and referral to treatment for prenatal alcohol use and cigarette smoking: a survey of academic and community health care providers. *J Addict Med*. 2020;14(4):e76-e82.
116. O'Rourke M, Richardson LD, Wilets I, et al. Alcohol-related problems: emergency physicians' current practice and attitudes. *J Emerg Med*. 2006;30(3):263-8.
117. Peckham AM, Niculete ME, Steinberg H, et al. A survey of prescribers' attitudes, knowledge, comfort, and fear of consequences related to an opioid overdose education and naloxone distribution program. *J Public Health Manag Pract*. 2018;24(4):310-17.
118. Pelet A, Besson J, Pécoud A, et al. Difficulties associated with outpatient management of drug abusers by general practitioners. A cross-sectional survey of general practitioners with and without methadone patients in Switzerland. *BMC Fam Pract*. 2005;6:51.
119. Peterson GM, Northeast S, Jackson SL, et al. Harm minimization strategies: opinions of health professionals in rural and remote Australia. *J Clin Pharm Ther*. 2007;32(5):497-504.
120. Porath-Waller A, Gereghty S, Robeson P. Perceptions among healthcare professionals of prescription drug misuse. *Drug Alcohol Depend*. 2015;156:e180.
121. Price JH, Jordan TR, Dake JA. Obstetricians and gynecologists' perceptions and use of nicotine replacement therapy. *J Community Health*. 2006;31(3):160-75.
122. Pulcinelli M, Mannaioni PF, Zerbetto R. Professional knowledge on the subject of drug addiction. First results of a survey. *Giornale di Medicina Militare*. 1978;128(1-2):1-9.
123. Punzal M, Santos P, Li X, et al. Current practices in naloxone prescribing upon hospital discharge. *J Opioid Manag*. 2019;15(5):357-61.

© 2024 Campopiano von Klimo M et al. *JAMA Network Open.*

124. Pytell J, Brady M, Buresh M, et al. A pilot office-based opioid treatment clinic in an internal medicine resident continuity practice: provider, staff, and patient outcomes. *J Gen Intern Med*. 2019;34(2):S784.
125. Ramos MM, Sebastian RA, Murphy M, et al. Adolescent substance use: assessing the knowledge, attitudes, and practices of a school-based health center workforce. *Subst Abus*. 2017;38(2):230-6.
126. Raupach T, Merker J, Hasenfuss G, et al. Knowledge gaps about smoking cessation in hospitalized patients and their doctors. *Eur J Cardiovasc Prev Rehabil*. 2011;18(2):334-41.
127. Richmond RL, Mendelsohn CP. Physicians' views of programs incorporating stages of change to reduce smoking and excessive alcohol consumption. *Am J Health Promot*. 1998;12(4):254-7.
128. Rock NL, Silsby HD. The attitudes of American physicians stationed with the United States Army, Europe, in regard to alcohol and drug abuse. *Mil Med*. 1975;140(11):781-3.
129. Rowland N, Maynard A, Kennedy P, et al. Doctors and alcohol screening--the gap between attitudes and action. *Health Educ J*. 1988;47(4):133-6.
130. Ruetsch C, Schoenberger C. Factors that influence decisions among MBHO network physicians to use office-based opioid treatment or to increase the number of opioid-dependent patients they treat: results from the OBOT attitude and intention physician survey. *Value Health*. 2009;12(3):A188.
131. Russell HA, Smith B, Sanders M, et al. Attending a biopsychosocially focused buprenorphine training improves clinician attitudes. *Front Psychiatry*. 2021;12:639826.
132. Samuels EA, Dwyer K, Mello MJ, et al. Emergency department-based opioid harm reduction: moving physicians from willing to doing. *Acad Emerg Med*. 2016;23(4):455-65.
133. Satre DD, McCance-Katz EF, Moreno-John G, et al. Using needs assessment to develop curricula for screening, brief intervention, and referral to treatment (SBIRT) in academic and community health settings. *Subst Abus*. 2012;33(3):298-302.
134. Savage T, Ross M. Barriers and attitudes reported by Canadian emergency physicians regarding the initiation of buprenorphine/naloxone in the emergency department for patients with opioid use disorder. *CJEM*. 2022;24(1):44-49.
135. Schaeffer TH, Strout TDS, Baumann MR, et al. Emergency prescribers and rescue naloxone: results of a health-system survey. *Acad Emerg Med*. 2016;23:S126.
136. Schulte B, Schmidt CS, Kuhnigk O, et al. Structural barriers in the context of opiate substitution treatment in Germany--a survey among physicians in primary care. *Subst Abuse Treat Prev Policy*. 2013;8:26.
137. Sell J, Visconti A. Harm reduction: assessing the educational needs of family medicine residents in care of persons who inject drugs. *Fam Med*. 2020;52(7):514-7.
138. Sheffer CE, Barone CP, Anders ME. Training health care providers in the treatment of tobacco use and dependence: pre- and post-training results. *J Eval Clin Pract*. 2009;15(4):607-13.
139. Shuey B, Lee D, Ugalde I, et al. Evaluation of resident physicians' knowledge of and attitudes towards prescribing buprenorphine for patients with opioid use disorder. *J Addict Med*. 2021;15(3):219-25.
140. Sinclair L, Long H, Geary S, et al. New York state emergency department providers' attitudes on naloxone distribution for treatment of opioid overdose: a preliminary study. *Acad Emerg Med*. 2014;21(5):S165-S166.
141. Spandorfer JM, Israel Y, Turner BJ. Primary care physicians' views on screening and management of alcohol abuse: inconsistencies with national guidelines. *J Fam Pract*. 1999;48(11):899-902.
142. Stone EM, Kennedy-Hendricks A, Barry CL, et al. The role of stigma in U.S. primary care physicians’ treatment of opioid use disorder. *Drug Alcohol Depend*. 2021;221:108627.
143. Strange RE. The federal physician's attitude toward alcoholism: a sampling of naval medical officers' opinions. *Mil Med*. 1971;136(12):881-3.

© 2024 Campopiano von Klimo M et al. *JAMA Network Open.*

144. Sullivan LE, Tetrault J, Bangalore D, et al. Training HIV physicians to prescribe buprenorphine for opioid dependence. *Subst Abus*. 2006;27(3):13-8.
145. Taylor LE, Runarsdottir V, Zampi A, et al. Would you consider prescribing syringes to injection drug users? Addiction Medicine Conference Survey. *J Addict Dis*. 2003;22(1):67-78.
146. Tello MA, Wakeman SE, Lipsitz SR, et al. A survey of attitudes around opioid use disorder and perceived barriers to providing buprenorphine maintenance treatment among outpatient primary care providers in an urban academic medical setting. *J Gen Intern Med*. 2019;34(2):S119.
147. Thomas CP, Wallack SS, Lee S, et al. Research to practice: adoption of naltrexone in alcoholism treatment. *J Subst Abuse Treat*. 2003;24(1):1-11.
148. Tiako MJNN, Mahmood SUB. Cardiac surgeons' practices, attitudes regarding addiction care and patients who use drugs. *J Addict Med*. 2020;14(6):e388.
149. Twardella D, Brenner H. Lack of training as a central barrier to the promotion of smoking cessation: a survey among general practitioners in Germany. *Eur J Public Health*. 2005;15(2):140-5.
150. Vader JP, Aufseesser M. Physicians and intravenous drug users: attitudes and opinions in the Canton of Vaud, Switzerland. *Int J Addict*. 1993;28(14):1587-99.
151. Van Boekel LC, Brouwers EPM, van Weeghel J, et al. Healthcare professionals' regard towards working with patients with substance use disorders: comparison of primary care, general psychiatry and specialist addiction services. *Drug Alcohol Depend*. 2014 Jan;134:92-8.
152. Waal H, Brekke M, Clausen T, et al. General practitioners' views on drug-assisted rehabilitation. *Tidsskr Nor Laegeforen*. 2012;132(16):1861-6.
153. Wakeman S, Baggett M, Campbell E, et al. Internal medicine residents' training in substance use disorders. *J Gen Intern Med*. 2013;28:S109-S110.
154. Wakeman SE, Baggett MV, Pham-Kanter G, et al. Internal medicine residents training in substance use disorders: a survey of the quality of instruction and residents self-perceived preparedness to diagnose and treat addiction. *Subst Abus*. 2013;34(4):363-70.
155. Wakeman SE, Kanter GP, Donelan K. Institutional substance use disorder intervention improves general internist preparedness, attitudes, and clinical practice. *J Addict Med*. 2017;11(4):308-14.
156. Wakeman SE, Pham-Kanter G, Donelan K. General internists' attitudes, practices and preparedness related to substance use disorder. *J Gen Intern Med*. 2016;31(2):S231-S232.
157. Wakeman SE, Pham-Kanter G, Donelan K. Attitudes, practices, and preparedness to care for patients with substance use disorder: results from a survey of general internists. *Subst Abus*. 2016;37(4):635-41.
158. Walley AY, Alperen JK, Cheng DM, et al. Office-based management of opioid dependence with buprenorphine: clinical practices and barriers. *J Gen Intern Med*. 2008;23(9):1393-8.
159. Walther M, Montse B, Silvia M, et al. Teaching hospital staff about hazardous drinking: the effect of a single intervention. *Alcohol Alcohol*. 2008;43(1):51-2.
160. Warburg MM, Cleary PD, Rohman M, et al. Residents' attitudes, knowledge, and behavior regarding diagnosis and treatment of alcoholism. *J Med Educ*. 1987;62(6):497-503.
161. Weinberger AH, Reutenauer EL, Vessicchio JC, et al. Survey of clinician attitudes toward smoking cessation for psychiatric and substance abusing clients. *J Addict Dis*. 2008;27(1):55-63.
162. West SL, Luck RS, Capps CF, et al. Alcohol/other drug problems screening and intervention by rehabilitation physicians. *Alcohol Treat Q*. 2009;27(3):280-93.
163. Wilson GB, Lock CA, Heather N, et al. Intervention against excessive alcohol consumption in primary health care: a survey of GPs' attitudes and practices in England 10 years on. *Alcohol Alcohol*. 2011;46(5):570-7.
164. Wilson JD, Spicyn N, Matson P, et al. Internal medicine resident knowledge, attitudes, and barriers to naloxone prescription in hospital and clinic settings. *Subst Abus*. 2016;37(3):480-7.

© 2024 Campopiano von Klimo M et al. *JAMA Network Open.*

165. Winograd RP, Davis CS, Niculete M, et al. Medical providers' knowledge and concerns about opioid overdose education and take-home naloxone rescue kits within Veterans Affairs health care medical treatment settings. *Subst Abus*. 2017;38(2):135-40.
166. Wolk CB, Doubeni CA, Klusaritz HA, et al. Perspectives and practice in the identification and treatment of opioid use, alcohol use, and depressive disorders. *Psychiatr Serv*. 2019;70(10):940-3.
167. Wombacher K, Harrington NG, Scott AM, et al. Communication apprehension mediates the effects of past experience discussing substance use on child and adolescent psychiatrists' self-efficacy. *Patient Educ Couns*. 2019;102(4):651-5.
168. Yan J, Xiao S, Ouyang D, et al. Smoking behavior, knowledge, attitudes and practice among health care providers in Changsha city, China. *Nicotine Tob Res*. 2008;10(4):737-44.
169. Zellman GL, Bell RM, Archie C, et al. Physician response to prenatal substance exposure. *Matern Child Health J*. 1999;3(1):29-38.
170. Zuckerman M, Kelly T, Heard K, et al. Physician attitudes on buprenorphine induction in the emergency department: results from a multistate survey. *Clin Toxicol (Phila)*. 2021;59(4):279-85.
171. Abraham TH, Lewis ET, Cucciare MA. Providers' perspectives on barriers and facilitators to connecting women veterans to alcohol-related care from primary care. *Mil Med*. 2017;182(9):e1888-e94.
172. Abram HS, McCourt WF. Interaction of physicians with emergency ward alcoholic patients. *Q J Stud Alcohol*. 1964;25:679-88.
173. Aira M, Kauhanen J, Larivaara P, et al. Factors influencing inquiry about patients' alcohol consumption by primary health care physicians: qualitative semi-structured interview study. *Fam Pract*. 2003;20(3):270-5.
174. Alanis-Hirsch K, Croff R, Ford JH, et al. Extended-release naltrexone: a qualitative analysis of barriers to routine use. *J Subst Abuse Treat*. 2016;62:68-73.
175. Allen B, Harocopos A, Chernick R. Substance use stigma, primary care, and the New York State Prescription Drug Monitoring Program. *Behav Med*. 2020;46(1):52-62.
176. Andraka-Christou B, Capone MJ. A qualitative study comparing physician-reported barriers to treating addiction using buprenorphine and extended-release naltrexone in U.S. office-based practices. *Int J Drug Policy*. 2018;54:9-17.
177. Arborelius E, Damström Thakker K. Why is it so difficult for general practitioners to discuss alcohol with patients? *Fam Pract*. 1995;12(4):419-22.
178. Bar-Zeev Y, Skelton E, Bonevski B, et al. Overcoming challenges to treating tobacco use during pregnancy - a qualitative study of Australian general practitioners barriers. *BMC Pregnancy Childbirth.* 2019;19(1):61.
179. Barry DT, Irwin KS, Jones ES, et al. Integrating buprenorphine treatment into office-based practice: a qualitative study. *J Gen Intern Med*. 2009;24(2):218-25.
180. Bartholomew JB, Bute JJ. Exploring internal medicine interns' educational experiences on opioid addiction: a narrative analysis. *Health Commun*. 2023;38(1):169-76.
181. Beich A, Gannik D, Malterud K. Screening and brief intervention for excessive alcohol use: qualitative interview study of the experiences of general practitioners. *BMJ*. 2002;325(7369):870.
182. Bell G, Cohen J, Cremona A. How willing are general practitioners to manage narcotic misuse? *Health Trends*. 1990;22(2):56-7.
183. Binswanger IA, Koester S, Mueller SR, et al. Overdose education and naloxone for patients prescribed opioids in primary care: a qualitative study of primary care staff. *J Gen Intern Med*. 2015;30(12):1837-44.
184. Blevins CE, Rawat N, Stein MD. Gaps in the substance use disorder treatment referral process: provider perceptions. *J Addict Med*. 2018;12(4):273-7.

© 2024 Campopiano von Klimo M et al. *JAMA Network Open.*

185. Bounthavong M, Suh K, Christopher MLD, et al. Providers' perceptions on barriers and facilitators to prescribing naloxone for patients at risk for opioid overdose after implementation of a national academic detailing program: a qualitative assessment. *Res Social Adm Pharm*. 2020;16(8):1033-40.
186. Chichetto NE, Mannes ZL, Allen MK, et al. HIV care provider perceptions and approaches to managing unhealthy alcohol use in primary HIV care settings: a qualitative study. *Addict Sci Clin Pract*. 2019;14(1):21.
187. Cunningham CO, Sohler NL, McCoy K, et al. Attending physicians' and residents' attitudes and beliefs about prescribing buprenorphine at an urban teaching hospital. *Fam Med*. 2006;38(5):336-40.
188. Dong KA, Lavergne KJ, Salvalaggio G, et al. Emergency physician perspectives on initiating buprenorphine/naloxone in the emergency department: a qualitative study. *J Am Coll Emerg Physicians Open*. 2021;2(2):e12409.
189. Edsall A, Dinh TTT, Mai PP, et al. Provider perspectives on integration of substance use disorder and HIV care in Vietnam: a qualitative study. *J Behav Health Serv Res*. 2021;48(2):274-86.
190. El-Shahawy O, Brown R, Elston Lafata J. Primary care physicians' beliefs and practices regarding e-cigarette use by patients who smoke: a qualitative assessment. *Int J Environ Res Public Health*. 2016;13(5).
191. Fraeyman J, Symons L, Van Royen P, et al. How to overcome hurdles in opiate substitution treatment? A qualitative study with general practitioners in Belgium. *Eur J Gen Pract*. 2016;22(2):134-40.
192. Gatewood AK, Van Wert MJ, Andrada AP, et al. Academic physicians' and medical students' perceived barriers toward bystander administered naloxone as an overdose prevention strategy. *Addict Behav*. 2016;61:40-6.
193. Green CA, McCarty D, Mertens J, et al. A qualitative study of the adoption of buprenorphine for opioid addiction treatment. *J Subst Abuse Treat*. 2014;46(3):390-401.
194. Haug NA, Bielenberg J, Linder SH, et al. Assessment of provider attitudes toward #naloxone on Twitter. *Subst Abus*. 2016;37(1):35-41.
195. Herzig K, Danley D, Jackson R, et al. Seizing the 9-month moment: addressing behavioral risks in prenatal patients. *Patient Educ Couns*. 2006;61(2):228-35.
196. Herzig K, Huynh D, Gilbert P, et al. Comparing prenatal providers' approaches to four different risks: alcohol, tobacco, drugs, and domestic violence. *Women Health*. 2006;43(3):83-101.
197. Holland TJ, Penm J, Dinh M, et al. Emergency department physicians' and pharmacists' perspectives on take-home naloxone. *Drug Alcohol Rev*. 2019;38(2):169-76.
198. Hunter SB, Dopp AR, Ober AJ, et al. Clinician perspectives on methadone service delivery and the use of telemedicine during the COVID-19 pandemic: a qualitative study. *J Subst Abuse Treat*. 2021;124:108288.
199. Hutchinson E, Catlin M, Andrilla CH, et al. Barriers to primary care physicians prescribing buprenorphine. *Ann Fam Med*. 2014;12(2):128-33.
200. Hutchinson E, Rosenblatt R. Understanding and overcoming barriers to office-based physicians' treatment of opioid addiction. *J Investig Med*. 2013;61(1):115.
201. Hyland K, Hammarberg A, Andreasson S, et al. Treatment of alcohol dependence in Swedish primary care: perceptions among general practitioners. *Scand J Prim Health Care*. 2021;39(2):247-56.
202. Johansson K, Bendtsen P, Akerlind I. Factors influencing GPs' decisions regarding screening for high alcohol consumption: a focus group study in Swedish primary care. *Public Health*. 2005;119(9):781-8.
203. Kaner E, Rapley T, May C. Seeing through the glass darkly? A qualitative exploration of GPs' drinking and their alcohol intervention practices. *Fam Prac*. 2006;23(4):481-7.

© 2024 Campopiano von Klimo M et al. *JAMA Network Open.*

204. Kennedy AJ, Carter A, McNeil M. Resident trainee reflections on patients with substance use disorder after attending a mutual support group meeting. *J Gen Intern Med*. 2018;33(2):737-8.
205. Kenny K, O'Carroll A. The use of psychotherapeutic interventions by primary care GPs in Ireland in the treatment of their methadone patients: a grounded theory study. *Ir J Med Sci*. 2012;181(1):43-8.
206. Kersnik J, Poplas Susic T, Kolsek M, et al. What may stimulate general practitioners to undertake screening and brief intervention for excess alcohol consumption in Slovenia? A focus group study. *J Int Med Res*. 2009;37(5):1561-9.
207. Ketterer F, Symons L, Lambrechts MC, et al. What factors determine Belgian general practitioners' approaches to detecting and managing substance abuse? A qualitative study based on the I-Change Model. *BMC Fam Pract*. 2014;15:119.
208. Kilgore EA, Waddell EN, Tannert Niang KM, et al. Provider attitudes and practices on treating tobacco dependence in New York City after 10 years of comprehensive tobacco control efforts. *J Prim Care Community Health*. 2021;12:2150132720957448.
209. Klimas J, Muench J, Wiest K, et al. Alcohol screening among opioid agonist patients in a primary care clinic and an opioid treatment program. *J Psychoactive Drugs*. 2015;47(1):65-70.
210. Lambrechts MC, Ketterer F, Symons L, et al. The approach taken to substance abuse by occupational physicians: a qualitative study on influencing factors. *J Occup Environ Med*. 2015;57(11):1228-35.
211. Lid TG, Nesvåg S, Meland E. When general practitioners talk about alcohol: exploring facilitating and hampering factors for pragmatic case finding. *Scand J Public Health*. 2015;43(2):153-8.
212. Lin C, Detels R. A qualitative study exploring the reason for low dosage of methadone prescribed in the MMT clinics in China. *Drug Alcohol Depend*. 2011;117(1):45-49.
213. Mabood N, Ali S, Dong KA, et al. Experiences of pediatric emergency physicians in providing alcohol-related care to adolescents in the emergency department. *Pediatr Emerg Care*. 2013;29(12):1260-5.
214. Mark TL, Kranzler HR, Poole VH, et al. Barriers to the use of medications to treat alcoholism. *Am J Addict*. 2003;12(4):281-94.
215. Martin A, Raja A, White B. Emergency department-initiated medication-assisted treatment: lessons learned one year after launch. *Acad Emerg Med*. 2019;26:S308.
216. Mathew TA, Shields AL, Imasheva A, et al. Knowledge, attitudes, and practices of physicians in Tomsk Oblast tuberculosis services regarding alcohol use among tuberculosis patients in Tomsk, Russia. *Cult Med Psychiatry*. 2009;33(4):523-37.
217. Mathis SM, Hagaman A, Hagemeier N, et al. Provider-patient communication about prescription drug abuse: a qualitative analysis of the perspective of prescribers. *Subst Abus*. 2020;41(1):121-31.
218. May FC, Stocks N, Barton C. Identification of barriers that impede the implementation of nicotine replacement therapy in the acute cardiac care setting. *Eur J Cardiovasc Prev Rehabil*. 2008;15(6):646-50.
219. McCambridge J, Platts S, Whooley D, et al. Encouraging GP alcohol intervention: pilot study of change-orientated reflective listening (CORL). *Alcohol Alcohol*. 2004;39(2):146-9.
220. McKeown A, Matheson C, Bond C. A qualitative study of GPs' attitudes to drug misusers and drug misuse services in primary care. *Fam Pract*. 2003;20(2):120-5.
221. McMurphy S, Shea J, Switzer J, et al. Clinic-based treatment for opioid dependence: A qualitative inquiry. *Am J Health Behav*. 2006;30(5):544-54.
222. McNabb C, Foot C, Ting J, et al. Diagnosing drug-seeking behaviour in an adult emergency department. *Emerg Med Australas*. 2006;18(2):138-42.
223. McNeely J, Kumar P, Rieckmann T, et al. Barriers and facilitators affecting the implementation of substance use screening in primary care clinics: a qualitative study of patients, providers, and staff. *J Gen Intern Med*. 2017;32(2):S128.

© 2024 Campopiano von Klimo M et al. *JAMA Network Open.*

224. Meijer E, Kampman M, Geisler MS, et al. "It's on everyone's plate": a qualitative study into physicians' perceptions of responsibility for smoking cessation. *Subst Abuse Treat Prev Policy*. 2018;13(1):48.
225. Miller PM, Stockdell R, Nemeth L, et al. Initial steps taken by nine primary care practices to implement alcohol screening guidelines with hypertensive patients: the AA-TRIP project. *Subst Abus*. 2006;27(1-2):61-70.
226. Miner KJ, Holtan N, Braddock ME, et al. Barriers to screening and counseling pregnant women for alcohol use. *Minn Med*. 1996;79(10):43-7.
227. Molfenter T, Sherbeck C, Zehner M, et al. Implementing buprenorphine in addiction treatment: payer and provider perspectives in Ohio. *Subst Abuse Treat Prev Policy*. 2015;10:13.
228. Mudd J, Preston R, Larkins S. Qualitative exploration of barriers to alcohol management in patients with chronic disease in a regional setting. *Aust J Prim Health*. 2020;26(3):265-70.
229. Mules T, Taylor J, Price R, Walker L, Singh B, Newsam P, t al. Addressing patient alcohol use: a view from general practice. *J Prim Health Care*. 2012;4(3):217-22.
230. O'Donnell A, Kaner E. Are brief alcohol interventions adequately embedded in UK primary care? A qualitative study utilising normalisation process theory. *Int J Environ Res Public Health*. 2017;14(4).
231. Omole OB, Ayo-Yusuf OA, Ngobale KN. Implementing tobacco dependence treatment during clinical consultations: a qualitative study of clinicians' experiences, perceptions and behaviours in a South African primary health care setting. *BMC Fam Pract*. 2014;15:85.
232. Pflanz-Sinclair C, Matheson C, Bond CM, et al. Physicians' experiences of SBIRT training and implementation for SUD management in primary care in the UAE: a qualitative study. *Prim Health Care Res Dev*. 2018;19(4):344-54.
233. Pilnick A, Coleman T. 'Do your best for me': the difficulties of finding a clinically effective endpoint in smoking cessation consultations in primary care. *Health (London)*. 2010;14(1):57-74.
234. Poplas Susic T, Kersnik J, Kolsek M. Why do general practitioners not screen and intervene regarding alcohol consumption in Slovenia? A focus group study. *Wien Klin Wochenschr*. 2010;122(Suppl 2):68-73.
235. Quest TL, Rosenblatt R. Buprenorphine therapy for opioid addiction in rural Washington: the early adopters. *J Investig Med*. 2011;59(1):210-1.
236. Rahm AK, Boggs JM, Martin C, et al. Facilitators and barriers to implementing screening, brief intervention, and referral to treatment (SBIRT) in primary care in integrated health care settings. *Subst Abus*. 2015:36(3):281-8.
237. Rindal KE, Chin N, Shah MN, et al. There is no rock bottom: emergency physicians' approach and adaptations to alcohol intoxicated patients. *Ann Emerg Med*. 2012;60(4):S146.
238. Scarborough J, Eliott J, Braunack-Mayer A. Opioid substitution therapy: a study of GP participation in prescribing. *Aust Fam Physician*. 2011;40(4):241-5.
239. Tam CW, Zwar N, Markham R. Australian general practitioner perceptions of the detection and screening of at-risk drinking, and the role of the AUDIT-C: a qualitative study. *BMC Fam Pract*. 2013;14:121.
240. Taylor P, Zaichkin J, Pilkey D, et al. Prenatal screening for substance use and violence: findings from physician focus groups. *Matern Child Health J*. 2007;11(3):241-7.
241. Tesema L, Marshall J, Hathaway R, et al. Training in office-based opioid treatment with buprenorphine in US residency programs: a national survey of residency program directors. *Subst Abus*. 2018;39(4):434-40.
242. Todd FC, Sellman JD, Robertson PJ. Barriers to optimal care for patients with coexisting substance use and mental health disorders. *Aust N Z J Psychiatry*. 2002;36(6):792-9.
243. Van Hook S, Harris SK, Brooks T, et al. The "six T's": barriers to screening teens for substance abuse in primary care. *J Adolesc Health*. 2007;40(5):456-61.

© 2024 Campopiano von Klimo M et al. *JAMA Network Open.*

244. Van Hout MC, Crowley D, McBride A, et al. Optimising treatment in opioid dependency in primary care: results from a national key stakeholder and expert focus group in Ireland. *BMC Fam Pract*. 2018;19(1):103.
245. van Schayck OCP, Bindels L, Nijs A, et al. The experience of general practitioners with Very Brief Advice in the treatment of tobacco addiction. *NPJ Prim Care Respir Med*. 2020;30(1):40.
246. Varley AL, Lappan S, Jackson J, et al. Understanding barriers and facilitators to the uptake of best practices for the treatment of co-occurring chronic pain and opioid use disorder. *J Dual Diagn*. 2020;16(2):239-49.
247. Webster D, Meyer T, Crain C, et al. Emergency physician attitudes on opioid use disorder and barriers to providing buprenorphine/naloxone. *CJEM*. 2020;22:S46.
248. Wiercigroch D, Hoyeck P, Sheikh H, et al. Management of opioid withdrawal: a qualitative examination of current practices and barriers to prescribing buprenorphine in a Canadian emergency department. *CJEM*. 2019;21:S114.
249. Wiercigroch D, Hoyeck P, Sheikh H, et al. A qualitative examination of the current management of opioid use disorder and barriers to prescribing buprenorphine in a Canadian emergency department. *BMC Emerg Med*. 2021;21(1):48.
250. Williams EC, Achtmeyer CE, Young JP, et al. Barriers to and facilitators of alcohol use disorder pharmacotherapy in primary care: a qualitative study in five VA clinics. *J Gen Intern Med*. 2018;33(3):258-67.
251. Włoch K. Role of the countryside general practictioner in recognition of reasons and circumstances of alcohol abuse by patients of Spzoz. Part I. Characteristics of staff and patients resources in the outpatient health care in the Lublin voivodeship. *Wiad Lek*. 2002;55(Suppl 1 Pt 2):967-71.
252. Wolf I, Chafetz ME, Blane HT, et al. Social factors in the diagnosis of alcoholism. II. Attitudes of physicians. *Q J Stud Alcohol*. 1965;26:72-9.
253. Chenworth M, Perrone J, Love JS, et al. Buprenorphine initiation in the emergency department: a thematic content analysis of a #firesidetox Tweetchat. *J Med Toxicol*. 2020;16(3):262-8.
254. Foster SD, Lee K, Edwards C, et al. Providing incentive for emergency physician X-waiver training: an evaluation of program success and postintervention buprenorphine prescribing. *Ann Emerg Med*. 2020;76(2):206-14.
255. Gordon AJ, Ettaro L, Rodriguez KL, et al. Provider, patient, and family perspectives of adolescent alcohol use and treatment in rural settings. *J Rural Health*. 2011;27(1):81-90.
256. Haffajee RL, Andraka-Christou B, Attermann J, et al. A mixed-method comparison of physician-reported beliefs about and barriers to treatment with medications for opioid use disorder. *Subst Abuse Treat Prev Policy*. 2020;15:69.
257. Hanna EZ. Attitudes toward problem drinkers, revisited: patient-therapist factors contributing to the differential treatment of patients with alcohol problems. *Alcohol Clin Exp Res*. 1991;15(6):927-31.
258. Harris AHS, Ellerbe L, Rachelle N, et al. Pharmacotherapy for alcohol dependence: perceived treatment barriers and action strategies among Veterans Health Administration service providers. *Psychol Serv*. 2013;10(4):410-19.
259. Harutyunyan A, Abrahamyan A, Hayrumyan V, et al. Perceived barriers of tobacco dependence treatment: a mixed-methods study among primary healthcare physicians in Armenia. *Prim Health Care Res Dev*. 2019;20:e17.
260. Hawk KF, D'Onofrio G, Chawarski MC, et al. Barriers and facilitators to clinician readiness to provide emergency department-initiated buprenorphine. *JAMA Netw Open*. 2020;3(5):e204561.
261. Im DD, Chary A, Condella AL, et al. Emergency department clinicians' attitudes toward opioid use disorder and emergency department-initiated buprenorphine treatment: a mixed-methods study. *West J Emerg Med*. 2020;21(2):261-71.

© 2024 Campopiano von Klimo M et al. *JAMA Network Open.*

262. Joudrey P, Oldfield B, Yonkers KA, et al. A pre-implementation study of hospital-based health professional perspectives on prescribing of medications for alcohol use disorder. *J Gen Intern Med*. 2019;34(2):S113-S4.
263. Joudrey PJ, Oldfield BJ, Yonkers KA, et al. Inpatient adoption of medications for alcohol use disorder: a mixed-methods formative evaluation involving key stakeholders. *Drug Alcohol Depend*. 2020;213:108090.
264. Kathuria H, Seibert RG, Cobb V, et al. Patient and physician perspectives on treating tobacco dependence in hospitalized smokers with substance use disorders: a mixed methods study. *J Addict Med*. 2019;13(5):338-45.
265. Matusow H, Rosenblum A, Fong C, et al. Factors associated with mental health clinicians' referrals to 12-step groups. *J Addict Dis*. 2012;31(3):303-12.
266. McCausland K, Haider B, White M, et al. Current practice in withdrawal management: opportunities to improve treatment of opioid use disorder. *Am J Addict*. 2020;29(3):202-3.
267. Oros SM, Christon LM, Barth KS, et al. Facilitators and barriers to utilization of medications for opioid use disorder in primary care in South Carolina. *Int J Psychiatry Med*. 2021;56(1):14-39.
268. Palmer A, Karakus M, Mark T. Barriers faced by physicians in screening for substance use disorders among adolescents. *Psychiatr Serv*. 2019;70(5):409-12.
269. Panda R, Persai D, Mathur M, et al. Perception and practices of physicians in addressing the smokeless tobacco epidemic: findings from two states in India. *Asian Pac J Cancer Prev*. 2013;14(12):7237-41.
270. Penm J, MacKinnon NJ, Lyons MS, et al. Combatting opioid overdoses in Ohio: emergency department physicians’ prescribing patterns and perceptions of naloxone. *J Gen Intern Med*. 2018;33(5):608-9.
271. Quest TL, Merrill JO, Roll J, et al. Buprenorphine therapy for opioid addiction in rural Washington: the experience of the early adopters. *J Opioid Manag*. 2012;8(1):29-38.
272. Roche AM, Richard GP. Doctors' willingness to intervene in patients' drug and alcohol problems. *Soc Sci Med*. 1991;33(9):1053-61.
273. Rohman ME, Cleary PD, Warburg M, et al. The response of primary care physicians to problem drinkers. *Am J Drug Alcohol Abuse*. 1987;13(1-2):199-209.
274. Rowan MS, Galasso CS. Identifying office resource needs of Canadian physicians to help prevent, assess and treat patients with substance use and pathological gambling disorders. *J Addict Dis*. 2000;19(2):43-58.
275. Segnan N, Battista RN, Rosso S, et al. Preventive practices of general practitioners in Torino, Italy. *Am J Prev Med*. 1992;8(6):333-8.
276. Stöver H. Barriers to opioid substitution treatment access, entry and retention: a survey of opioid users, patients in treatment, and treating and non-treating physicians. *Eur Addict Res*. 2011;17(1):44-54.
277. Van Zyl PM. Doctors' views of disulfiram and their response to relapse in alcohol-dependent patients, Free State, 2009. *Afr J Prim Health Care Fam Med*. 2016;8(1):e1-e7.
278. Wamsley MA, Gleason N, Guy M, et al. Teaching residents screening, brief intervention and referral to treatment skills for alcohol use: using chart-stimulated recall to assess curricular impact. *J Gen Intern Med*. 2014;29:S536.
279. Yilani M, Chakraborty A, Goyes D, et al. Medication-assisted treatment in alcohol use disorder: can education and EMR interventions increase prescriptions? *Am J Addict*. 2020;29(3):213-4.
280. Anderson P, Kaner E, Wutzke S, et al. Attitudes and managing alcohol problems in general practice: an interaction analysis based on findings from a WHO collaborative study. *Alcohol Alcohol*. 2004;39(4):351-6.
281. Mitchell AJ, Meader N, Bird V, et al. Clinical recognition and recording of alcohol disorders by clinicians in primary and secondary care: meta-analysis. *Br J Psychiatry*. 2012;201:93-100

© 2024 Campopiano von Klimo M et al. *JAMA Network Open.*

282. Mertens J, Weisner C, Sterling S, et al. Structual and attitudinal factors affecting sbirt implementation in adult primary care. *Alcoholism: Clinical and Experimental Research*. 2011;35:285A.
283. Muench J, Jarvis K, Vandersloot D, et al. Perceptions of clinical team members toward implementation of SBIRT processes. *Alcohol Treat Q*. 2015;33(2):143-60.

© 2024 Campopiano von Klimo M et al. *JAMA Network Open.*
