# Supplementary material for: Physician Reluctance to Intervene in Addiction: A Systematic Review
Source: JAMA Netw Open. 2024 Jul 17;7(7):e2420837. doi: 10.1001/jamanetworkopen.2024.20837 (PMC11255913; doi:10.1001/jamanetworkopen.2024.20837)
Supplement: Supplement 2. — Data Sharing Statement [file jamanetwopen-e2420837-s002.pdf]

Data Sharing Statement

Campopiano von Klimo. Physician Reluctance to Intervene in Addiction. *JAMA Netw Open*. Published July 17, 2024. doi:10.1001/jamanetworkopen.2024.20837

Data
Data available: No

Additional Information
Explanation for why data not available: We have provided the search terms and a list of all included papers in the publication. If any additional information is needed by others, we will be glad to provide it upon request.
